# Genome Mining of Marine-Derived *Streptomyces* sp. SCSIO 40010 Leads to Cytotoxic New Polycyclic Tetramate Macrolactams

**DOI:** 10.3390/md17120663

**Published:** 2019-11-25

**Authors:** Wei Liu, Wenjun Zhang, Hongbo Jin, Qingbo Zhang, Yuchan Chen, Xiaodong Jiang, Guangtao Zhang, Liping Zhang, Weimin Zhang, Zhigang She, Changsheng Zhang

**Affiliations:** 1 South China Sea Resource Exploitation and Protection Collaborative Innovation Center (SCS-REPIC)/School of Marine Sciences, Sun Yat-sen University, Guangzhou 510006, Chinacesshzhg@mail.sysu.edu.cn (Z.S.); 2 Key Laboratory of Tropical Marine Bio-resources and Ecology, Guangdong Key Laboratory of Marine Materia Medica, South China Sea Institute of Oceanology, Innovation Academy for South China Sea Ecology and Environmental Engineering, Chinese Academy of Sciences, 164 West Xingang Road, Guangzhou 510301, China; wzhang@scsio.ac.cn (W.Z.); khbmail@126.com (H.J.); gudaobo@163.com (Q.Z.); jxd374487986@163.com (X.J.); gtzhang@scsio.ac.cn (G.Z.); zhanglp@scsio.ac.cn (L.Z.); 3 State Key Laboratory of Applied Microbiology Southern China, Guangdong Institute of Microbiology, 100 Central Xianlie Road, Guangzhou 510070, China; chenyc@gdim.cn (Y.C.); wmzhang@gdim.cn (W.Z.)

**Keywords:** *Streptomyces* sp. SCSIO 40010, marine, genome mining, polycyclic tetramate macrolactams, cytotoxicity

## Abstract

Polycyclic tetramate macrolactams (PTMs) biosynthetic gene cluster are widely distributed in different bacterial types, especially in *Streptomyces* species. The mining of the genomic data of marine-derived *Streptomyces* sp. SCSIO 40010 reveals the presence of a putative PTM-encoding biosynthetic gene cluster (*ptm′* BGC) that features a genetic organization for potentially producing 5/5/6 type of carbocyclic ring-containing PTMs. A fermentation of *Streptomyces* sp. SCSIO 40010 led to the isolation and characterization of six new PTMs **1**–**6**. Comprehensive spectroscopic analysis assigned their planar structures and relative configurations, and their absolute configurations were deduced by comparing the experimental electronic circular dichroism (ECD) spectra with the reported spectra of the known PTMs. Intriguingly, compounds **1**–**6** were determined to have a *trans*-orientation of H-10/H-11 at the first 5-membered ring, being distinct from the *cis*-orientation in their known PTM congeners. PTMs **1**–**5** displayed cytotoxicity against several cancer cell lines, with IC_50_ values that ranged from 2.47 to 17.68 µM.

## 1. Introduction

Polycyclic tetramate macrolactams (PTMs) are a unique class of natural products that consist of a tetramate-embedding macrocyclic lactam core and a varying carbocycle with 5/6, 5/5, 5/6/5, or 5/5/6 ring system [1]. PTMs display a wide range of antifungal, antibiotic, antiprotozoal, and antitumor properties [2,3,4,5], and they have significant potential for applications in agricultures and medicines [1,6]. HSAF (also known as dihydromaltophilin) [7], a typical representative of 5/5/6 type of PTMs, exhibits a broad spectrum of antifungal activities and it has been used as an antifungal agent to control plant diseases [8]. The anticancer agent ikarugamycin [9], a typical 5/6/5 type of PTMs, shows activity as an inhibitor of clathrin-mediated endocytosis [10]. Therefore, PTMs draw the attention of synthetic chemists; however, multiple chiral centers in PTMs greatly enhance the structure diversity and increase the difficulty for the total synthesis [11,12,13,14]. In a sharp contrast, in nature, a conserved and compact biosynthetic pathway has been evolved to simply assemble such kinds of complex structures [1]. Recent studies reveal that PTMs are derived from a conserved hybrid polyketide synthase (PKS)/non-ribosomal peptide synthethase (NRPS) pathway [1,15]. The PKS portion of the hybrid PKS/NRPS enzyme is iteratively used to produce two separate polyketide chains, which are respectively condensed with the *α*- and *δ*-amino groups of an l-orinithine that is tethered in the NRPS portion to generate a common polyene tetramate precursor [7,15,16,17,18,19,20,21]. Afterwards, a set of oxidoreductases catalyzes divergent cyclization reactions to control the formation of diverse carbocyclic ring systems in PTMs [18,20,22,23,24]. Particularly, the iterative assembly of two separate polyketide chains by the same single-module bacterial polyketide synthase has been demonstrated in vitro in the biosynthesis of HSAF [16], and the biocatalytic total synthesis of ikarugamycin has been recently achieved [22].

**Figure 1:**
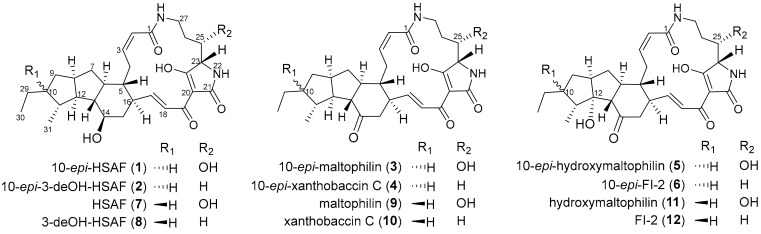
Chemical structures of polycyclic tetramate macrolactams (PTMs). Compounds **1**–**6** were isolated from *Streptomyces* sp. SCSIO 40010. The known compounds **7**–**12** with the same planar structures as those of **1–6**, respectively, are shown here for comparison.

Genome Mining has been successfully utilized to discover new PTMs [9,15,17,20,25,26]. For example, we have reported the activation of a silent PTM biosynthetic gene cluster (BGC) by promoter engineering in marine-derived *Streptomyces pactum* SCSIO 02999 to produce a series of new PTMs pactamides with a 5/5/6 ring system [20]. In addition, we have characterized three new PTMs containing a 5/6/5 ring system from a South China Sea-derived *Streptomyces* sp. SCSIO 40060 while using a genomics-guided approach [26]. We identified a mangrove-derived *Streptomyces* sp. SCSIO 40010 harboring a putative PTM BGC during our continuous search for PTM-producing strains. Herein, we reported the isolation, structural elucidation and biological evaluation of six new PTMs **1**–**6** (Figure 1).

## 2. Results and Discussion

### 2.1. Genome Mining of a PTM Biosynthetic Gene Cluster

**Figure 2:**
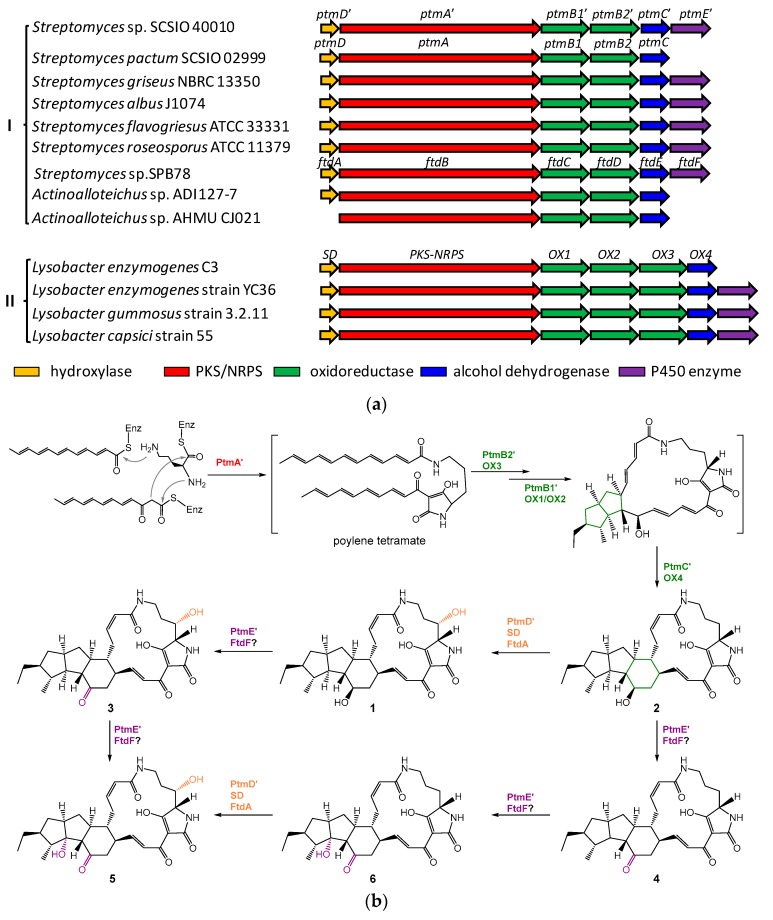
(**a**) Bioinformatics analysis of 5/5/6 type of PTM biosynthetic gene clusters (BGCs). (**b**) The proposed biosynthetic pathway for six new 5/5/6 type of PTMs.

The strain SCSIO 40010 was isolated from the mangrove sediment in Penang, Malaysia, and it was identified to be a *Streptomyces* species on the basis of its 16S rDNA sequence (GenBank accession number MN224032). The mining of the sequenced genome of *Streptomyces* sp. SCSIO 40010 reveals the presence of a putative PTM BGC (*ptm*′, GenBank accession number MN234160) that displays high similarity to the *ptm* BGC in *S. pactum* SCSIO 02999 (Figure 2a) [20]. This *ptm′* BGC encodes six conserved enzymes, including the hybrid PKS/NRPS PtmA′, the FAD-dependent oxidoreductase PtmB1′, PtmB2′, the alcohol dehydrogenase PtmC′, the hydroxylase PtmD′, and the P450 enzyme PtmE′. In addition to the scaffold constructing enzymes PtmA′, PtmB1′, PtmB2′, and PtmC′, two modifying enzymes PtmD′ (resembling the C-25 hydroxylase FtdA [15], 63% identity) and PtmE′ (resembling the P450 enzyme FtdF [15], 59% identity) were also found in the *ptm′* BGC in *Streptomyces* sp. SCSIO 40010.

Our preliminary genome mining of *Streptomyces* sp. SCSIO 40010 indicates that it should be a potential producer of PTMs with a 5/5/6 carbocyclic ring system [1,15]. Thus, we mined the available genome sequences for PTM BGCs and made a bioinformatics analysis of the PTM BGCs, typically for 5/5/6 type of PTMs [1,15]. Our analysis shows that the BGCs for 5/5/6 type of PTMs should fall into two categories (Figure 2a), depending on the number of oxidoreductases that are involved in the construction of the 5/5 ring system (two for Group I and three for Group II).

The PTM BGCs of Group I are mainly distributed in *Streptomyces* species (Figure 2a). Some of these *Streptomyces* strains have been demonstrated to produce 5/5/6 and/or 5/5 type of PTMs, such as pactamides in *S. pactum* SCSIO 02999 [20], compounds a–d in *S. griseus* NBRC 13350 [17], alteramides in *S. albus* J1074 [25], and frontalamides in *Streptomyces* sp. SPB78 [15]. In contrast, no PTMs have been reported from *S. flavogriseus* ATCC 33331 and *S. roseosporus* ATCC 11379 that contain Group I of PTM BGCs (Figure 2a) [15]. In addition to *Streptomyces* species, *Actinoalloteichus* sp. ADI127-7 and AHMU CJ201 also contain Group I of PTM BGCs (Figure 2a), while no PTMs have been reported from them. *Actinoalloteichus cyanogriseus* WH1-2216-6 was reported to produce HSAF and its analogues [27]; however, its genome sequence is not yet publicly available.

Biosynthetically, it has been experimentally demonstrated in *S. pactum* SCSIO 02999 that two oxidoreductases PtmB1 and PtmB2 are responsible for the sequential formation of the 5/5 ring system in pactamide A, with the formation of the first 5-membered ring by PtmB2 and the second 5-membered ring by PtmB1 [20]. Subsequently, formation of the inner 6-membered is catalyzed by the alcohol dehydrogenase PtmC [20].

The PTM BGCs of Group II are mainly found in bacterial strains of *Lysobacter* (Figure 2a). The strains *Lysobacter enzymogenes* C3 and strain YC36 are validated to produce HSAF and related PTMs [7,16,19,23]. The strains *Lysobacter gummosus* strain 3.2.11 and *Lysobacter capsici* strain 55 are indicated as HSAF producers [28]. Unlike Group I PTM BGCs, three oxidoreductases (such as OX1, OX2, and OX3 in *Lysobacter enzymogenes*) from the PTM BGCs of Group II are involved in the formation of the 5/5 ring system [23]. It has been shown that OX3 is responsible for the first 5-membered ring formation in lysobacterene A, while OX1 and OX2 catalyze the formation of the second 5-membered ring, but with different stereo selectivity [23]. In a similar fashion to PtmC, OX4 catalyzes the formation of the inner 6-membered ring in HSAF (**7**, Figure 1) [23,24].

The metabolite profiles of *Streptomyces* sp. SCSIO 40010 were investigated by cultivation in four different media, including modifed-A1BFe+C [29], AM6, AM6-4, and modifed-ISP3 [26]. HPLC analyses showed that compounds exhibiting UV-visible absorption spectra that were similar to PTMs were better produced in the modifed-A1BFe+C medium (Supplementary Figure S1). Subsequently, a 20-L fermentation of *Streptomyces* sp. SCSIO 40010 was performed in the modifed-A1BFe+C. Butanone extracts of the 20-L fermentation cultures were subjected to multiple chromatographic methods to provide six new PTMs 1–6.

**Figure 3:**
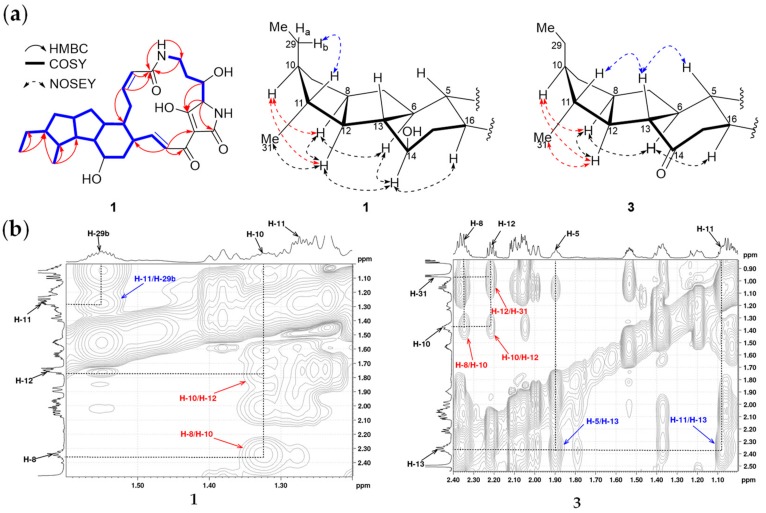
(**a**) Key COSY, HMBC correlations for **1,** and selected NOE correlations for **1** and **3**. (**b**) Key NOE correlations to support a *trans*-orientation of H-10/H-11 in **1** and **3**.

Compound **1** was isolated as a white powder. The molecular formula of **1** was determined as C_29_H_40_N_2_O_6_ by HRESIMS ([M + H]^+^, *m*/*z* 513.2960, calcd for 513.2965, Supplementary Figure S2). The planar structure of **1** was determined to be the same as that of HSAF (**7**, Figure 1) [27,30], by comparing NMR spectroscopic data of **1** (Table 1 and Table 2; Supplementary Figures S3–S9) and HSAF (**7**) [27,30]. The geometries of double bonds in **1** were determined to be *trans* (*E*) or *cis* (*Z*) on the basis of their coupling constants (*Z*∆^2,3^ *J*_2,3_ 11.5 Hz; *E*∆^17,18^ *J*_17,18_ 15.5 Hz; Table 1). The relative configurations of **1** were assigned by NOESY correlations and then compared with pactamide A [20] and HSAF (**7**) [30] (Figure 3a). It should be noted that a *trans*-orientation of H-10 and H-11 was assigned for **1**, because of the obvious NOESY correlations of H-8/H-10, H10/H-12, and H-11/H-29b (Figure 3). Previously, a *trans*-orientation of H-10 and H-11 was reported for pactamide A [20], aburatubolactam A (X-ray crystallography structure available [31]), combamide D [32], deOH alteramides, and lysobacterene B [33]. In the recently reported 5/5 type of PTMs umezawamides, the relative orientation of H-10 and H-11 was not determined [34]. However, a *cis*-orientation of H-10 and H-11 was determined in **7** [30]. As described by Cao et al. [9] and Hoshino et al. [34], the small vicinal coupling constant between H-23 and H-25 strongly indicated the relative configuration between H-23 and H-25 to be (23S*, 25S*) in **1** (^3^*J*_H-23/H-25_ 1.2 Hz, Table 1). The configuration of C-23 was deduced to be 23*S* upon the proposed biogenesis from an l-orinithine [7,15,16,17,18,19,20,21]. Recently, a crystallographic study unequivocally determined the absolute configuration as 23*S*, 25*S* in hydroxylikarugamycin A [26]. Thus, when considering the biosynthetic similarity between **1** and hydroxyikarugamycin A, **1** was deduced to also have the stereochemistry of 23*S*, 25*S*. Given that **1** and **7** (5*R*, 6*S*, 8*S*, 10*R*, 11*R*, 12*R*, 13*S*, 14*R*, 16*R*, 23*S*, 25*S* [30]) displayed an almost identical electronic circular dichroism (ECD) spectra (Supplementary Figure S10), the absolute configuration of **1** was deduced as 5*R*, 6*S*, 8*S*, 10*S*, 11*R*, 12*R*, 13*S*, 14*R*, 16*R*, 23*S*, 25*S*, which was only different from **7** by adopting an opposite configuration at C-10 (Figure 1). Thus, compound **1** was designated 10-*epi*-HSAF.

**Figure 4:**
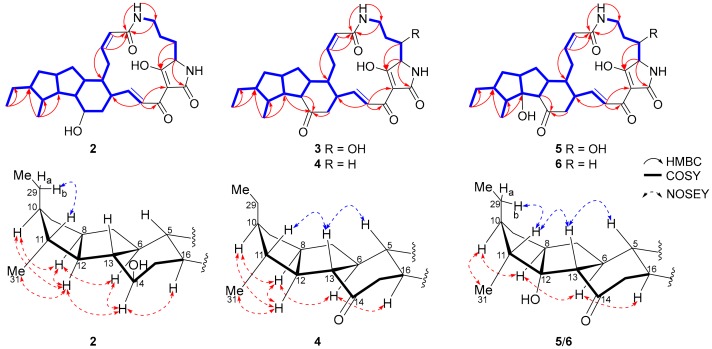
Key COSY, HMBC, and selected NOE correlations for **2**–**6**.

Compound **2** was obtained as a white powder and it was assigned the molecular formula of C_29_H_40_N_2_O_5_ on the basis of HRESIMS ([M + H]^+^, *m*/*z* 497.3010, calcd for 497.3015, Supplementary Figure S11). A detailed comparison of one-dimensional (1D) and two-dimensional (2D) NMR spectroscopic data of **2** (Table 1 and Table 2, Supplementary Figures S12–S18) and deOH-HSAF (**8**, Figure 1) revealed the same planar structure for **2** and **8** [27,30]. The relative configuration of **2** was deduced by proton coupling constants (*Z*∆^2,3^ *J*_2,3_ 11.5 Hz; *E*∆^17,18^ *J*_17,18_ 15.5 Hz; Table 1) and careful analysis of NOESY correlations (Figure 4, Supplementary Figures S17 and S18). Similar to **1**, a *trans*-orientation of H-10/H-11 was determined in **2** (Figure 1), by deducing from NOESY correlations of H-8/H-10, H10/H-12, H12/Me-31, and H-11/H-29b. This was different from the *cis*-orientation of H-10/H-11 in **8** (Figure 1) [30]. **2** was deduced to have the absolute configuration of 5*R*, 6*S*, 8*S*, 10*S*, 11*R*, 12*R*, 14*R*, 13*S*, 16*R*, and 23*S* because of the almost identical ECD spectra of **1** and **8** (Supplementary Figure S10), and thus compound **2** was designated 10-*epi*-deOH-HSAF.

Compound **3** was isolated as a reddish and amorphous powder. The molecular formula of **3** was determined as C_29_H_38_N_2_O_6_ by HRESIMS ([M−H]^−^, *m*/*z* 509.2642, calcd for 509.2657, Supplementary Figure S19). Careful analysis of the 1D and 2D NMR data of **3** (Table 1 and Table 2, Supplementary Figures S20–S26) revealed that **3** was an isomer of maltophilin (**9**, Figure 1) [27]. The *trans*-orientation of H-10/H-11 in **3**, which differed from the *cis*-orientation of H-10/H-11 in **9**, was supported by NOESY correlations of H-8/H-10, H-10/H-12, and H-12/Me-31 (Figure 3, Supplementary Figures S25 and S26). When considering the similar ECD spectra of **3** and **1** (Supplementary Figure S10), the absolute configuration of **3** was deduced as 5*R*, 6*S*, 8*S*, 10*S*, 11*R*, 12*R*, 13*S*, 16*R*, 23*S*, and 25*S*, and thus **3** was designated 10-*epi*-maltophilin.

Compound **4** was obtained as a white powder and it was assigned the molecular formula as C_29_H_38_N_2_O_5_ by HRESIMS ([M + H]^+^, *m*/*z* 495.2846, calcd for 495.2859, Supplementary Figure S27). Detailed comparison of NMR spectroscopic data of **4** (Table 1 and Table 2, Supplementary Figures S28–S34) and xanthobaccin C (**10**, Figure 1) uncovered that **4** was an isomer of **10** [27]. The key NOESY correlations of H-8/H-10, H-10/H-12, and H-12/Me-31 in **4** (Figure 4, Supplementary Figure S34) supported a *trans*-orientation of H-10/H-11 in **4**. The absolute configuration of **4** was deduced as 5*R*, 6*S*, 8*S*, 10*S*, 11*R*, 12*R*, 13*S*, 16*R*, and 23*S* by comparing the ECD spectra of **4** and **2** (Supplementary Figure S10). Therefore, **4** was designated 10-*epi*-xanthobaccin C.

Compound **5** was obtained as a reddish powder. The molecular formula of **5** was assigned as C_29_H_38_N_2_O_7_ by HRESIMS ([M + H]^+^, *m*/*z* 527.2757, calcd for 527.2757, Supplementary Figure S35). A detailed comparison of NMR spectroscopic data of **5** and hydroxymaltophilin (**11**, Figure 1) suggested that both compounds should have the same planar structure (Table 1 and Table 2, Supplementary Figures S36–S42) [27]. However, distinct from the *cis*-orientation of H-10/H-11 in **11** [27], a *trans*-orientation of H-10/H-11 was indicated in **5** by key NOE correlations of H-8/H-10, H10-/H-12, and H-11/H-29b (Figure 4, Supplementary Figures S41 and S42). Based on the similar ECD spectra of **5** and **11** (Supplementary Figure S10), **5** was suggested to have the configuration of 5*R*, 6*S*, 8*S*, 10*S*, 11*R*, 12*R*, 13*S*, 16*R*, 23*S*, and 25*S*, and it was thus designated 10-*epi*-hydroxymaltophilin.

Compound **6** was isolated as a yellowish solid. The molecular formula of **6** was determined to be C_29_H_38_N_2_O_6_ by HRESIMS ([M + H]^+^, *m*/*z* 511.2800, calcd for 511.2808, Supplementary Figure S43). An analysis of 1D, COSY, and HMBC correlations (Supplementary Figures S44–S48) showed that the planar structure of **6** was the same as that of FI-2 (**12**, Figure 1), an intermediate in frontalamide biosynthesis [15,27]. A *trans*-orientation of H-10/H-11 was indicated in **6** by key NOE correlations of H-8/H-10, H-10/H-12, and H-11/H-29b (Figure 4, Supplementary Figures S49 and S50), different from the *cis*-orientation of H-10/H-11 in **12** [27]. The high similarity in the ECD spectra of **6** and **12** indicated that **6** should be a 10-epimer of **12**, designated 10-*epi*-FI-2.

### 2.2. Biological Activities

The in vitro cytotoxicities of compounds **1**–**5** (compound **6** was not tested due to limited amount) were evaluated against four human cancer cell lines, including SF-268, MCF-7, A549, and HepG2, by the SRB method since most reported PTMs exhibits cytotoxic activities [1] (Table 3). Compounds **1**–**5** showed moderate activities against these four cancer cell lines with half inhibitory concentration (IC_50_) values of 2.47–17.68 μM, which were comparable to those of the positive control cisplatin (Table 3). It should be noted that pactamide A, differing from **2** only by lacking C-14 OH, displayed much better cytotoxicities (IC_50_ values ranging from 0.2–0.5 μM against these four cancer cell lines) than **2** [20].

### 2.3. Biosynthetic Implications

Based on bioinformatics analysis, the *ptm*′ BGC in *Streptomyces* sp. SCSIO 40010 was highly similar to that of frontalamides (*ftd*) in *Streptomyces* sp. SPB78 and it should be classified into the Group I of 5/5/6 type of PTM BGCs (Figure 2a). Subsequently, six new PTM analogues with moderate antitumor activities were isolated from *Streptomyces* sp. SCSIO 40010 and the absolute configuration at C-10 in these PTMs was identified as being 10*S*, opposite to their known PTM congeners. These observations further highlight the importance of *Streptomyces* species as prolific sources for bioactive compounds and they indicate the worth of genome mining in marine-derived Streptomycetes [35]. Similar to the well-established biosynthetic pathway for 5/5/6 type of PTMs [20,23,33], PtmA′ catalyzes the formation of a common polyene tetramate precursor, which is sequentially cyclized by PtmB2′/PtmB1′ into an intermediate with the 5/5 carbocyclic ring system (Figure 2b). It has been hypothesized that OX3, which is a PtmB2′ homologous enzyme, is involved in controlling the formation of products with both *cis*- and *trans*-orientated H-10/H-11 in HSAF (**7**) biosynthesis [23]. However, it appears that PtmB2′ only generates products with *trans*-orientated H-10/H-11. Additionally, it has been proposed that C-14 oxidation occurs during the OX2 (PtmB1′ analogue)-catalyzed formation of the second five-membered ring [23], and a recent in vivo combinatorial study has confirmed that the second ring formation is coupled with the C-14 hydroxylation in the biosynthesis of HSAF and analogues [33]. However, the detailed biochemistry and enzymology responsible for such transformations have not been elucidated. Next, PtmC’ generates the inner six-membered ring in **2** (Figure 2b). Finally, different oxidations of **2** by PtmD’ (a C-25 hydroxlase, analogous to FtdA for frontalamides [15], SD for HSAF [36]) and PtmE’ (a putative C-12 hydroxylase and C-14 dehydrogenase, analogous to FtdF for frontalamides [15]) lead to the formation of products **1** and **3**–**6** due to the substrate promiscuity of PtmD’ and PtmE’ (Figure 2b).

### 2.4. Conclusion

Conclusively, on the basis of a genome mining approach, we isolated six new PTMs **1**–**6** from the marine-derived *Streptomyces* sp. SCSIO 40010. The 10*S* absolute configuration is the unique feature of these new PTM analogues, which is distinct from the 10*R* configuration in their known congeners. PTMs **1**–**5** display moderate cytotoxic activities toward four human cancer cell lines. Although a biosynthetic pathway for PTMs **1**–**6** is proposed, the precise biochemistry and enzymology involved in the polycyclic ring formation and the stereochemistry selectivity remains elusive and awaits further investigations.

## 3. Materials and Methods

### 3.1. General Experimental Procedures

Optical rotations were measured using a 341 Polarimeter (Perkin-kinelmer, Inc., Norwalk, CT, USA). The CD spectra were measured on a Chirascan circular dichroism spectrometer (Applied Photophysics, Ltd., Surrey, UK). UV spectra were measured on a U-2900 spectrophotometer (Hitachi, Tokyo, Japan). IR spectra were recorded on an Affinity-1 FT-IR spectrometer (Shimadzu, Tokyo, Japan). The 1D and 2D NMR spectra were recorded on a Bruker AV-700 MHz NMR spectrometer (Bruker Biospin GmbH, Rheinstetten, Germany) with tetramethylsilane (TMS) as the internal standard. Mass spectrometric data were obtained on a quadrupole-time-of-flight mass spectrometry (Bruker Maxis 4G) for HRESIMS. Column chromatography was performed while using silica gel (100–200 mesh, 300–400 mesh; Jiangyou Silica gel development, Inc., Yantai, China), Sephadex LH-20 (GE Healthcare Bio-Sciences AB, Uppsala, Sweden). HPLC was carried out while using a reversed-phase column (Phenomenex Gemini C18, 250 mm × 4.6 mm, 5 μm; Phenomenex, Torrance, CA, USA) with UV detection at 270 nm and 320 nm. Semi-preparative HPLC was performed on a Hitachi HPLC station (Hitachi-L2130) with a Diode Array Detector (Hitachi L-2455) using a Phenomenex ODS column (250 mm × 10.0 mm, 5 mm; Phenomenex, Torrance, CA, USA) with UV detection at 320 nm.

### 3.2. Strain, Screening and Culture Methods

*Streptomyces* sp. SCSIO 40010 was isolated from the Mangrove sediment obtained from Penang, Malaysia, and it was identified by 16S rDNA sequence analysis. The strain SCSIO 40010 was maintained in 40% glycerol aqueous solution at −80 °C in Research Center for Marine Microbiology Culture Collection Center of South China Sea Institute of Oceanology, Chinese Academy of Sciences. It was found that the strain SCSIO 40010 was best maintained on 38#-Agar medium containing 3% sea salt for optimal growth and sporulation. A single colony was inoculated into 50 mL of four different media, including modifed-A1BFe+C (soluble starch 1.0%, yeast extract 0.4%, tryptone 0.2%, CaCO_3_ 0.2%, sea salts 3%, pH 7.2–7.4) [29], AM6 (soluble starch 2.0%, glucose 1.0%, tryptone 0.5%, yeast extract 0.5%, CaCO_3_ 0.2%, sea salts 3%, pH 7.2–7.4) [37], AM6-4 (glycerol 0.1%, bacterial peptone 0.5%, glycine 0.01%, alanine 0.01%, CaCO_3_ 0.5%, sea salts 3%, pH 7.2–7.4) [37], and modifed-ISP3 (oat meal 1.5%, FeSO_4_ 0.0001%, MnCl_2_ 0.0001%, ZnSO_4_ 0.0001%, sea salts 3%, pH 7.2–7.4) [37], in 250 mL Erlenmeyer flasks, and then incubated on a rotary shaker (200 rpm) at 28 °C for seven days. The culture broths were extracted with an equal volume of n-butanol and the extracts were then monitored by HPLC-DAD. HPLC analyses were carried out under the following program: solvent system (solvent A, 10% acetonitrile in water supplemented with 0.08% formic acid; solvent B, 90% acetonitrile in water); 5% B to 100% B (linear gradient, 0–18 min.), 100% B (18–23 min.), 100% B to 5% B (23–27 min.), 5% B (27–32 min.); flow rate at 1 mL/min. A single colony was inoculated into 30 mL of modifed-A1BFe+C medium and incubated at 28 °C for 2–3 days. Then, a total of 20 L fermentation cultures were performed by inoculating 30 mL of the seed culture into a 1000 mL Erlenmeyer flask containing 200 mL of the modifed-A1BFe+C medium to cultivate on a rotary shaker (200 rpm) at 28 °C for 7 days.

### 3.3. Genome Mining and Bioinformatics Analysis

The strain SCSIO 40010 was inoculated into modifed-A1BFe+C medium and incubated at 28 °C for 48 h. Then the mycelia were collected by centrifugation. Genomic DNA was released from the mycelia by lysozyme and proteinase K digestion, which was extracted with Phenol-chloroform, followed by anhydrous ethanol precipitation. The draft genome of *Streptomyces* sp. 40010 was sequenced by using Illumina HiSeq 2500. The reads were de novo assembled by using SOAPdenovo ver 2.04 (http://soap.genomics.org.cn/soapdenovo.html). Gene sequences were predicted and annotated by the Rapid Annotations using Subsystems Technology (RAST) server [38]. The putative PTM biosynthetic gene clusters in the genome were predicted with antiSMASH 4.0 [39]. The DNA sequences of the *ptm*′ gene cluster were deposited under GenBank accession number MN234160. The function of gene products was predicted with protein blast and/or blastx program (https://blast.ncbi.nlm.nih.gov/Blast.cgi). The PTM BGCs were obtained from GenBank database for bioinformatics analysis: *Streptomyces* sp. SCSIO 40010 (MN234160); *Streptomyces pactum* SCSIO 02999 (KU569222); *Streptomyces griseus* NBRC 13350 (AP009493); *Streptomyces albus* J1074 (ABYC01000481); *Streptomyces flavogriseus* ATCC 33331 (NZ_ACZH01000010); *Streptomyces roseosporus* ATCC 11379 (ABYX01000252); *Streptomyces* sp. SPB78 (NZ_ACEU01000453 and NZ_ACEU01000454); *Actinoalloteichus* sp. ADI127-7 (CP016076); *Actinoalloteichus* sp. AHMU CJ021 (CP025990.1); *Lysobacter enzymogenes* C3 (EF028635.2); *Lysobacter enzymogenes* strain YC36 (CP040656.1); *Lysobacter gummosus* strain 3.2.11 (CP011131.1); *Lysobacter capsici* strain 55 (CP011130.1).

### 3.4. Extraction, Isolation and Purification

The 20 L of culture broth of *Streptomyces* sp. SCSIO 40010 were pooled and centrifuged at 3900 rpm for 15 min. at 25 °C. The mycelia were extracted three times, each with 2 L acetone. The acetone extracts were concentrated under reduced pressure to afford an aqueous residue, which was extracted four times with equal volume of *n*-butanone. The supernatants were extracted four times with equal volume of *n*-butanone. The butanone extracts were combined and concentrated under reduced pressure to afford the crude extracts (11.5 g). The crude extracts were subjected to the column chromatography over silica gel eluting with a gradient of CHCl_3_/MeOH mixtures ranging from 100/0, 95/5, 90/10, 80/20,50/50 and 0/100 (*v*/*v*) yielded six fractions (Fr.1–Fr.6). Then Fr.2 (0.72 g) was further purified via MPLC (Medium Pressure Preparative Liquid Chromatography) with reverse phased C-18 column (14.5 × 2.5 cm i.d., 5 mm Agela Technologies) by eluting with a linear gradient of H_2_O/MeOH (0–100%, 15 mL/min, 300 min) give fractions Fr.2.1–Fr.2.18. Fractions Fr.2.14–15 (170 mg) were further purified by semi-preparative HPLC while using a mobile phase of MeCN-H_2_O (65:35, *v*/*v*) to give compounds **2** (3.4 mg), **3** (10.8 mg), and **4** (3.6 mg). The fraction Fr.3 (0.83 g) was purified by Sephadex LH-20 (120 × 3.5 cm i.d.), eluting with CHCl_3_/MeOH (5:5, *v*/*v*) to give fractions Fr.3.1–Fr.3.25. Fractions Fr.3.5–9 (300 mg) were further purified by semi-preparative HPLC while using a mobile phase of MeCN-H_2_O (45:55, *v*/*v*) to provide compounds **1** (4.1 mg), **5** (5.6 mg), and **6** (2.8 mg).

### 3.5. Physical and Chemical Properties of New Compounds 1–6

10-*epi*-HSAF (**1**): White powder; [α${]}_{D}^{25}$ + 50.7 (*c* 0.2, MeOH); UV (MeOH) λ_max_ (log ε) 322 (3.92) nm, 219 (4.18) nm; ECD (*c* 4.3 × 10^−4^ M, MeOH) λ_max_ (Δε) 215 (+15.5), 241 (−18.1), 326 (+6.2) nm; IR *ν*_max_ 3356, 2951, 2918, 2369, 2341, 1653, 1541, 1471, 1020, 679 cm^−1^; ^1^H NMR (700 MHz, DMSO-*d*_6_) and ^13^C NMR (176 MHz, DMSO-*d*_6_) data, see Table 1 and Table 2; (+)-HRESIMS *m*/*z* [M + H]^+^ 513.2960 (calcd for C_29_H_41_N_2_O_6_, 513.2965).

10-*epi*-deOH-HSAF (**2**): White powder; [α${]}_{D}^{25}$ + 53.7 (*c* 0.2, MeOH); UV (MeOH) λ_max_ (log ε) 322 (4.03) nm, 212 (4.37) nm; ECD (*c* 2.2 × 10^−4^ M, MeOH) λ_max_ (Δε) 214 (+7.8), 244 (−9.9), 326 (+4.4) nm; IR *ν*_max_ 3356, 3334, 2953, 2868, 2358, 2341, 1647, 1541, 1506, 1203, 1024, 669 cm^−1^; ^1^H NMR (700 MHz, DMSO-*d*_6_) and ^13^C NMR (176 MHz, DMSO-*d*_6_) data, see Table 1 and Table 2; (+)-HRESIMS *m/z* [M + H]^+^ 497.3010 (calcd for C_29_H_41_N_2_O_5_, 497.3015).

10-*epi*-maltophilin (**3**): Reddish solid; [α${]}_{D}^{25}$ + 42.4 (*c* 0.06, MeOH); UV (MeOH) λ_max_ (log ε) 322 (4.06) nm, 218 (4.31) nm; ECD (*c* 4.9 × 10^−4^ M, MeOH) λ_max_ (Δε) 214 (+26.3), 238 (−23.1), 332 (+6.0) nm; IR *ν*_max_ 3336, 2953, 2920, 2358, 2341, 1647, 1456, 1022, 679 cm^−1^; ^1^H NMR (700 MHz, DMSO-*d*_6_) and ^13^C NMR (176 MHz, DMSO-*d*_6_) data, see Table 1 and Table 2; (-)-HRESIMS *m/z* [M − H]^−^ 509.2642 (calcd for C_29_H_37_N_2_O_6_, 509.2952).

10-*epi*-xanthobaccin C (**4**): White powder; [α${]}_{D}^{25}$ + 8.31 (*c* 0.08, MeOH); UV (MeOH) λ_max_ (log ε) 322 (3.97) nm, 219 (4.27) nm; ECD (*c* 2.6 × 10^−4^ M, MeOH) λ_max_ (Δε) 210 (+15.3), 247 (−16.9), 327 (+5.0) nm; IR *ν*_max_ 3335, 2951, 2920, 2837, 2358, 2341, 1653, 1456, 1018, 758, 669 cm^−1^; ^1^H NMR (700 MHz, DMSO-*d*_6_) and ^13^C NMR (176 MHz, DMSO-*d*_6_) data, see Table 1 and Table 2; (+)-HRESIMS *m/z* [M + H]^+^ 495.2846 (calcd for C_29_H_39_N_2_O_5_, 495.2859).

10-*epi*-hydroxymaltophilin (**5**): Reddish powder; [α${]}_{D}^{25}$ + 30.8 (*c* 0.06, MeOH); UV (MeOH) λ_max_ (log ε) 321 (4.02) nm, 216 (4.32) nm; ECD (*c* 4.0 × 10^−4^ M, MeOH) λ_max_ (Δε) 214 (+24.1), 238 (−18.4), 326 (+4.4) nm; IR *ν*_max_ 3334, 3327, 2955, 2927, 2359, 2342, 1697, 1653, 1541, 1471, 1217, 1024, 754, 678 cm^−1^; ^1^H NMR (700 MHz, DMSO-*d*_6_) and ^13^C NMR (176 MHz, DMSO-*d*_6_) data, see Table 1 and Table 2; (+)-HRESIMS *m/z* [M + H]^+^ 527.2757 (calcd for C_29_H_39_N_2_O_7_, 527.2757).

10-*epi*-FI-2 (**6**): Yellowish solid; [α${]}_{D}^{25}$ + 41.4 (*c* 0.06, MeOH); UV (MeOH) λ_max_ (log ε) 322 (3.96) nm, 226 (4.39) nm; ECD (*c* 2.9 × 10^−4^ M, MeOH) λ_max_ (Δε) 209 (+12.0), 239 (−7.4), 332 (+2.7) nm; IR *ν*_max_ 3321, 2957, 2926, 2359, 2342, 1684, 1647, 1541, 1456, 1238, 669 cm^−1^; ^1^H NMR (700 MHz, DMSO-*d*_6_) and ^13^C NMR (176 MHz, DMSO-*d*_6_) data, see Table 1 and Table 2; (+)-HRESIMS *m/z* [M + H]^+^ 511.2800 (calcd for C_29_H_39_N_2_O_6_, 511.2808).

### 3.6. Bioactivity Assays

The in vitro cytotoxic activities of PTMs **1**–**5** were evaluated against four tumor cell lines, SF-268 (human glioma cell line), HepG2 (human liver carcinoma cell line), and MCF-7 (human breast adenocarcinoma cell line), A549 (human lung adenocarcinoma cell) by the SRB method, according to a previously described protocol [40]. All of the cells were cultivated in RPMI 1640 medium [41]. Cells (180 μL) with a density of 3 × 10^4^ cells/mL were seeded onto 96-well plates and incubated for 24 h at 37 °C, 5% CO_2_. Subsequently, 20 μL of different concentrations of PTM compounds, ranging from 0 to 100 µM in dimethyl sulfoxide (DMSO), were added to each plate well. Equal volume of DMSO was used as a negative control. After a further incubation for 72 h, the cell monolayers were fixed with 50% (*wt*/*v*) trichloroacetic acid (50 μL) and then stained for 30 min. with 0.4% (*wt*/*v*) SRB dissolved in 1% acetic acid. Unbound dye was removed by repeatedly washing with 1% acetic acid. The protein-bound dye was dissolved in 10 mM Tris-base solution (200 µL) for the determination of optical density (OD) at 570 nm while using a microplate reader. The cytotoxic compound cisplatin was used as a positive control. All of the data were obtained in triplicate and presented as means ± S.D. IC_50_ values were calculated with the SigmaPlot 14.0 software using the non-linear curve-fitting method.

## Figures and Tables

**Table 1:** ^1^H NMR (700 MHz) Data for PTMs **1**–**6** in dimethyl sulfoxide (DMSO)-*d*_6_ (*δ*_H_, mult, *J* in Hz).

No.	1	2	3	4	5	6
2	5.70, dd, (2.3, 11.5)	5.73, dd, (1.9, 11.4)	5.72, dd, (2.2, 11.3)	5.75, dd, (2.0, 11.5)	5.75, dd, (2.1, 11.3)	5.77, dd, (2.0, 11.5)
3	5.90, td, (1.8, 11.2)	5.9, td, (2.2, 11.1)	5.92, td, (2.1, 11.0)	5.92, td, (2.1, 11.1)	5.96, td, (2.1, 11.1)	5.96, td, (2.2, 11.1)
4a	1.89, m	1.92, m	2.00, m	2.04, m	2.03, m	2.05, m
4b	3.52, m	3.49, m	3.62, m	3.62, m	3.65, m	3.64, m
5	1.28, m	1.27, m	1.90, m	1.90, m	1.87, m	1.88, m
6	1.64, m	1.27, m	2.09, m	2.10, m	2.56, m	2.57, m
7a	0.86, m	0.87, m	1.05, m	0.85, m	0.98, m	1.01, m
7b	1.96, m	1.97, m	2.05, m	2.06, m	2.11, m	2.14, m
8	2.35, m	2.35, m	2.35, m	2.35, m	2.03, m	2.03, m
9a	0.80, m	0.79, m	0.84, m	1.06, m	0.68, m	0.69, m
9b	2.01, m	2.01, m	2.05, m	2.06, m	2.02, m	2.03, m
10	1.33, m	1.32, m	1.37, m	1.37, m	1.53, m	1.53, m
11	1.27, m	1.64, m	1.09, m	1.09, m	1.21, m	1.22, m
12	1.75, m	1.77, m	2.21, m	2.21, m		
13	1.10, m	1.09, m	2.37, m	2.37, m	2.32, m	2.32, m
14	3.25, m	3.25, m				
15a	1.24, m	1.24, m	2.11, m	2.11, m	2.12, m	2.13, m
15b	1.74, m	1.75, m	2.59, m	2.59, m	2.59, m	2.60, m
16	2.06, m	2.07, m	2.38, m	2.40, m	2.44, m	2.47, m
17	6.57, dd, (10.5, 15.5)	6.55, dd, (10.5, 15.5)	6.63, dd, (10.3, 15.6)	6.61, t, (15.5)	6.60, dd, (10.3, 15.5)	6.59, dd, (11.5, 15.5)
18	6.86, d, (15.5)	6.95, d, (15.5)	6.87, d, (15.6)	6.96, d, (15.5)	6.88, d, (15.5)	6.96, d, (15.5)
22	NH, 8.68, s	NH, 8.68, s	NH, 8.95, s	NH, 8.73, brs	NH, 8.96, s	NH, 8.75, s
23	3.86, d, (1.2)	3.81, d, (5.7)	3.87, d, (1.4)	3.83, s	3.87, d, (1.1)	3.84, d, (6.1)
25a	3.81, dt, (1.5, 6.1)	1.74, m	3.81, dt, (1.4,6.2)	1.74, m	3.82, dt, (2.0, 6.4)	1.74, m
25b		1.84, m		1.86, m		1.86, m
26a	1.18, m	1.15, m	1.18, m	1.17, m	1.20, m	1.15, m
26b	1.38, m	1.32, m	1.39, m	1.35, m	1.39, m	1.34, m
27a	2.57, m	2.39, m	2.59, m	3.23, m	2.58 m	2.40, m
27b	3.25, m	3.22, m	3.26, m	2.39, m	3.24, m	3.23, m
28	NH, 7.96, t, (5.7)	NH, 7.82, t, (5.3)	NH, 7.98, t, (5.6)	NH, 7.86, s	NH, 8.00, t, (5.6)	NH, 7.88, t, (5.6)
29a	1.04, m	1.04, m	1.03, m	1.03, m	0.99, m	1.02, m
29b	1.55, m	1.55, m	1.53, m	1.54, m	1.51, m	1.52, m
30	0.85, t (7.4)	0.85, t (7.4)	0.84, t (7.4)	0.84, t (7.4)	0.84, t (7.4)	0.84, t (7.4)
31	1.06, d (6.4)	1.06, d (6.4)	0.96, d (6.5)	0.96, d (6.5)	0.94, d (6.7)	0.94, d (6.8)

**Table 2:** ^13^C NMR (176 MHz) Data for PTMs **1**–**6** in DMSO-*d*_6_, (*δ*_C_, type).

No.	1	2	3	4	5	6
1	165.5, C	165.5, C	165.5, C	165.6, C	165.5, C	165.6, C
2	124.1, CH	124.2, CH	124.4, CH	124.5, CH	124.5, CH	124.7, CH
3	139.1, CH	138.9, CH	138.4, CH	138.3, CH	138.2, CH	138.1, CH
4	28.0, CH_2_	28.1, CH_2_	27.7, CH_2_	27.7, CH_2_	27.4, CH_2_	27.4, CH_2_
5	43.5, CH	43.5, CH	43.2, CH	43.2, CH	43.0, CH	43.0, CH
6	47.4, CH	46.4, CH	51.1, CH	51.2, CH	47.9, CH	48.0, CH
7	37.2, CH_2_	37.3, CH_2_	38.4, CH_2_	39.7, CH_2_	36.5, CH_2_	36.5, CH_2_
8	41.5, CH	41.4, CH	40.4, CH	40.4, CH	51.3, CH	51.3, CH
9	40.3, CH_2_	40.3, CH_2_	39.6, CH_2_	38.5, CH_2_	38.1, CH_2_	38.1, CH_2_
10	53.5, CH	53.5, CH	53,2, CH	53,2, CH	50.4, CH	50.4, CH
11	46.5, CH	47.6, CH	46.7, CH	46.7, CH	49.3, CH	49.3, CH
12	58.1, CH	58.1, CH	50.4, CH	50.4, CH	89.7, CH	89.7, CH
13	59.1, CH	59.1, CH	63.0, CH	63.0, CH	64.2, CH	64.2, CH
14	72.7, CH	72.7, CH	207.4, C	207.4, C	210.1 C	210.2, C
15	41.9, CH_2_	41.9, CH_2_	45.6, CH_2_	45.6, CH	46.0, CH_2_	46.0, CH_2_
16	45.7, CH	45.6, CH	47.7, CH	47.8, CH	47.1, CH	47.2, CH
17	150.1, CH	149.6, CH	147.8, CH	147.4, CH	147.5, CH	147.2, CH
18	121.3, CH	121.5, CH	122.0, CH	122.1, CH	122.1, CH	122.2, CH
19	172.2, C	171.8, C	171.9, C	175.1, C	171.8, C	171.2, C
20	100.4, C	100.7, C	100.7, C	101.1, C	100.7, C	101.0, C
21	175.7, C	175.3, C	175.6, C	171.4, C	175.6, C	175.1, C
23	68.6, CH	61.0, CH	68.5, CH	61.1, CH	68.6, CH	61.1, CH
24	193.0, C	195.8, C	193.0, C	195.8, C	193.0, C	195.9, C
25	70.1, CH	26.2, CH_2_	70.1, CH	26.2, CH_2_	70.1, CH	26.1, CH_2_
26	31.1, CH_2_	20.4, CH_2_	31.0, CH_2_	20.4, CH_2_	31.0, CH_2_	31.1, CH_2_
27	36.4, CH_2_	38.0, CH_2_	36.4, CH_2_	38.0, CH_2_	36.4, CH_2_	36.4, CH_2_
29	25.8, CH_2_	25.8, CH_2_	25.5, CH_2_	25.5, CH_2_	25.3, CH_2_	25.3, CH_2_
30	12.6, CH_3_	12.6, CH_3_	12.4, CH_3_	12.4, CH_3_	12.0, CH_3_	12.0, CH_3_
31	18.4, CH_3_	18.4, CH_3_	17.6, CH_3_	17.6, CH_3_	11.5, CH_3_	11.5, CH_3_

**Table 3:** Cytotoxicities of PTMs **1**–**5**.

	IC_50_ (μM)			
	SF-268	MCF-7	A549	HepG2
**1**	3.83 ± 0.13	2.47 ± 0.05	5.99 ± 0.15	3.48 ± 0.17
**2**	10.62 ± 0.45	3.84 ± 0.07	11.01 ± 1.09	10.34 ± 0.88
**3**	4.57 ± 0.18	3.18 ± 0.13	3.75 ± 0.62	6.30 ± 0.34
**4**	7.53 ± 0.27	3.54 ± 0.24	10.45 ± 0.46	17.86 ± 0.62
**5**	3.21 ± 0.18	6.83 ± 0.36	3.28 ± 0.04	3.12 ± 0.11
***^a^* CP**	3.26 ± 0.29	3.19 ± 0.12	1.56 ± 0.08	2.42 ± 0.14

*^a^* Cisplatin, positive control.

## Supplementary Materials

The following are available online at https://www.mdpi.com/1660-3397/17/12/663/s1, Figure S1: HPLC analysis of metabolite profiles of *Streptomyces* sp: SCSIO 40010 cultured in different media; Figure S2: Comparison of ECD spectra of compound **1**–**6** and the known compounds; Figure S3: HRESIMS (**a**) and IR (**b**) of compound **1**; Figure S4: ^1^H NMR spectrum of compound **1** in DMSO-*d*_6_; Figure S5: The ^13^C NMR and DEPT 135 spectra of compound **1** in DMSO-*d*_6_; Figure S6: The HSQC spectrum of compound **1** in DMSO-*d*_6_; Figure S7: The HMBC spectrum of compound **1** in DMSO-*d*_6_; Figure S8: The ^1^H-^1^H COSY spectrum of compound **1** in DMSO-*d*_6_; Figure S9: The NOESY spectrum of compound **1** in DMSO-*d*_6_; Figure S10: The key NOESY spectrum of compound **1** in DMSO-*d*_6_; Figure S11: HRESIMS (**a**) and IR (**b**) of compound **2**; Figure S12: ^1^H NMR spectrum of compound **2** in DMSO-*d*_6_; Figure S13: The ^13^C NMR and DEPT 135 spectra of compound **2** in DMSO-*d*_6_; Figure S14: The HSQC spectrum of compound **2** in DMSO-*d*_6_; Figure S15: The HMBC spectrum of compound **2** in DMSO-*d*_6_; Figure S16: The ^1^H-^1^H COSY spectrum of compound **2** in DMSO-*d*_6_; Figure S17: The NOESY spectrum of compound **2** in DMSO-*d*_6_; Figure S18: The key NOESY spectrum of compound **2** in DMSO-*d*_6_, Figure S19: HRESIMS (**a**) and IR (**b**) of compound **3**; Figure S20: ^1^H NMR spectrum of compound **3** in DMSO-*d*_6_; Figure S21: The ^13^C NMR and DEPT 135 spectra of compound **3** in DMSO-*d*_6_; Figure S22: The HSQC spectrum of compound **3** in DMSO-*d*_6_; Figure S23: The HMBC spectrum of compound **3** in DMSO-*d*_6_; Figure S24: The ^1^H-^1^HCOSY spectrum of compound **3** in DMSO-*d*_6_; Figure S25: The NOESY spectrum of compound **3** in DMSO-*d*_6_; Figure S26: The key NOESY spectrum of compound **3** in DMSO-*d*_6_; Figure S27: HRESIMS (**a**) and IR (**b**) of compound **4**; Figure S28: ^1^H NMR spectrum of compound **4** in DMSO-*d*_6_; Figure S29: The ^13^C NMR and DEPT 135 spectra of compound **4** in DMSO-*d*_6_; Figure S30: The HSQC spectrum of compound **4** in DMSO-*d*_6_; Figure S31: The HMBC spectrum of compound **4** in DMSO-*d*_6_; Figure S32: The ^1^H-^1^HCOSY spectrum of compound **4** in DMSO-*d*_6_; Figure S33: The NOESY spectrum of compound **4** in DMSO-*d*_6_; Figure S34: The key NOESY spectrum of compound **4** in DMSO-*d*_6_; Figure S35: HRESIMS (**a**) and IR (**b**) of compound **5**; Figure S36: ^1^H NMR spectrum of compound **5** in DMSO-*d*_6_; Figure S37: The ^13^C NMR and DEPT 135 spectra of compound **5** in DMSO-*d*_6_; Figure S38: The HSQC spectrum of compound **5** in DMSO-*d*_6_; Figure S39: The HMBC spectrum of compound **5** in DMSO-*d*_6_; Figure S40: The ^1^H-^1^HCOSY spectrum of compound **5** in DMSO-*d*_6_; Figure S41: The NOESY spectrum of compound **5** in DMSO-*d*_6_; Figure S42: The key NOESY spectrum of compound **5** in DMSO-*d*_6_; Figure S43: HRESIMS (**a**) and IR (**b**) of compound **6**; Figure S44: ^1^H NMR spectrum of compound **6** in DMSO-*d*_6_; Figure S45: The ^13^C NMR and DEPT 135 spectra of compound **6** in DMSO-*d*_6_; Figure S46: The HSQC spectrum of compound **6** in DMSO-*d*_6_; Figure S47: The HMBC spectrum of compound **6** in DMSO-*d*_6_; Figure S48: The ^1^H-^1^H COSY spectrum of compound **6** in DMSO-*d*_6_; Figure S49: The NOESY spectrum of compound **6** in DMSO-*d*_6_; Figure S50: The key NOESY spectrum of compound **6** in DMSO-*d*_6_.
