# Supplementary material for: Genome Mining of Marine-Derived Streptomyces sp. SCSIO 40010 Leads to Cytotoxic New Polycyclic Tetramate Macrolactams
Source: Mar Drugs. 2019 Nov 25;17(12):663. doi: 10.3390/md17120663 (PMC6950151; doi:10.3390/md17120663)

Supplementary Materials

Genome Mining of Marine-derived *Streptomyces* sp. SCSIO 40010 Leads to New Polycyclic Tetramate Macrolactams

Wei Liu [1,2], Wenjun Zhang [2], Hongbo Jin [2], Qingbo Zhang [2], Yuchan Chen [3], Xiaodong Jiang [2], Guangtao Zhang [2], Liping Zhang [2], Weimin Zhang [3], Zhigang She [1], Changsheng Zhang [2*]

1 South China Sea Resource Exploitation and Protection Collaborative Innovation Center (SCS-REPIC)/School of Marine Sciences, Sun Yat-sen University, Guangzhou 510006, China; 448242066@qq.com (W.L.); cesshzhg@mail.sysu.edu.cn (Z.S.);

2 CAS Key Laboratory of Tropical Marine Bio-resources and Ecology, Guangdong Key Laboratory of Marine Materia Medica, South China Sea Institute of Oceanology, Innovation Academy for South China Sea Ecology and Environmental Engineering, Chinese Academy of Sciences, 164 West Xingang Road, Guangzhou 510301, China; wzhang@scsio.ac.cn (W.Z.); khbmail@126.com (H.J.); zhanglp@scsio.ac.cn (L.Z.); gtzhang@scsio.ac.cn (G.Z.); gudaobo@163.com (Q.Z.); czhang2006@gmail.com (C.Z.)

3 State Key Laboratory of Applied Microbiology Southern China, Guangdong Institute of Microbiology, 100 Central Xianlie Road, Guangzhou 510070, China; chenyc@gdim.cn (Y.C.); wmzhang@gdim.cn (W.Z.)

* Correspondence: czhang2006@gmail.com; Tel.: +86-20-8902-3038 (C.Z.)

Content

Figure S1. HPLC analysis of metabolite profiles of *Streptomyces* sp. SCSIO 40010 cultured in different media........S4

Figure S2. HRESIMS (**a**) and IR(**b**) of compound **1** S5

Figure S3. ^{1}H NMR spectrum of compound **1** in DMSO-d_6........S6

Figure S4. The ^{13}C NMR and DEPT 135 spectra of compound **1** in DMSO-d_6........S7

Figure S5. The HSQC spectrum of compound **1** in DMSO-d_6 ....S8

Figure S6. The HMBC spectrum of compound **1** in DMSO-d_6........S9

Figure S7. The ^{1}H-^{1}H COSY spectrum of compound **1** in DMSO-d_6........S10

Figure S8. The NOESY spectrum of compound **1** in DMSO-d_6 ....S11

Figure S9. The key NOESY spectrum of compound **1** in DMSO-d_6 ....S12

Figure S10. Comparison of ECD spectra of compound **1-6** and the known compounds........S13

Figure S11. HRESIMS (**a**) and IR(**b**) of compound **2** S14

Figure S12. ^{1}H NMR spectrum of compound **2** in DMSO-d_6........S15

Figure S13. The ^{13}C NMR and DEPT 135 spectra of compound **2** in DMSO-d_6........S16

Figure S14. The HSQC spectrum of compound **2** in DMSO-d_6........S17

Figure S15. The HMBC spectrum of compound **2** in DMSO-d_6........S18

Figure S16. The ^{1}H-^{1}H COSY spectrum of compound **2** in DMSO-d_6........S19

Figure S17. The NOESY spectrum of compound **2** in DMSO-d_6 ....S20

Figure S18. The key NOESY spectrum of compound **2** in DMSO-d_6 ....S21

Figure S19. HRESIMS (**a**) and IR(**b**) of compound **3** S22

Figure S20. ^{1}H NMR spectrum of compound **3** in DMSO-d_6........S23

Figure S21. The ^{13}C NMR and DEPT 135 spectra of compound **3** in DMSO-d_6........S24

Figure S22. The HSQC spectrum of compound **3** in DMSO-d_6........S25

Figure S23. The HMBC spectrum of compound **3** in DMSO-d_6........S26

Figure S24. The ^{1}H-^{1}HCOSY spectrum of compound **3** in DMSO-d_6........S27

Figure S25. The NOESY spectrum of compound **3** in DMSO-d_6 ....S28

Figure S26. The key NOESY spectrum of compound **3** in DMSO-d_6 ....S29

Figure S27. HRESIMS (**a**) and IR(**b**) of compound **4** S30

Figure S28. ^{1}H NMR spectrum of compound **4** in DMSO-d_6........S31

Figure S29. The ^{13}C NMR and DEPT 135 spectra of compound **4** in DMSO-d_6........S32

Figure S30. The HSQC spectrum of compound **4** in DMSO-d_6........S33

Figure S31. The HMBC spectrum of compound **4** in DMSO-d_6........S34

Figure S32. The ^{1}H-^{1}HCOSY spectrum of compound **4** in DMSO-d_6 .... S35

Figure S33. The NOESY spectrum of compound **4** in DMSO-d_6 .... S36

Figure S34. The key NOESY spectrum of compound **4** in DMSO-d_6 .... S37

Figure S35. HRESIMS (**a**) and IR(**b**) of compound **5** S38

Figure S36. ^{1}H NMR spectrum of compound **5** in DMSO-d_6. .... S39

Figure S37. The ^{13}C NMR and DEPT 135 spectra of compound **5** in DMSO-d_6. .... S40

Figure S38. The HSQC spectrum of compound **5** in DMSO-d_6.. S41

Figure S39. The HMBC spectrum of compound **5** in DMSO-d_6.. S42

Figure S40. The ^{1}H-^{1}HCOSY spectrum of compound **5** in DMSO-d_6 .... S43

Figure S41. The NOESY spectrum of compound **5** in DMSO-d_6 .... S44

Figure S42. The key NOESY spectrum of compound **5** in DMSO-d_6 .... S45

Figure S43. HRESIMS (**a**) and IR(**b**) of compound **6** S46

Figure S44. ^{1}H NMR spectrum of compound **6** in DMSO-d_6. .... S47

Figure S45. The ^{13}C NMR and DEPT 135 spectra of compound **6**in DMSO-d_6. .... S48

Figure S46. The HSQC spectrum of compound **6** in DMSO-d_6.. S49

Figure S47. The HMBC spectrum of compound **6** in DMSO-d_6.. S50

Figure S48. The ^{1}H-^{1}HCOSY spectrum of compound **6** in DMSO-d_6 .... S51

Figure S49. The NOESY spectrum of compound **6** in DMSO-d_6 .... S52

Figure S50. The key NOESY spectrum of compound **6** in DMSO-d_6 .... S53

Figure S1. HPLC analysis of metabolite profiles of *Streptomyces* sp. SCSIO 40010 cultured in different media.

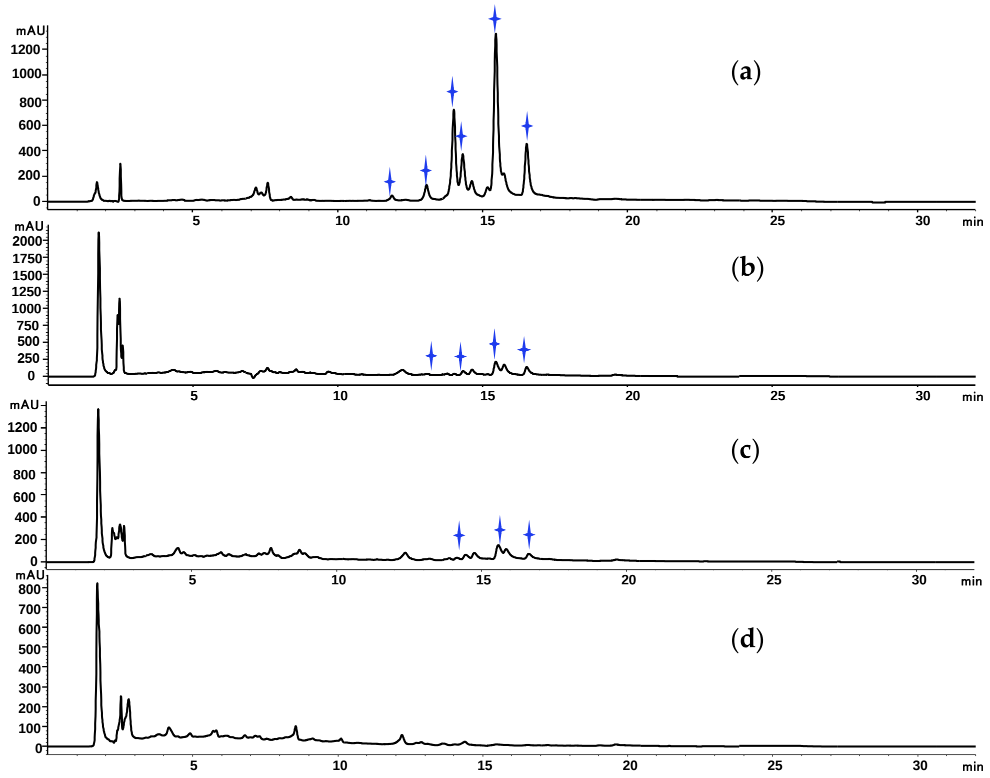

Note: bule star-PTM analogue.(**a**) modifed-A1BFe+C (soluble starch 1.0%, yeast extract 0.4%, tryptone 0.2%, $CaCO_3$ 0.2%, sea salts 3%, pH 7.2–7.4); (**b**) AM6 medium (soluble starch 2.0%, glucose 1.0%, tryptone 0.5%, yeast extract 0.5%, $CaCO_3$ 0.2%, sea salts 3%, pH 7.2–7.4); (**c**) AM6-4 (glycerol 0.1%, bacterial peptone 0.5%, glycine 0.01%, alanine 0.01%, $CaCO_3$ 0.5%, sea salts 3%, pH 7.2-7.4). (**d**) Modified-ISP3 (oat meal 1.5%, $FeSO_4$ 0.0001%, MnCl2 0.0001%, $ZnSO_4$ 0.0001%, sea salts 3%, pH 7.2–7.4).

Figure S2. HRESIMS (**a**) and IR(**b**) of compound **1**.

(a) **HRESIMS**

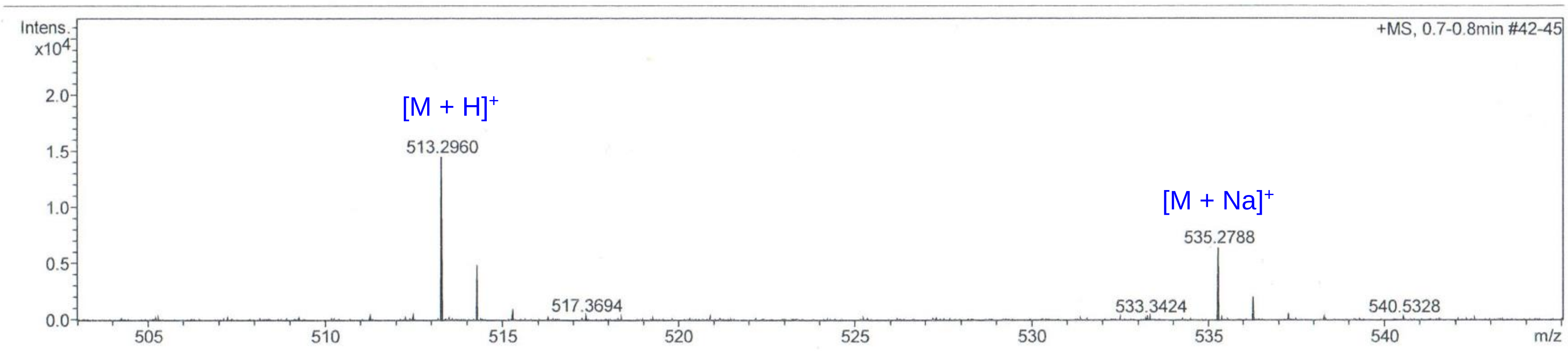

(**b**) **IR**

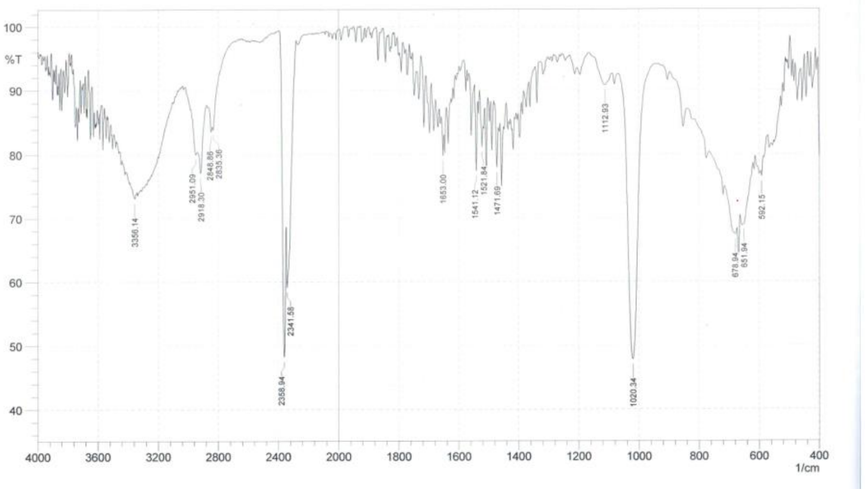

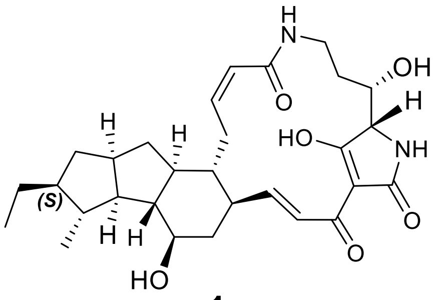

1

Chemical Formula: $C_{29}H_{40}N_2O_6$

caculated for $[M+H]^+$: 513.2965

caculated for $[M+Na]^+$: 535.2784

Figure S3. ^{1}H NMR spectrum of compound **1** in DMSO-d_6.

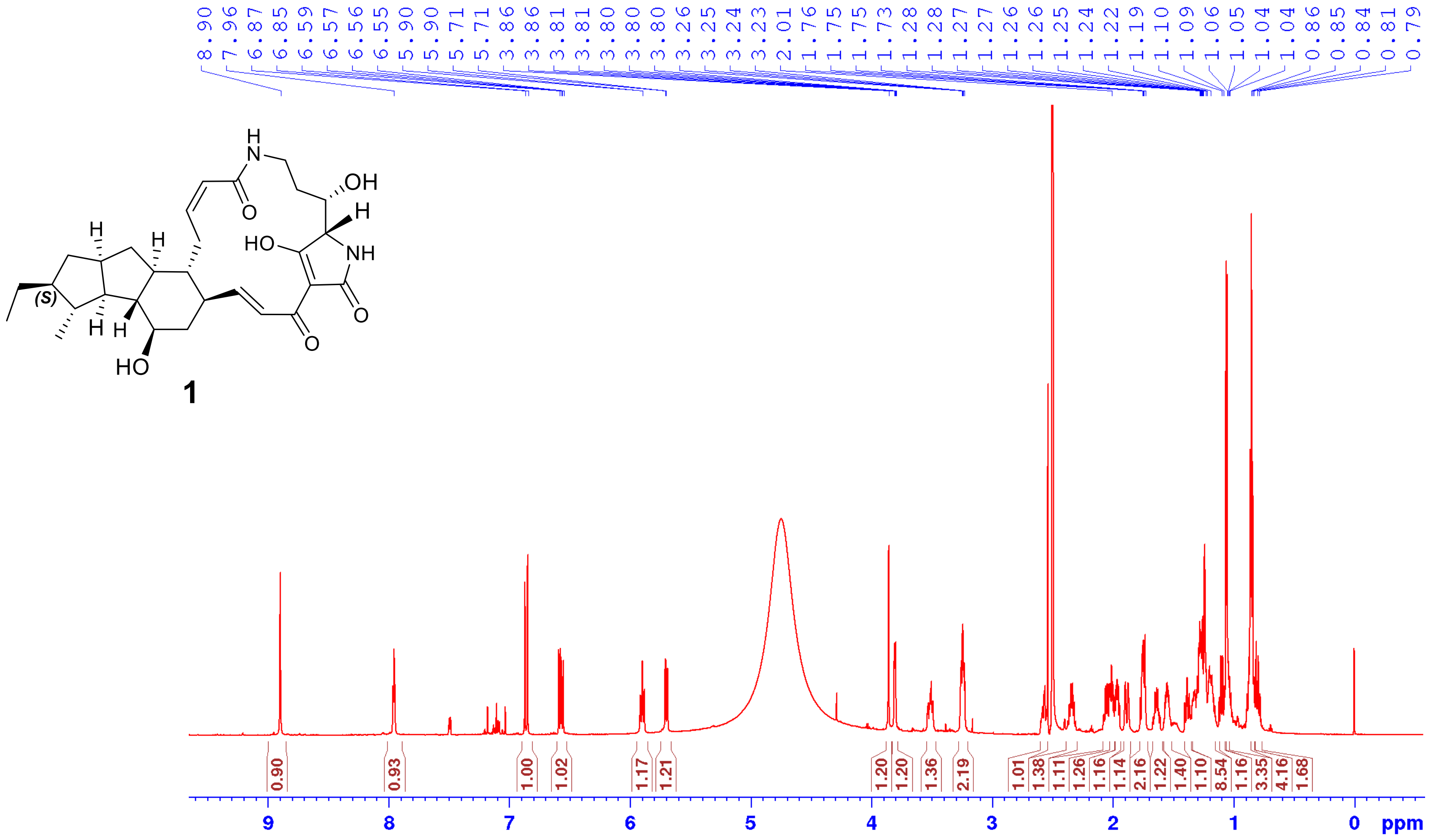

Figure S4. The ^{13}C NMR and DEPT 135 spectra of compound **1** in DMSO-d_6.

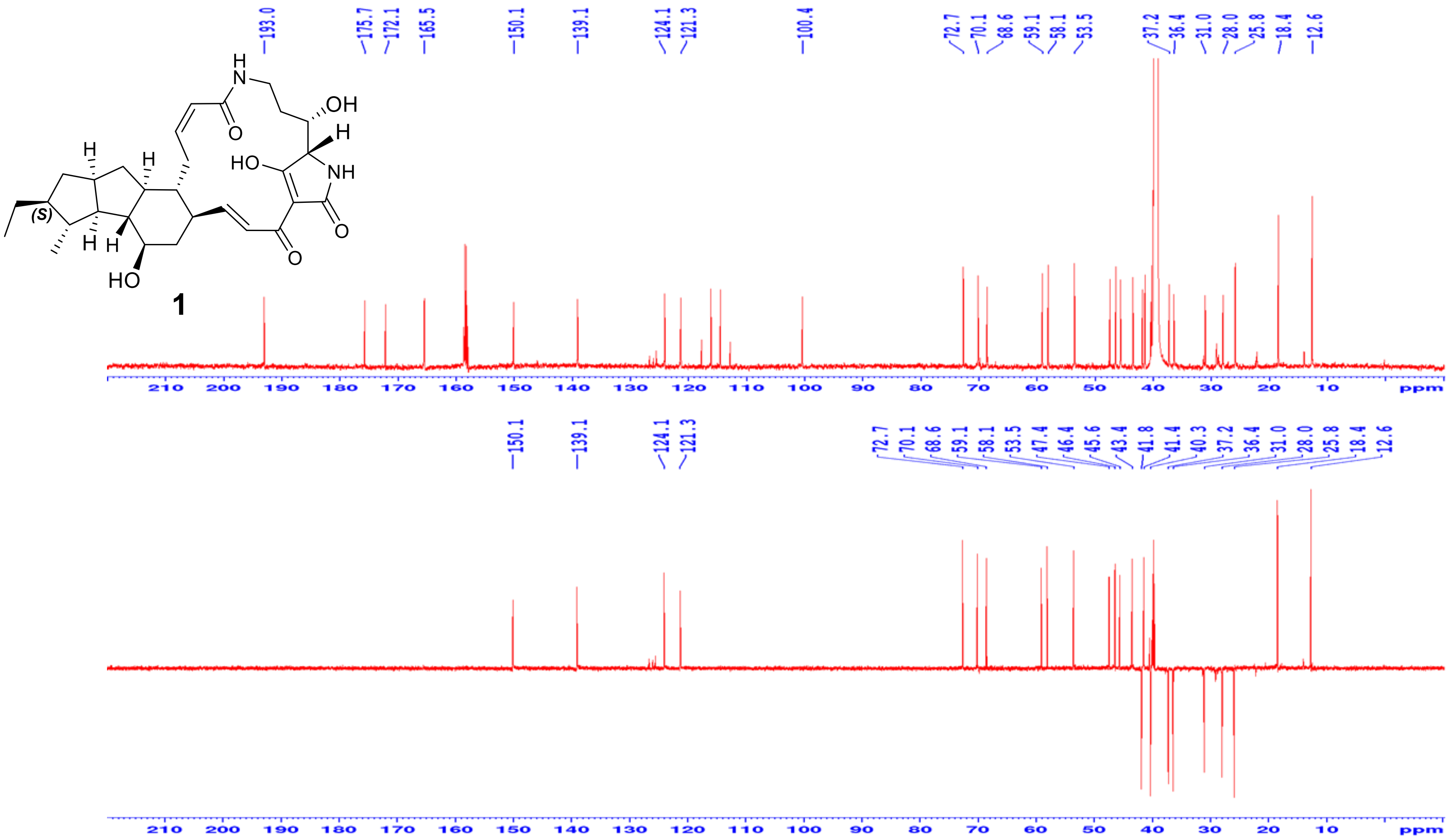

Figure S5. The HSQC spectrum of compound **1** in DMSO-d_6.

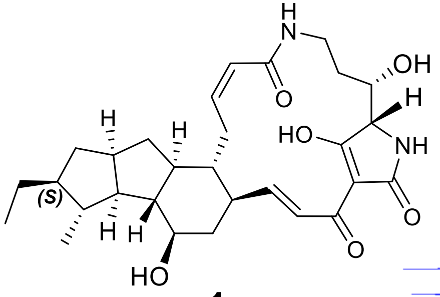

1

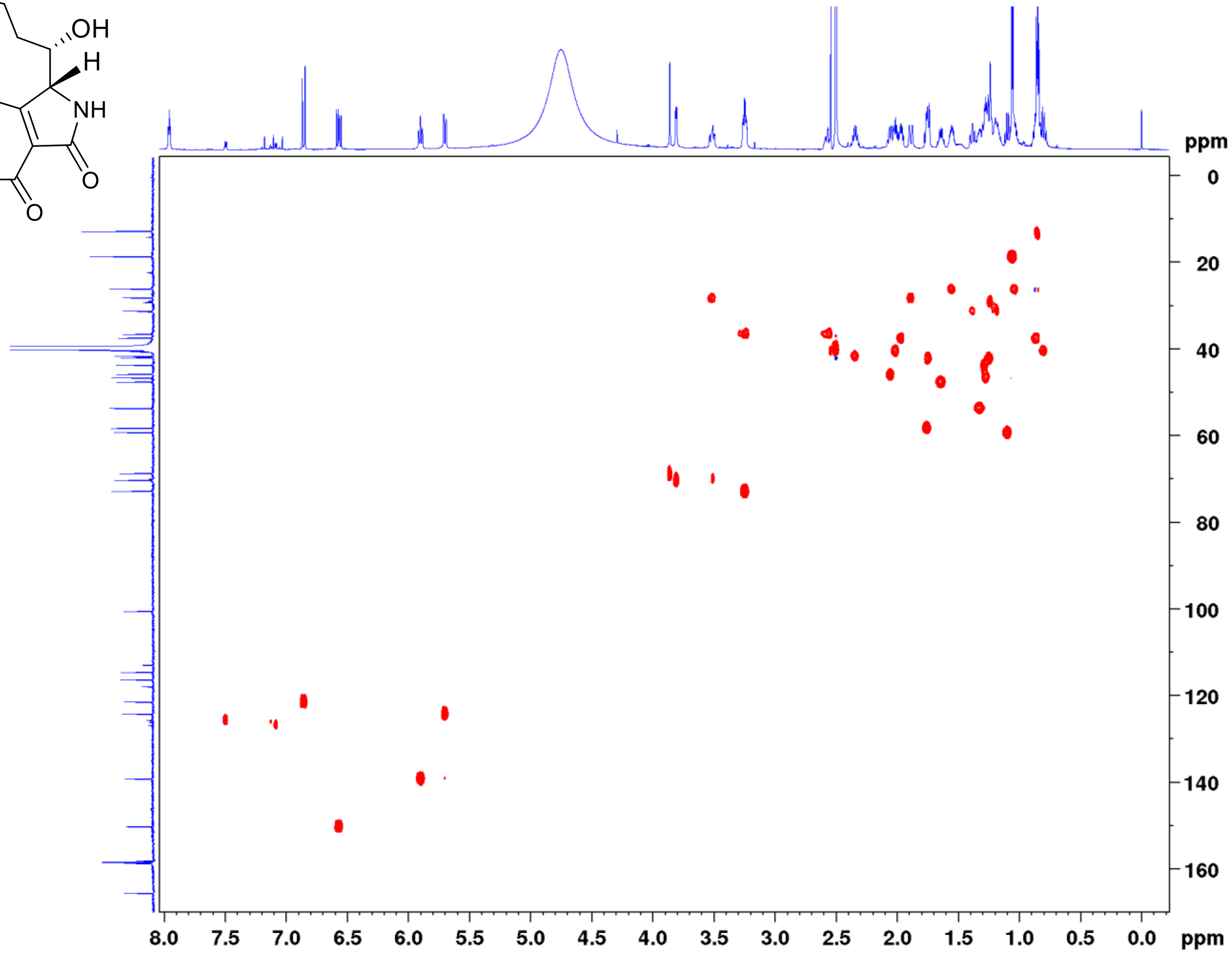

Figure S6. The HMBC spectrum of compound **1** in DMSO-d_6.

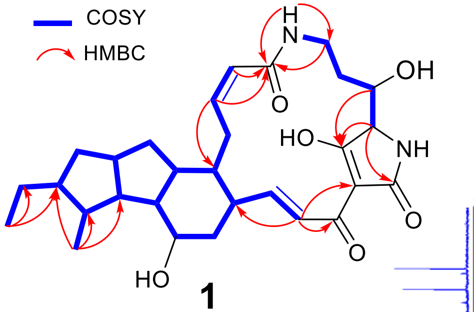

1

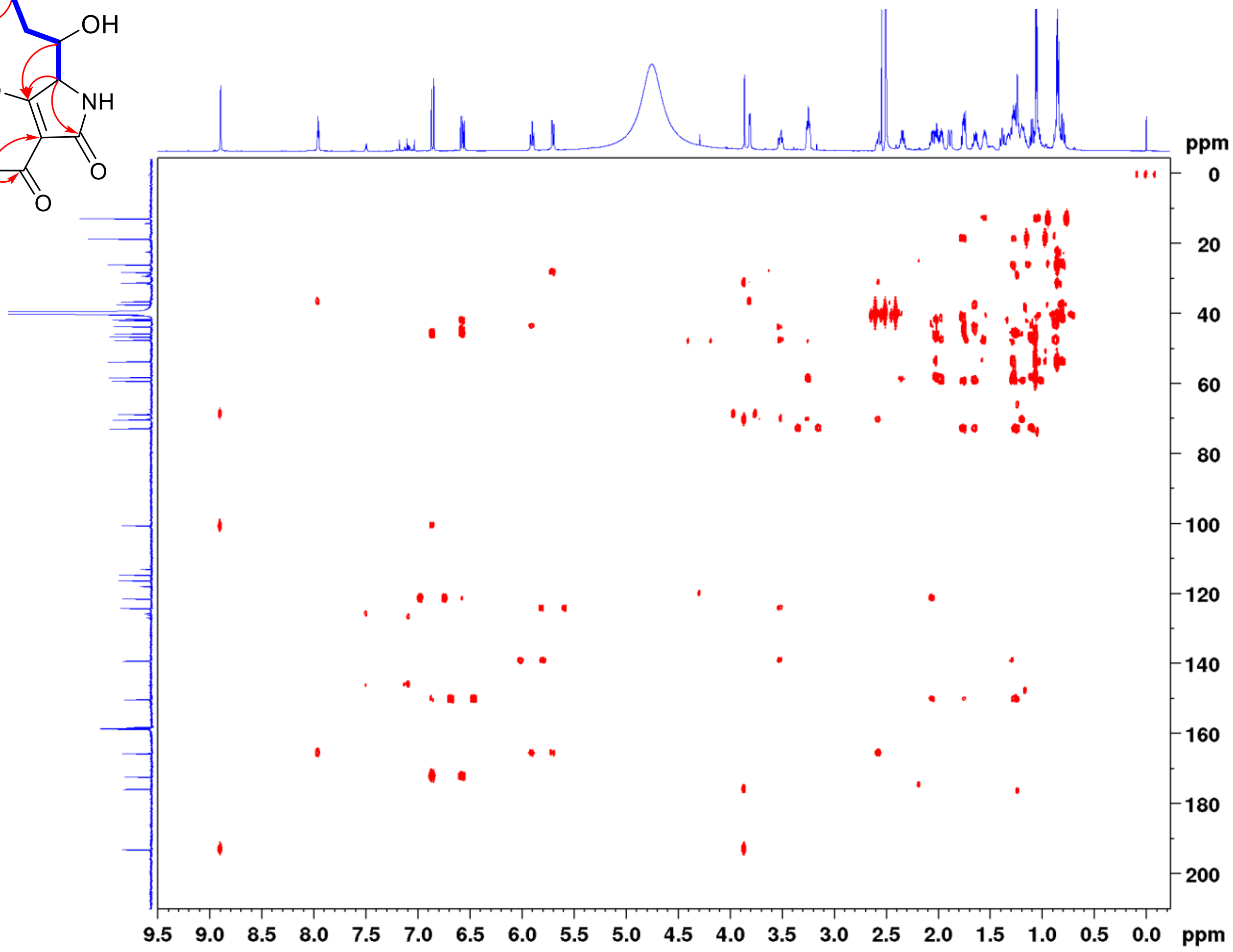

Figure S7. The ^{1}H-^{1}HCOSY spectrum of compound **1** in DMSO-d_6.

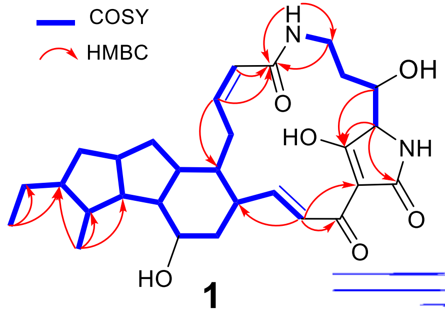

1

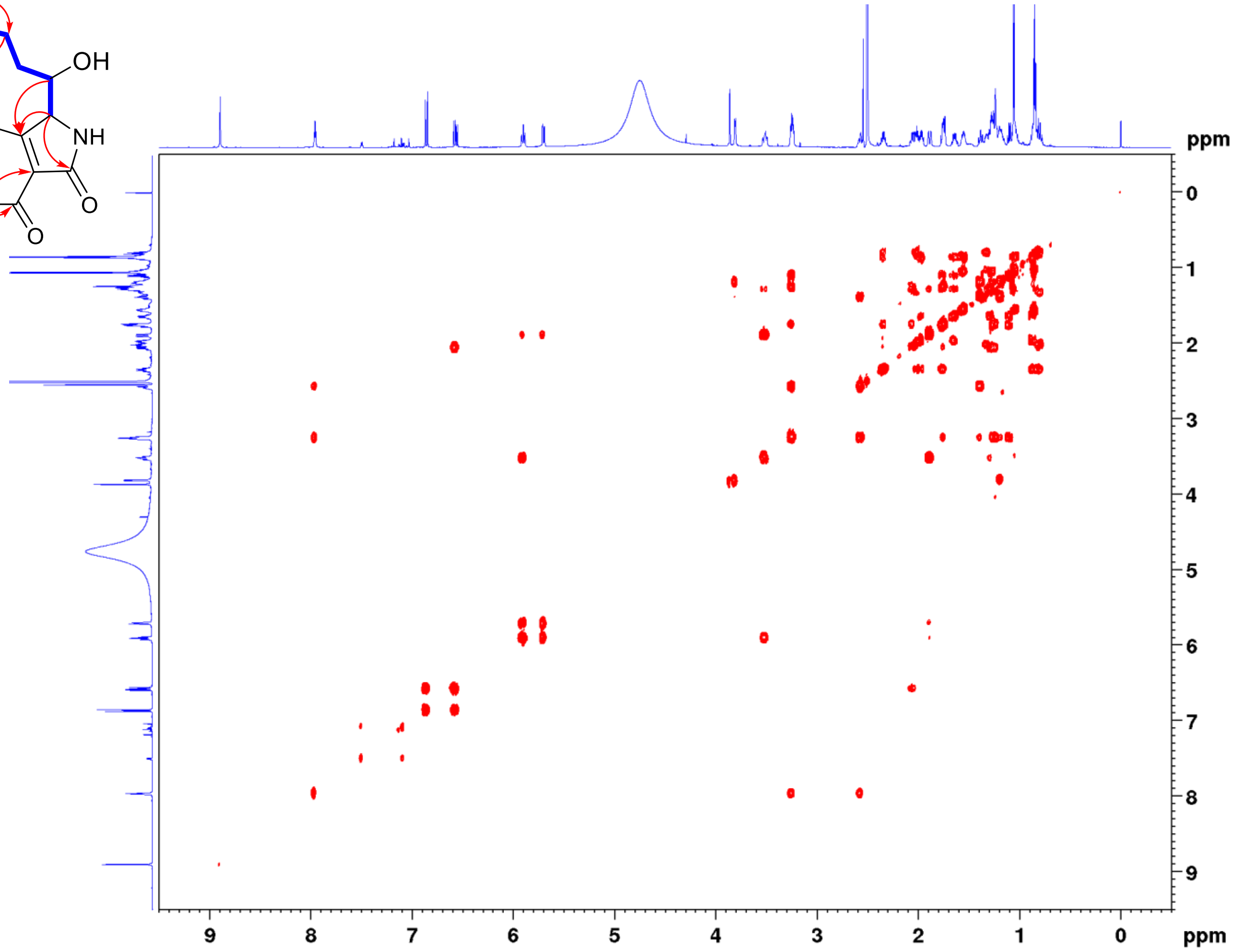

Figure S8. The NOESY spectrum of compound **1** in DMSO-d_6.

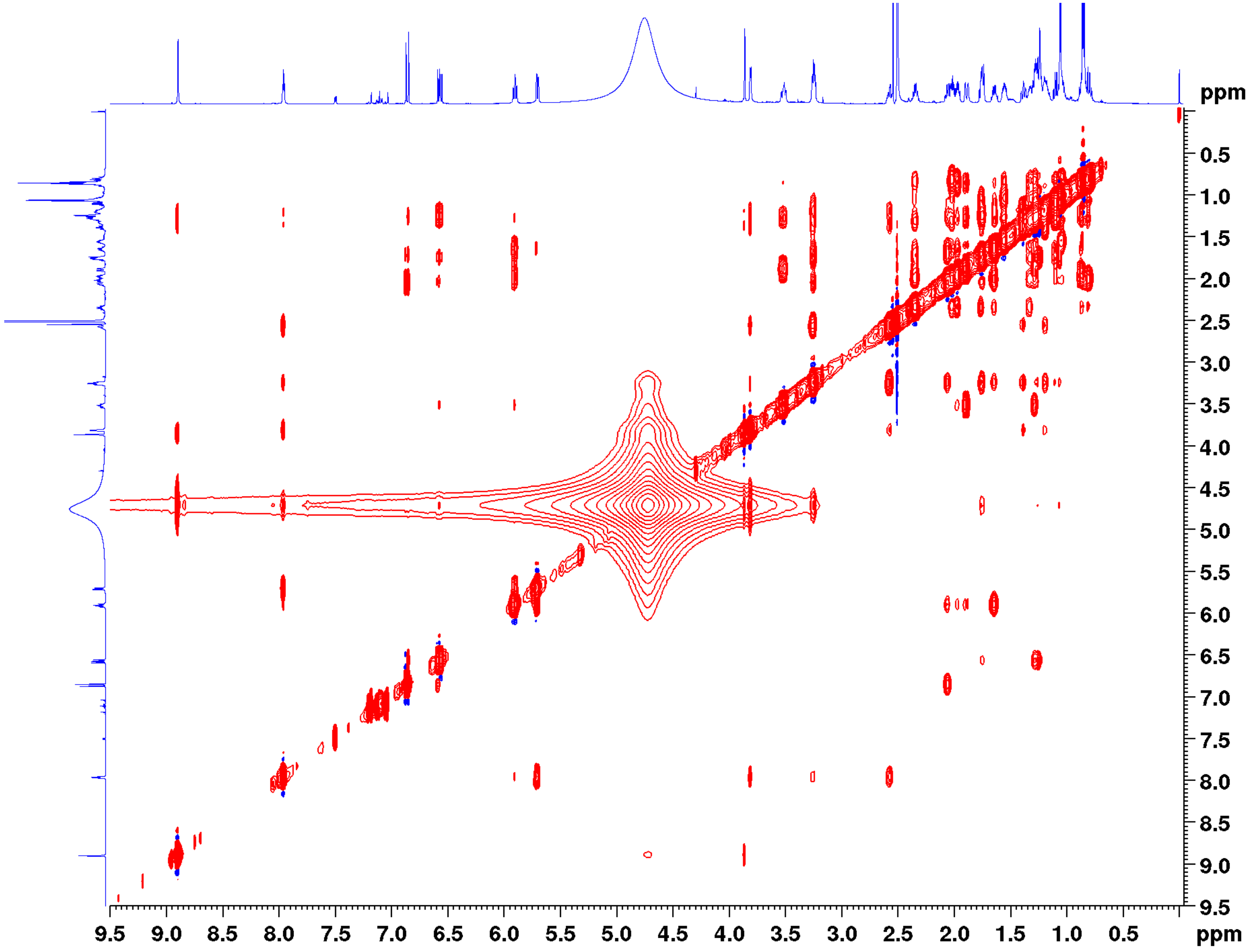

Figure S9. The key NOESY spectrum of compound **1** in DMSO-d_6.

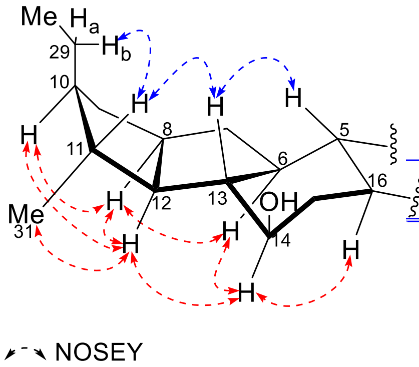

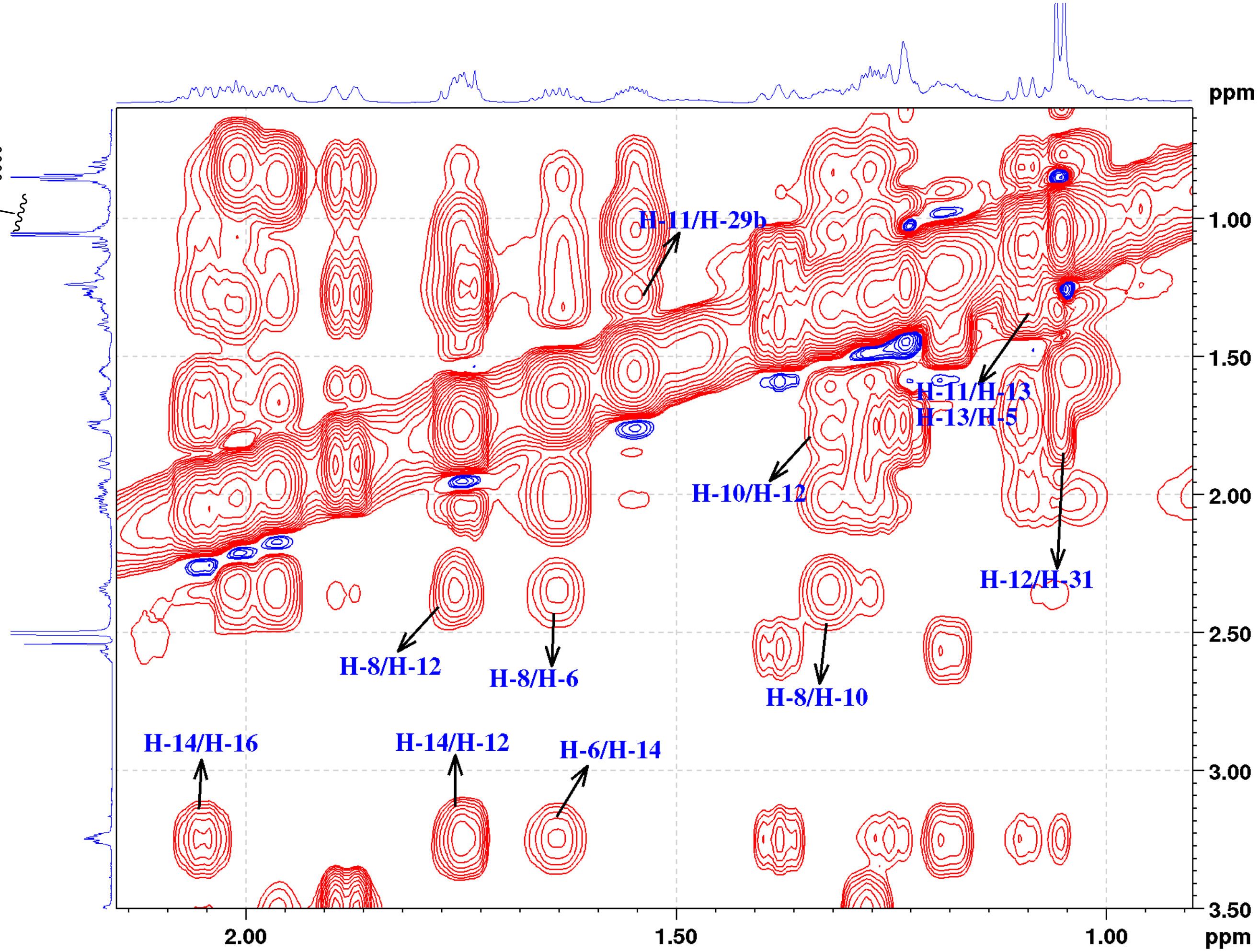

Figure S10. Comparison of ECD spectra of compound **1-6** and the known compounds.

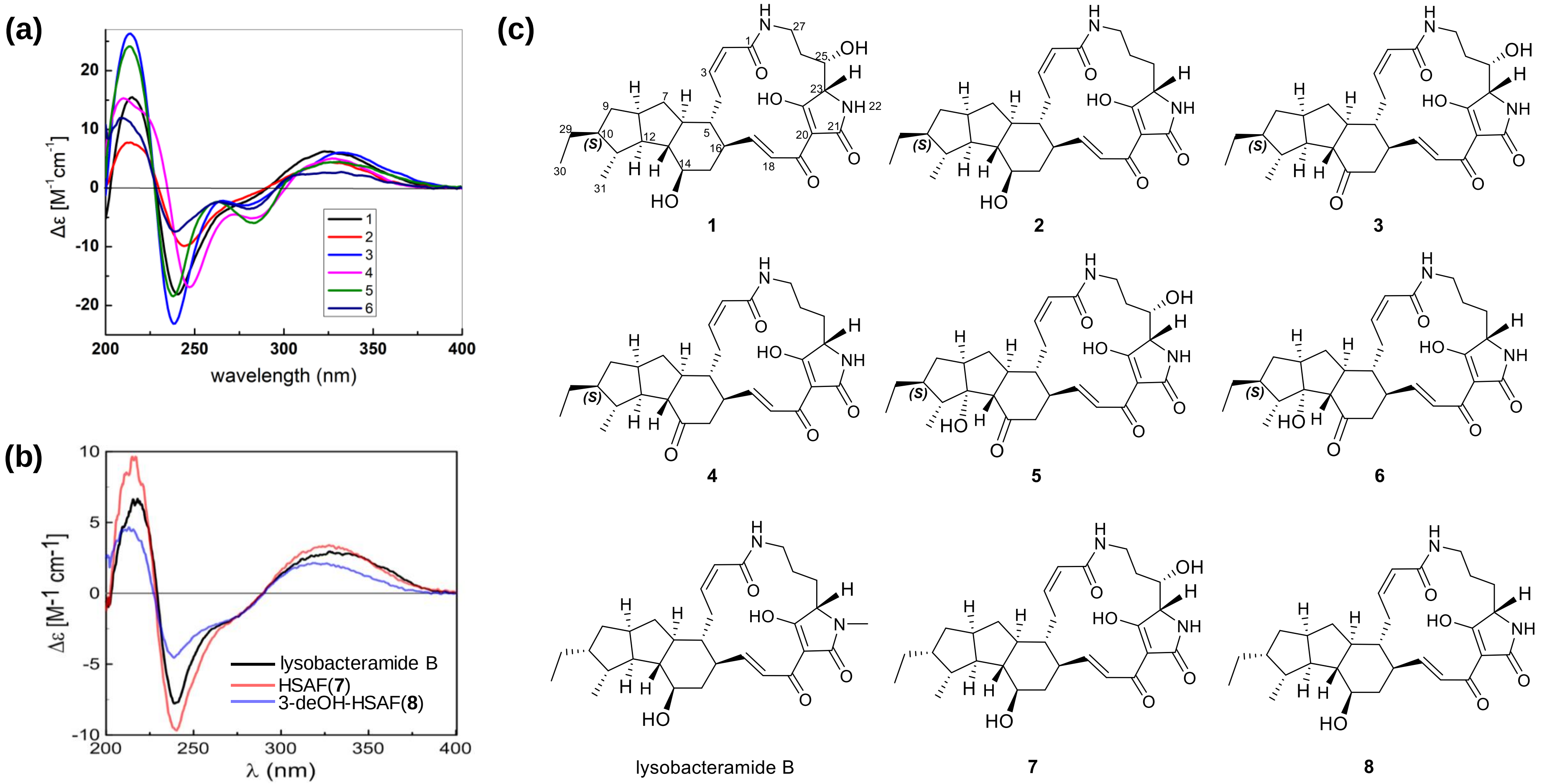

Note: **(a)** ECD spectra measured for compound **1-6**; **(b)** ECD spectra of lysobacteramide B, HSAF and 3-deOH-HSAF from reference; (**c**) Chemical structures.

Figure S11. HRESIMS (**a**) and IR(**b**) of compound **2**.

(a) **HRESIMS**

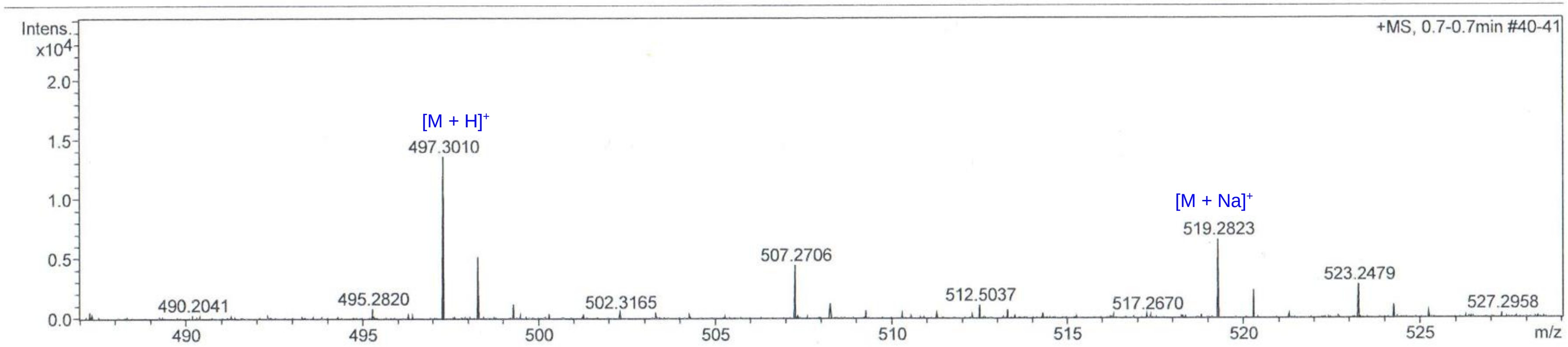

(b) **IR**

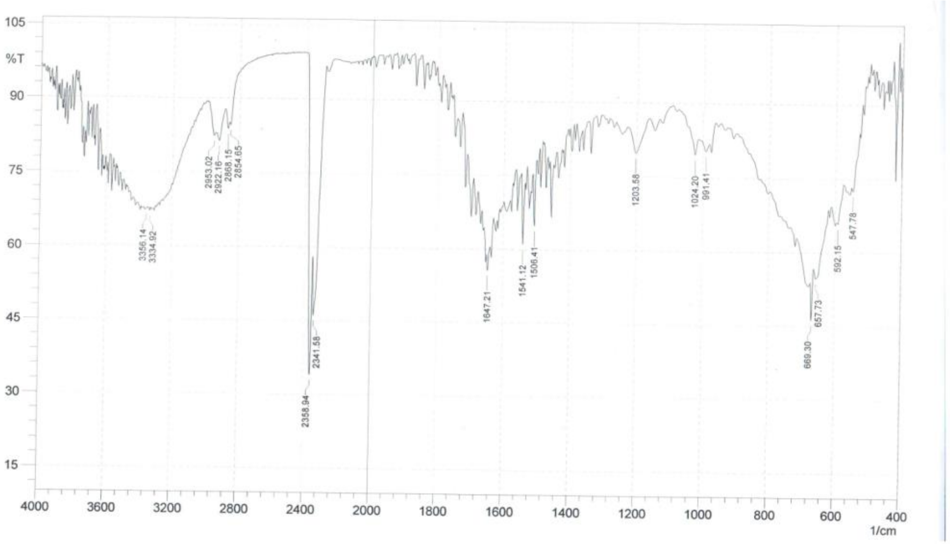

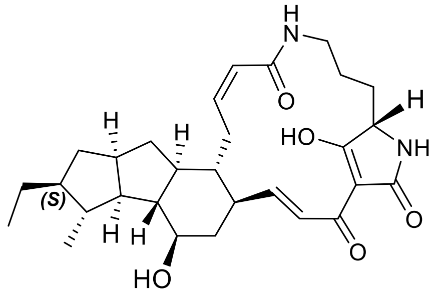

2

Chemical Formula: $C_{29}H_{40}N_2O_5$

caculated for $[M+H]^+$: 497.3015

caculated for $[M+Na]^+$: 519.2835

Figure S12. The ^{1}H NMR spectrum of compound **2** in DMSO-d_6.

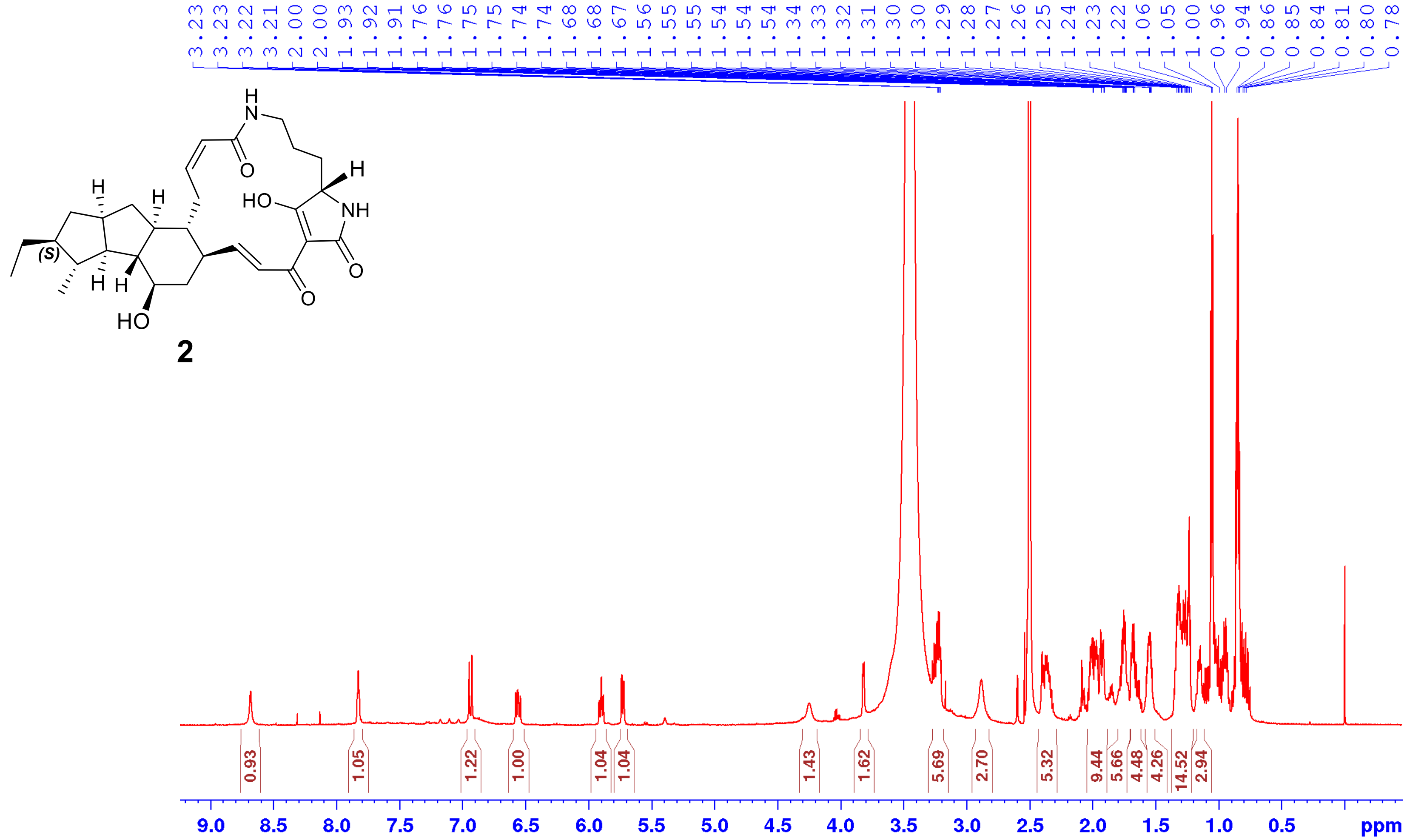

Figure S13. The ^{13}C NMR and DEPT 135 spectra of compound **2** in DMSO-d_6.

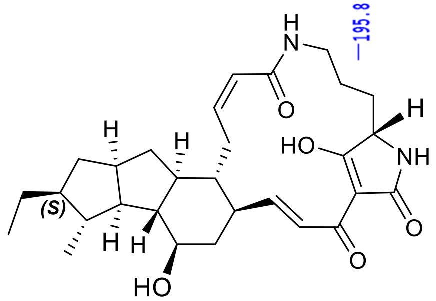

2

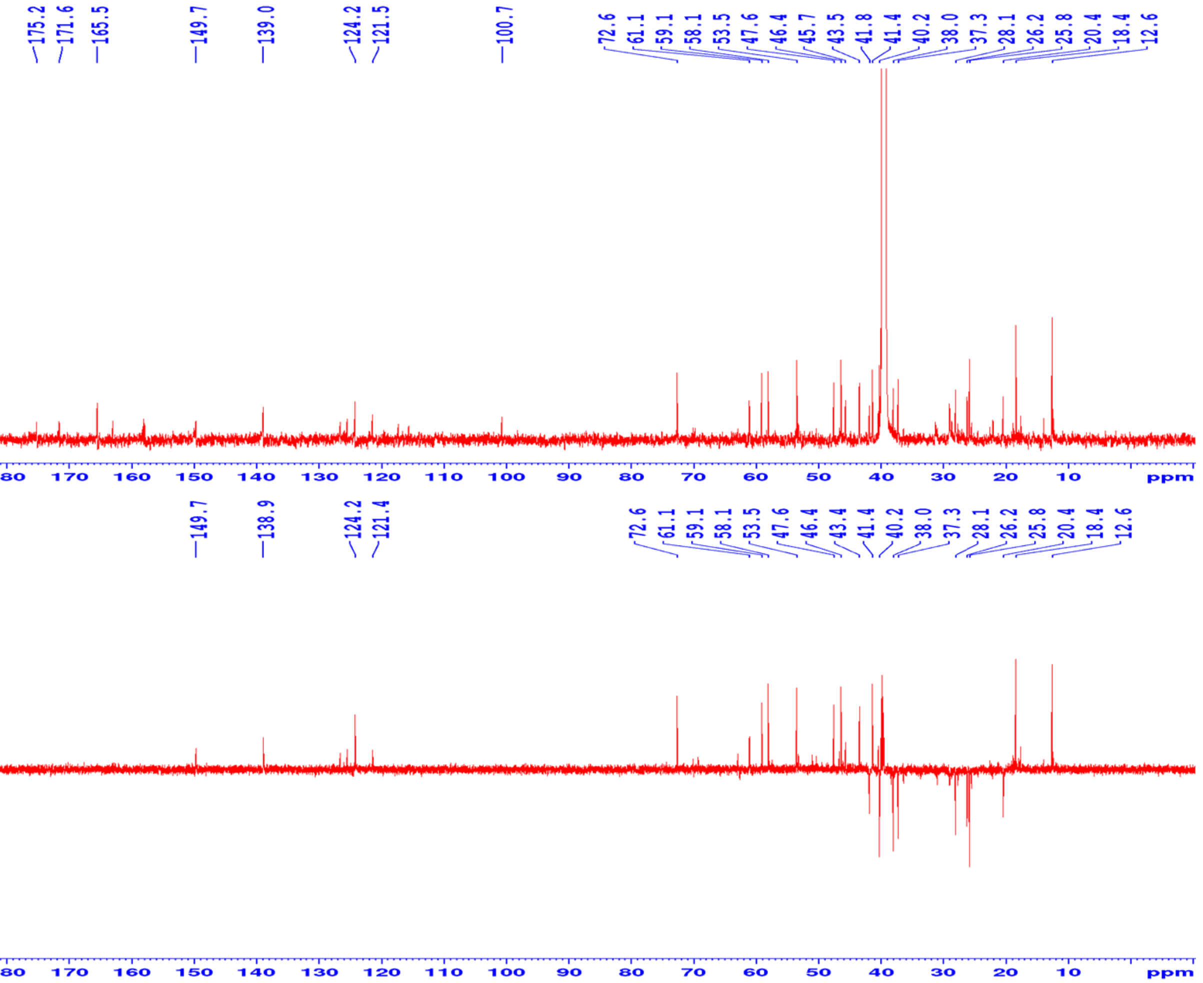

Figure S14. The HSQC spectrum of compound **2** in DMSO-d_6.

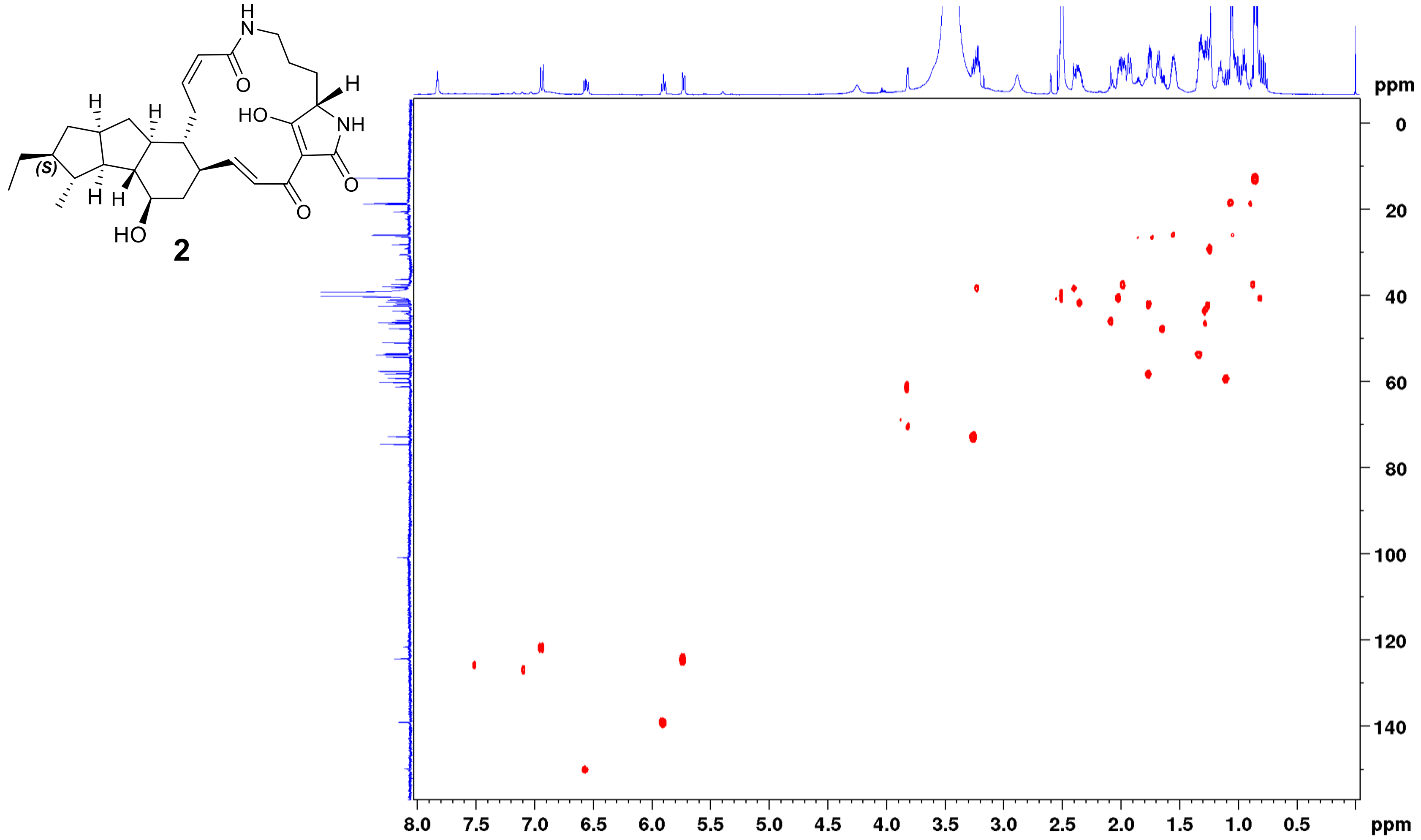

Figure S15. The HMBC spectrum of compound **2** in DMSO-d_6.

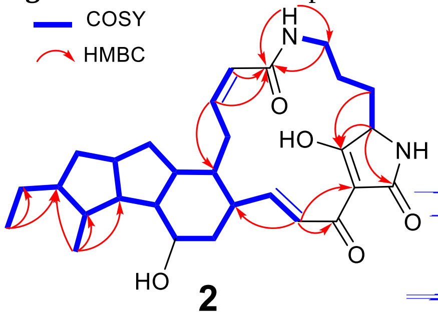

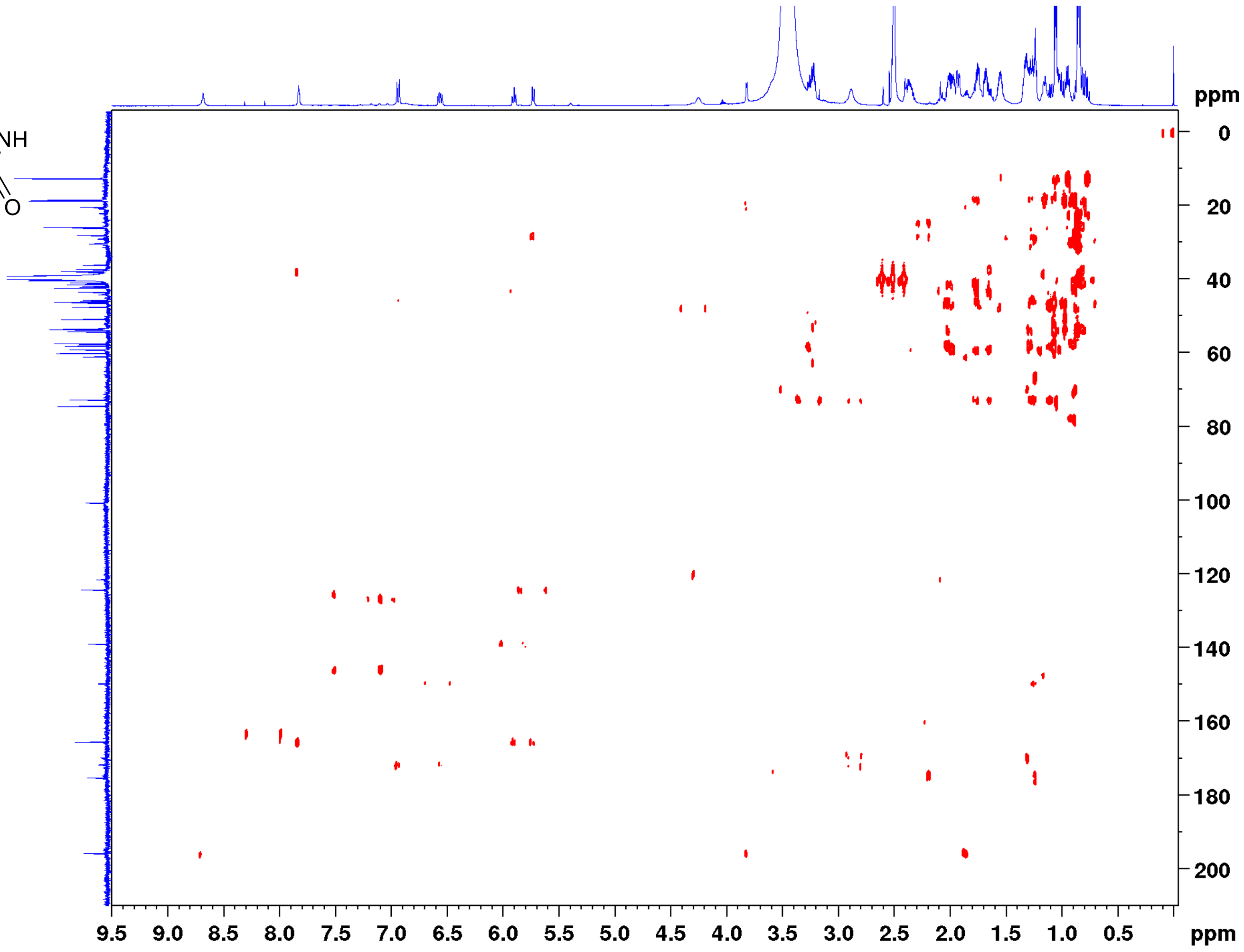

Figure S16. The ^{1}H-^{1}HCOSY spectrum of compound **2** in DMSO-d_6.

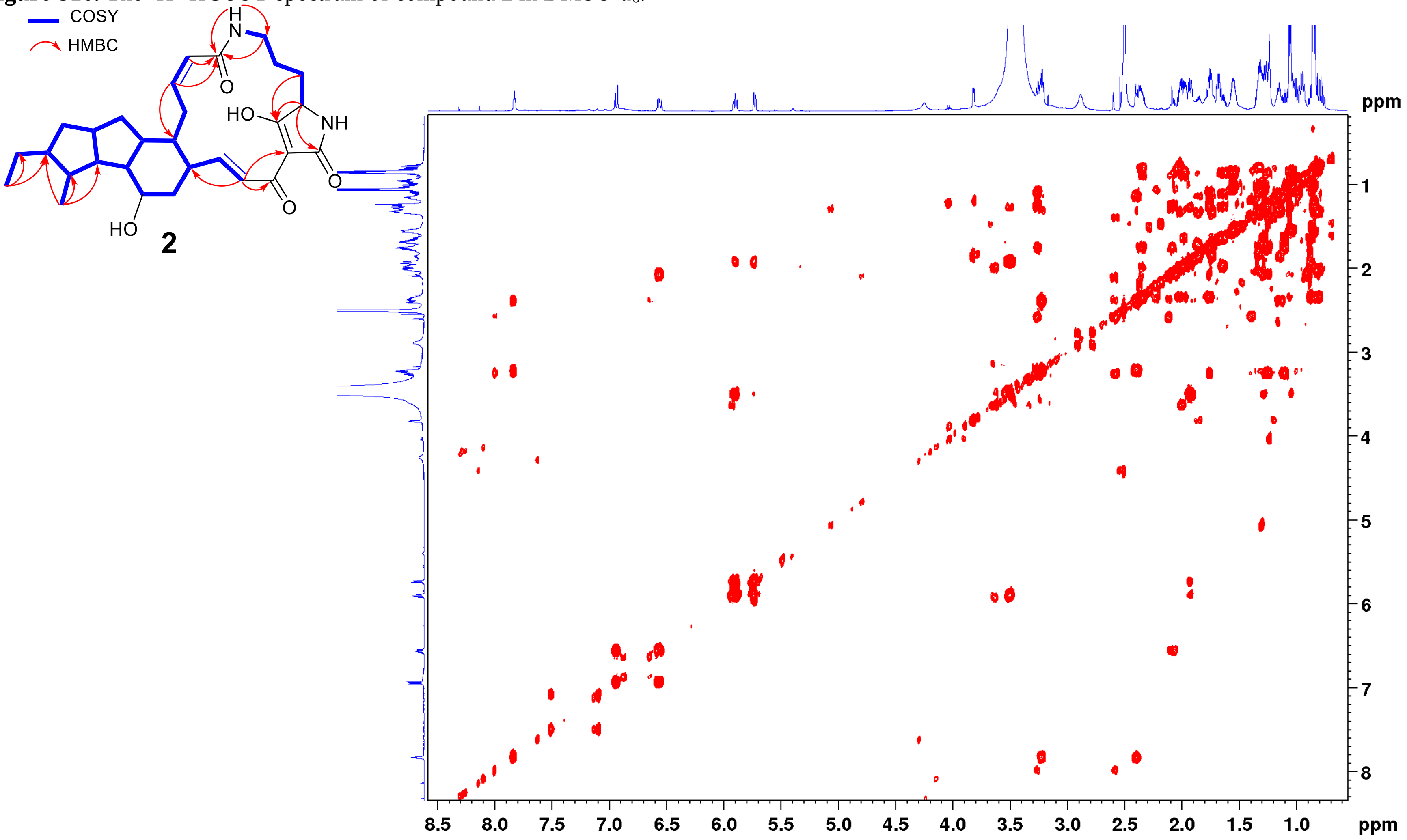

Figure S17. The NOESY spectrum of compound **2** in DMSO-d_6.

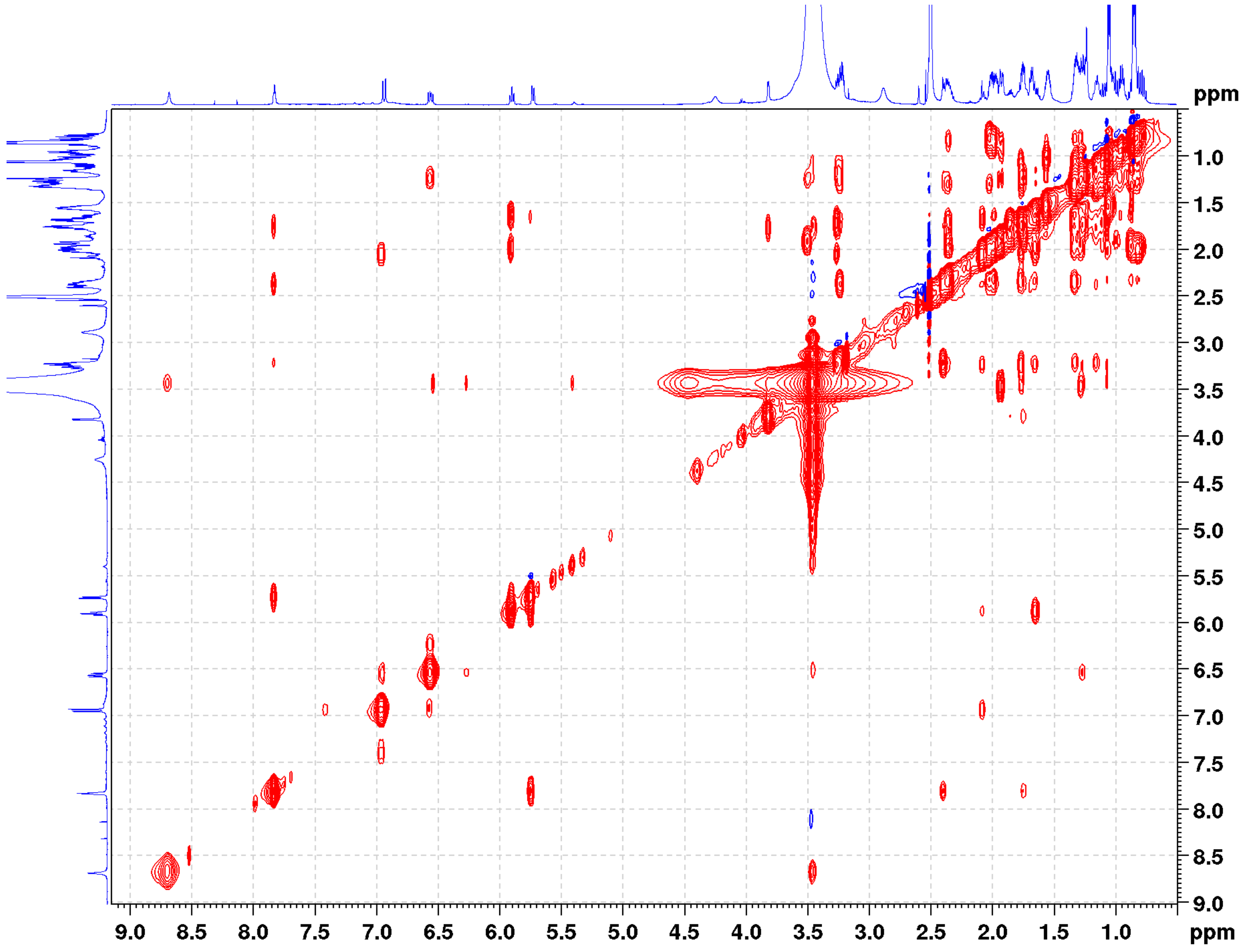

Figure S18. The key NOESY spectrum of compound **2** in DMSO-d_6.

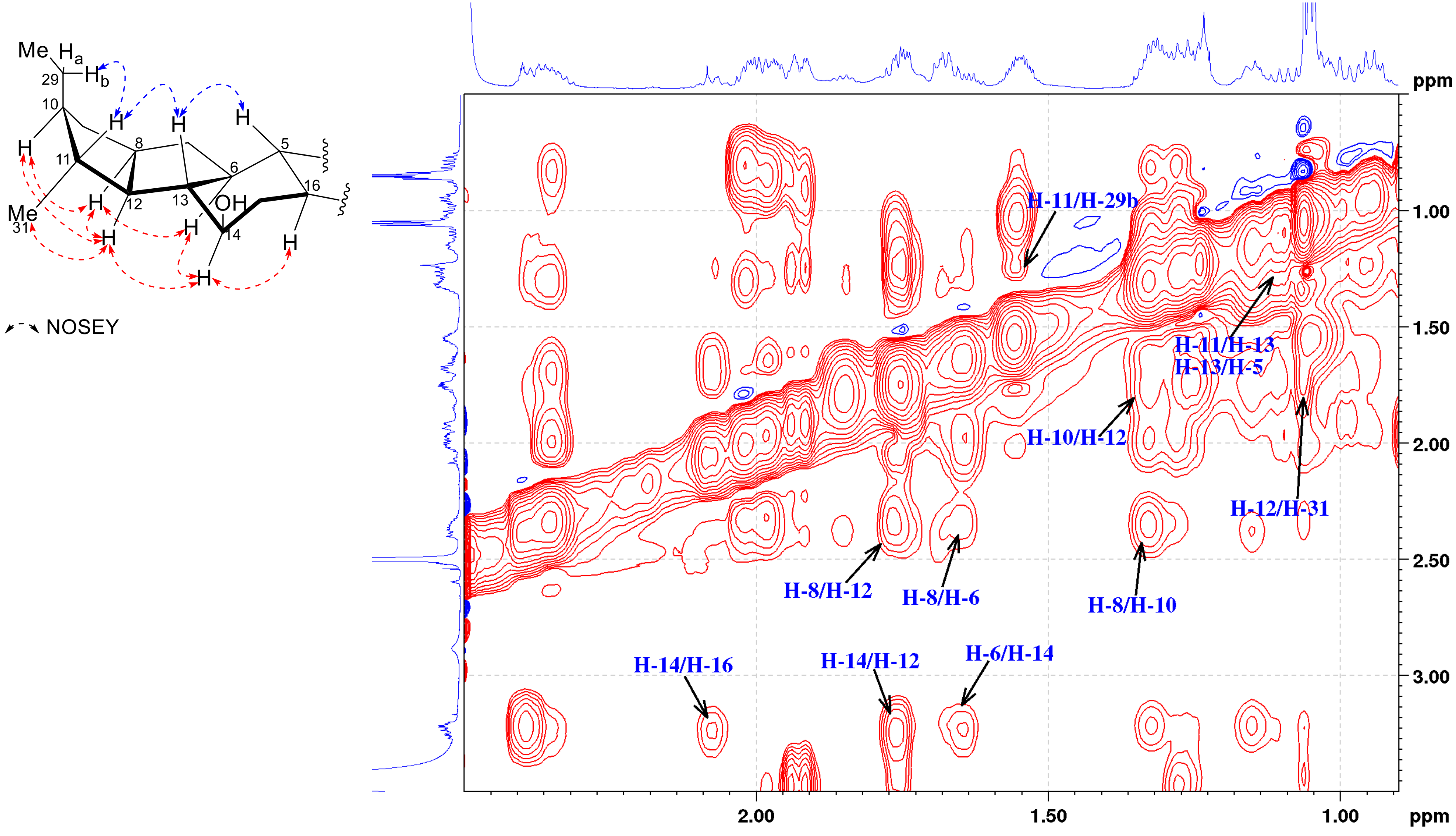

Figure S19. HRESIMS (**a**) and IR (**b**) of compound **3**.

(**a**) **HRESIMS**

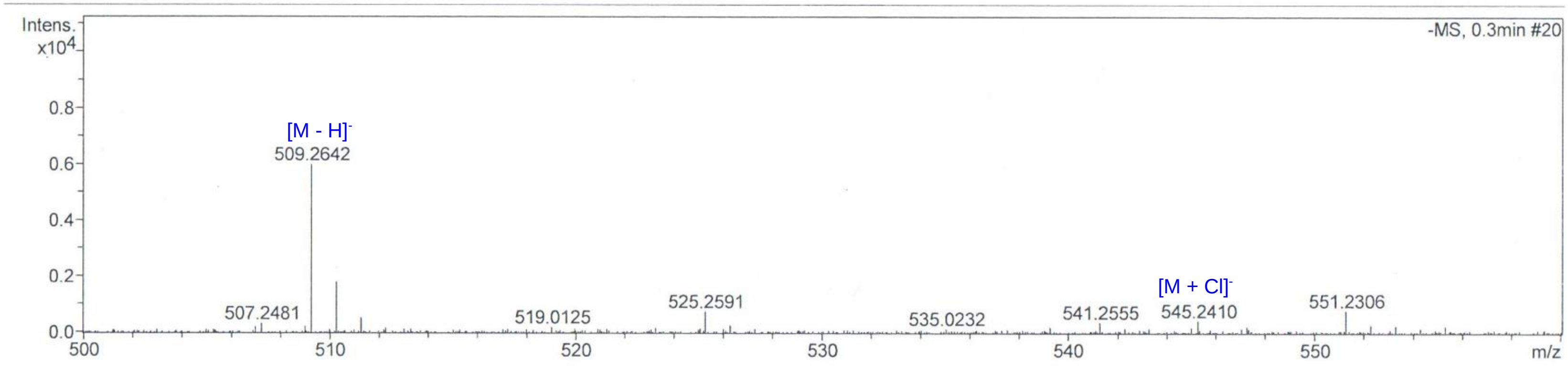

(**b**) **IR**

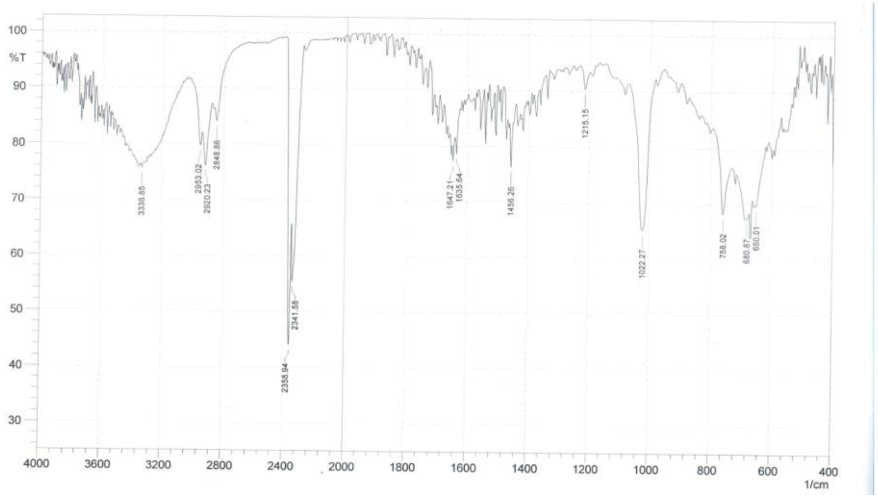

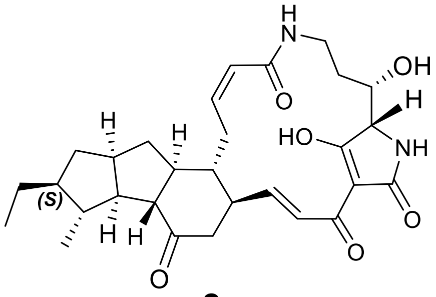

3

Chemical Formula: $C_{29}H_{38}N_2O_6$

caculated for $[M-H]^-$: 509.2652

caculated for $[M+Cl]^-$: 545.2418

Figure S20. The ^{1}H NMR spectrum of compound **3** in DMSO-d_6.

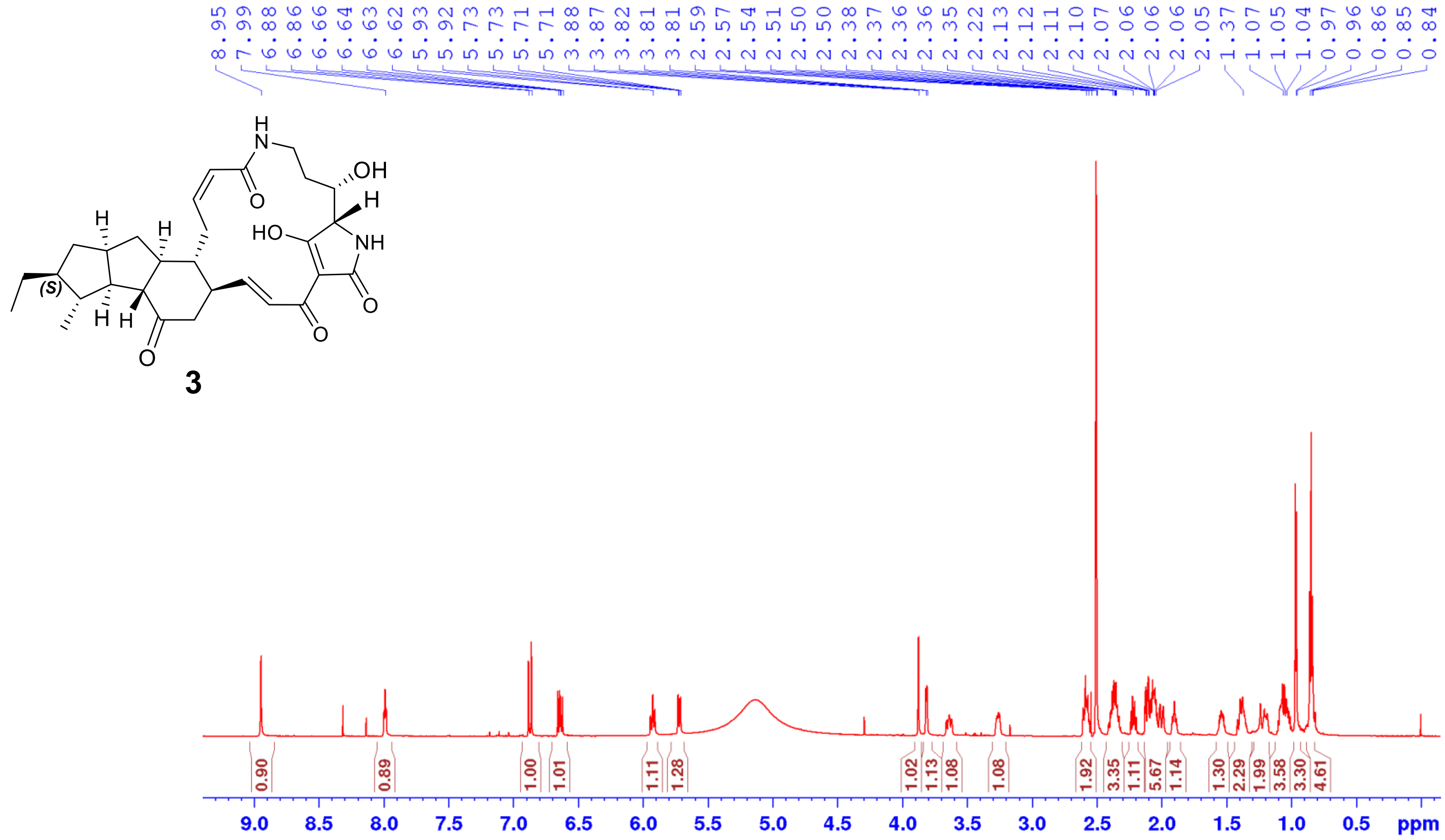

Figure S21. The ^{13}C NMR and DEPT 135 spectra of compound **3** in DMSO-d_6.

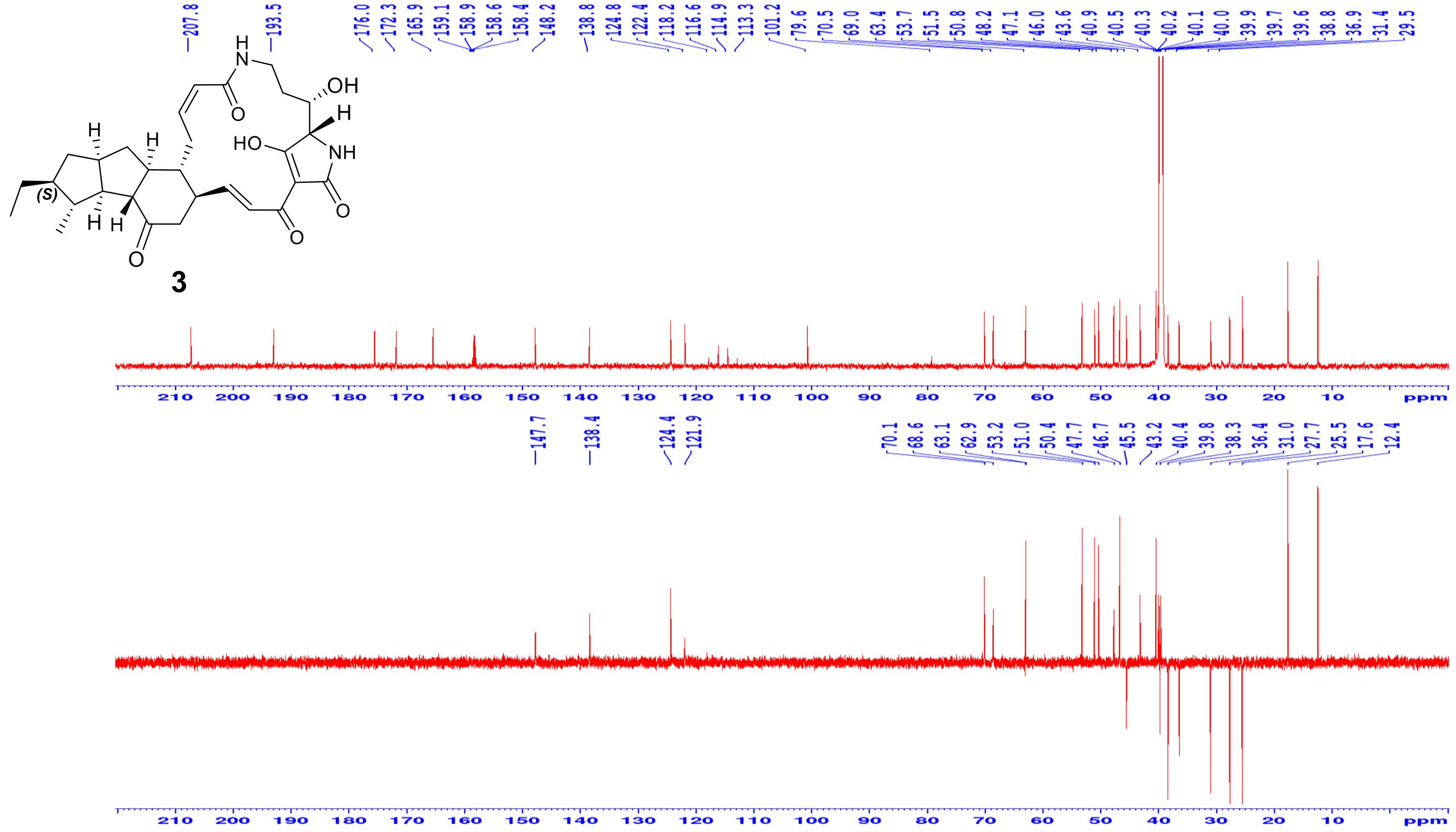

Figure S22. The HSQC spectrum of compound **3** in DMSO-d_6.

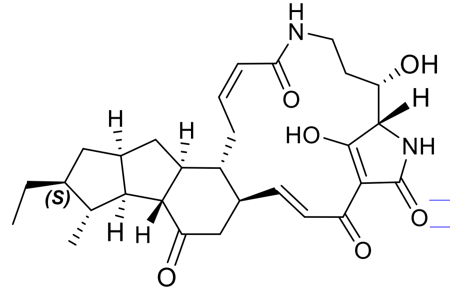

3

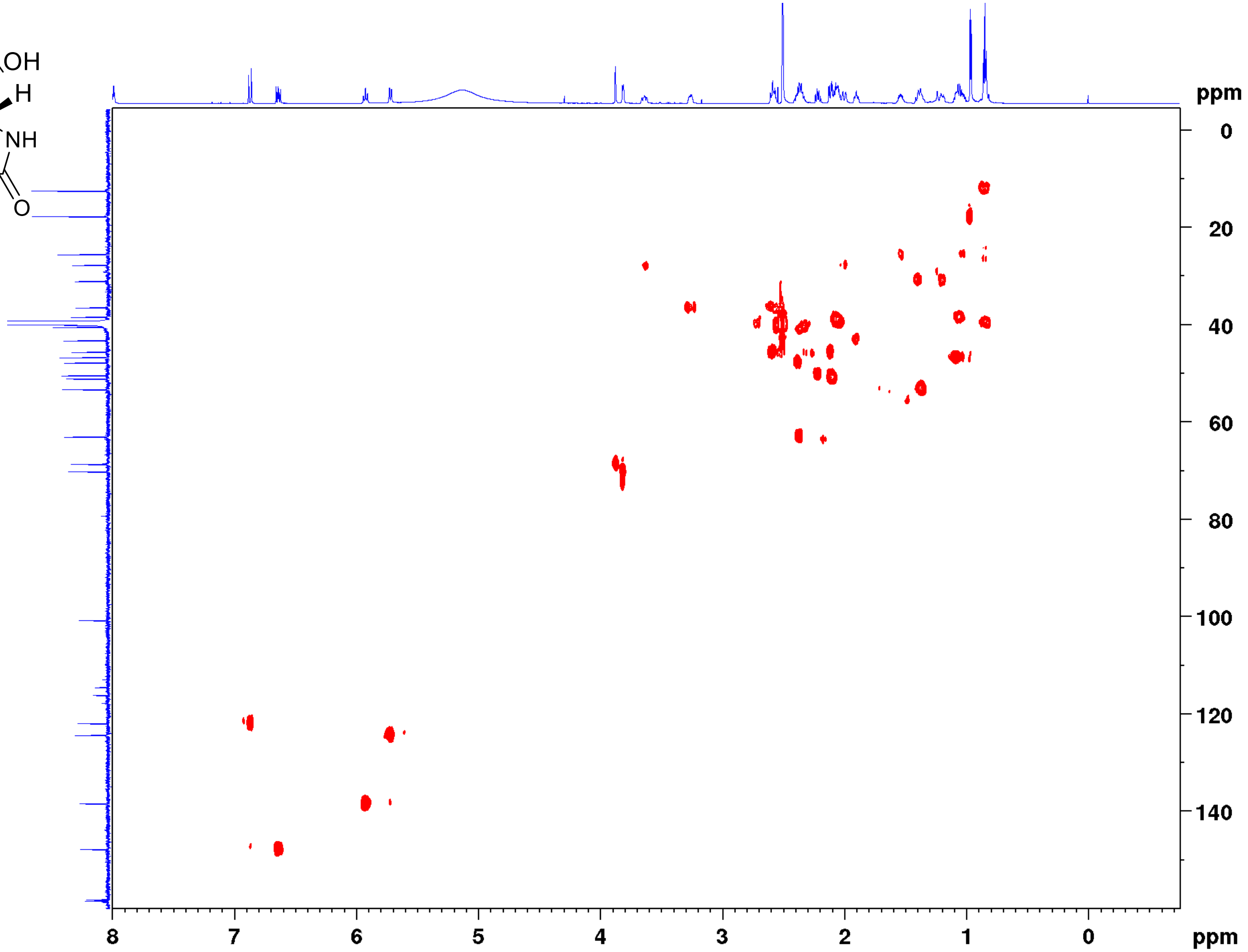

Figure S23. The HMBC spectrum of compound **3** in DMSO-d_6.

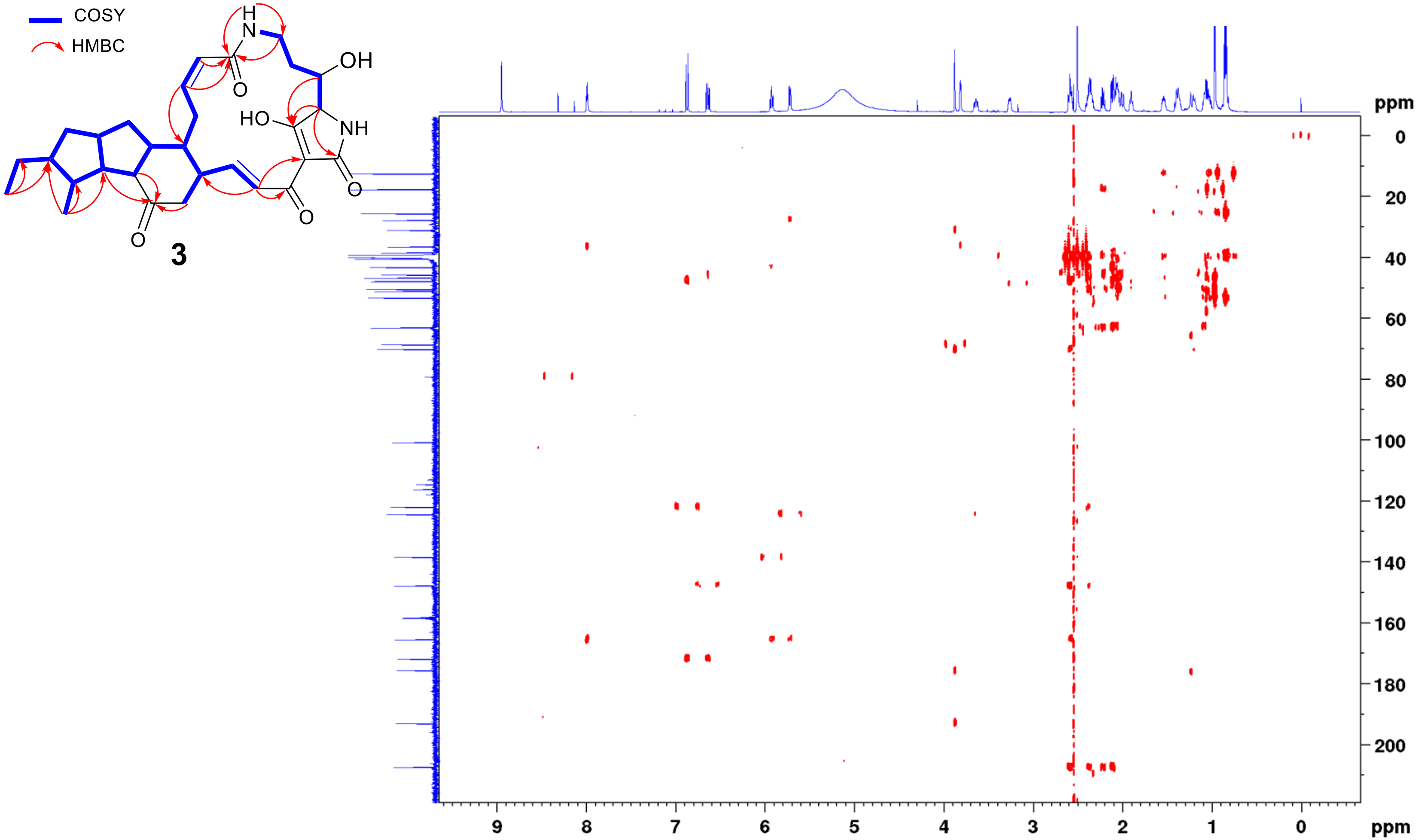

Figure S24. The ^{1}H-[1] HCOSY spectrum of compound **3** in DMSO-d_6.

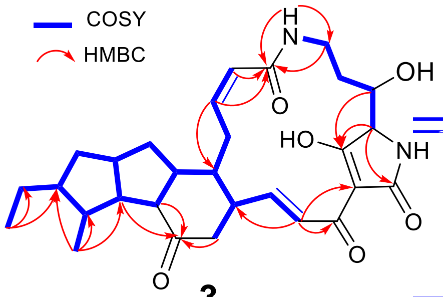

3

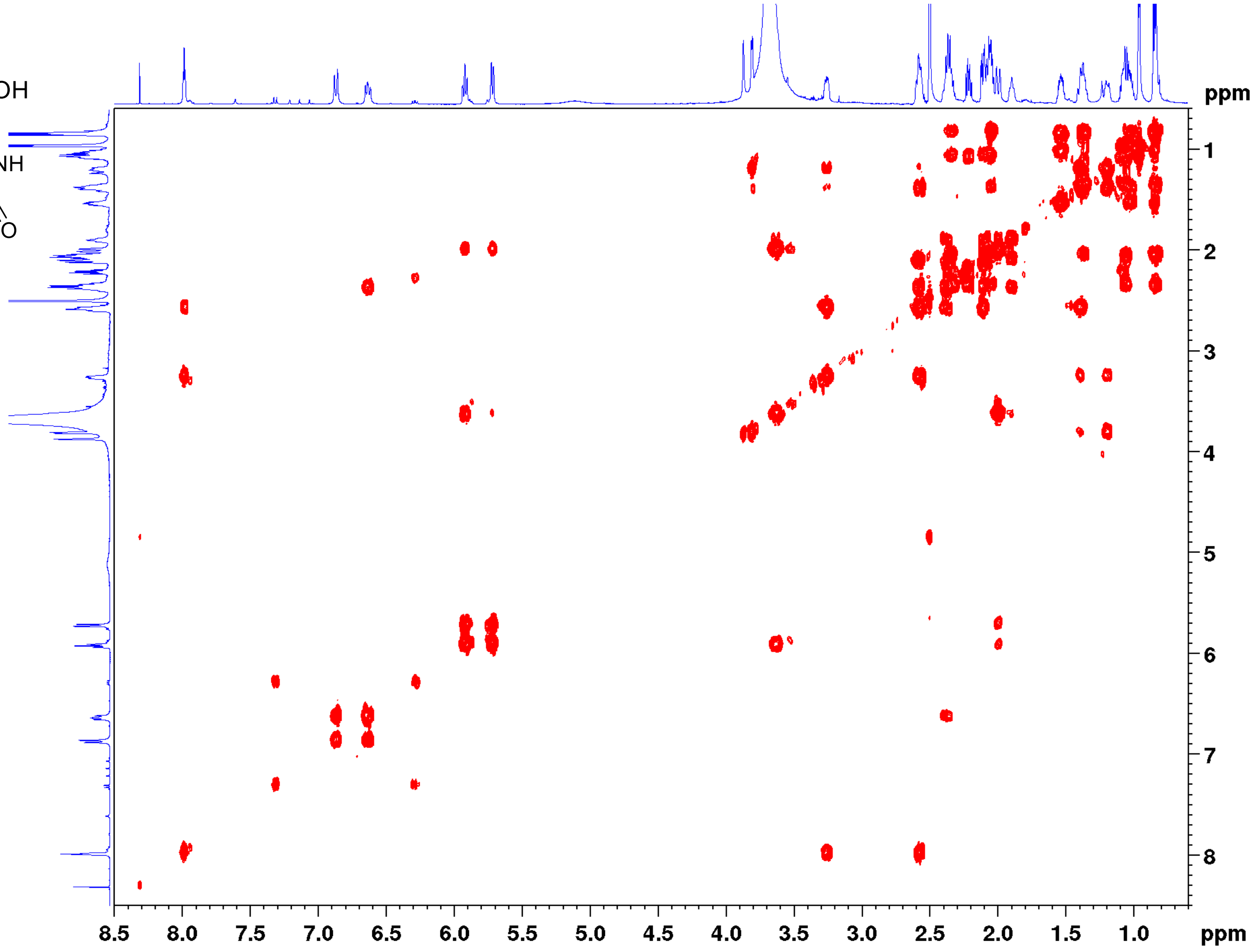

Figure S25. The NOESY spectrum of compound **3** in DMSO-d_6.

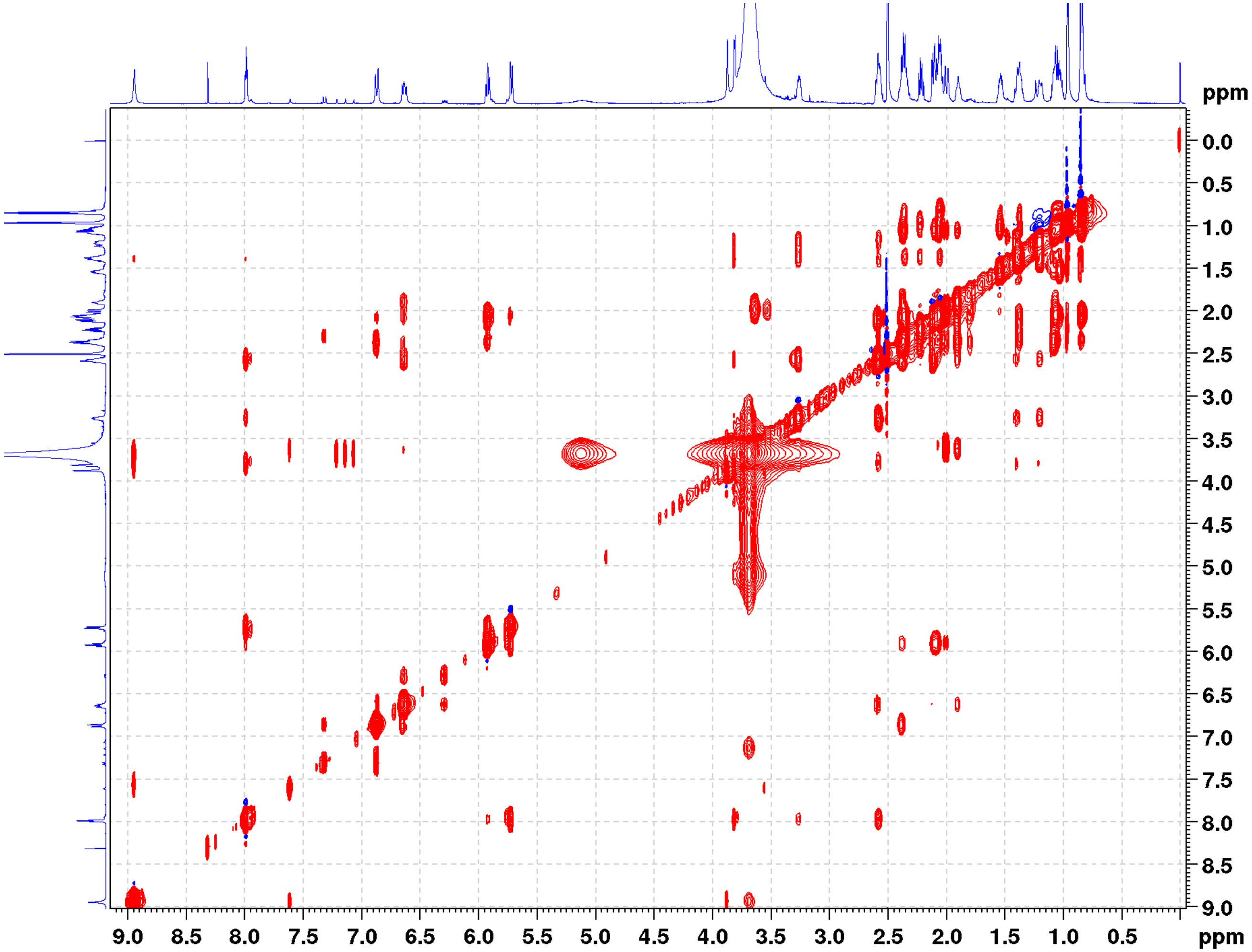

Figure S26. The key NOESY spectrum of compound **3** in DMSO-d_6.

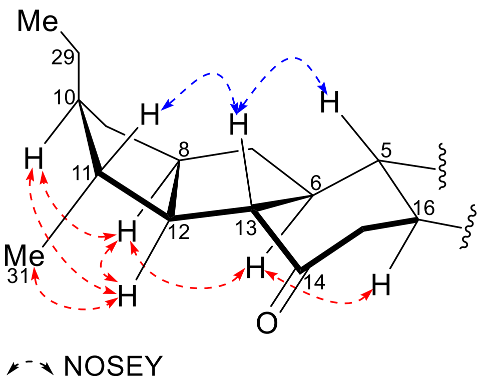

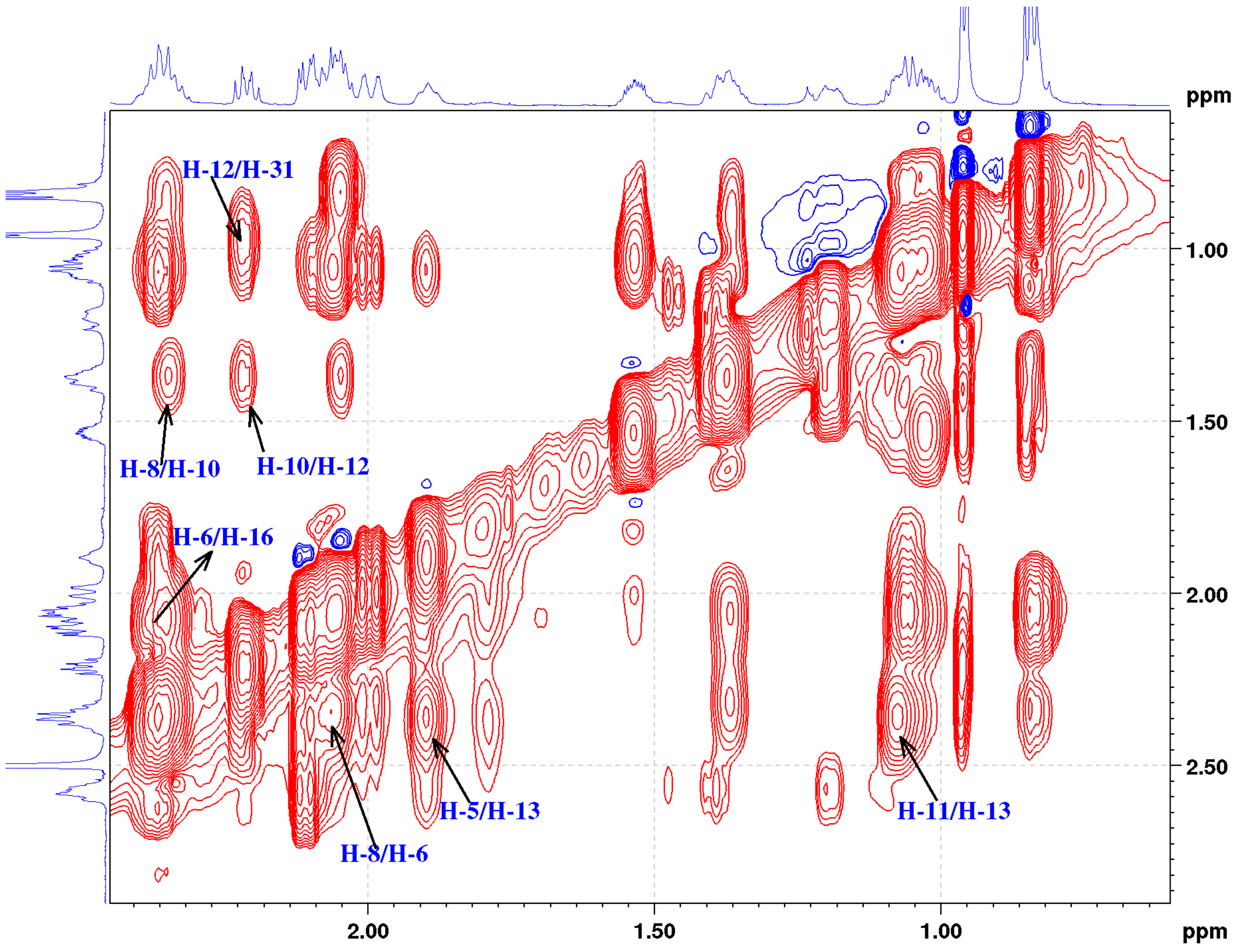

Figure S27. HRESIMS (**a**) and IR (**b**) of compound **4**.

(a) HRESIMS

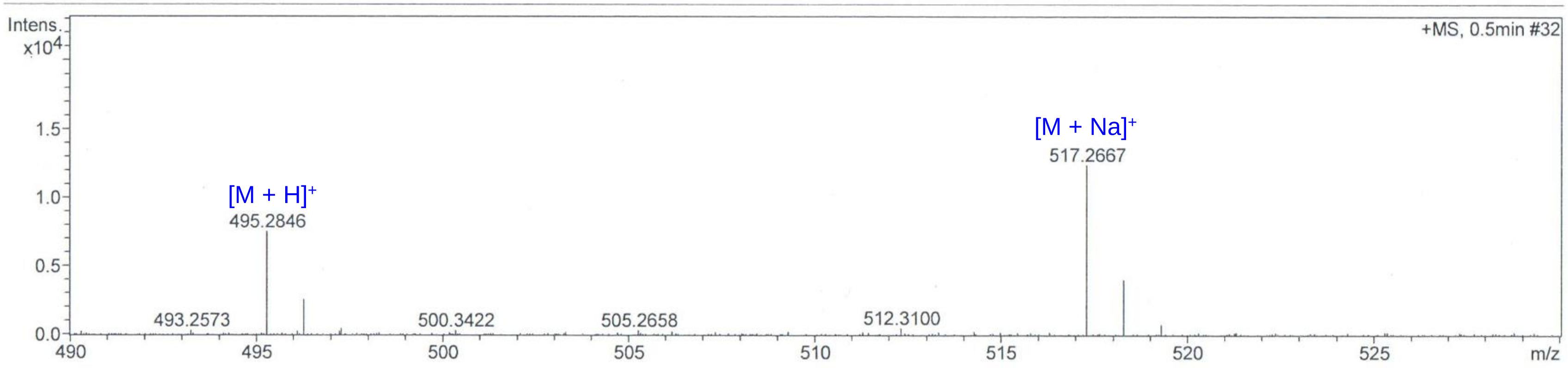

(b) IR

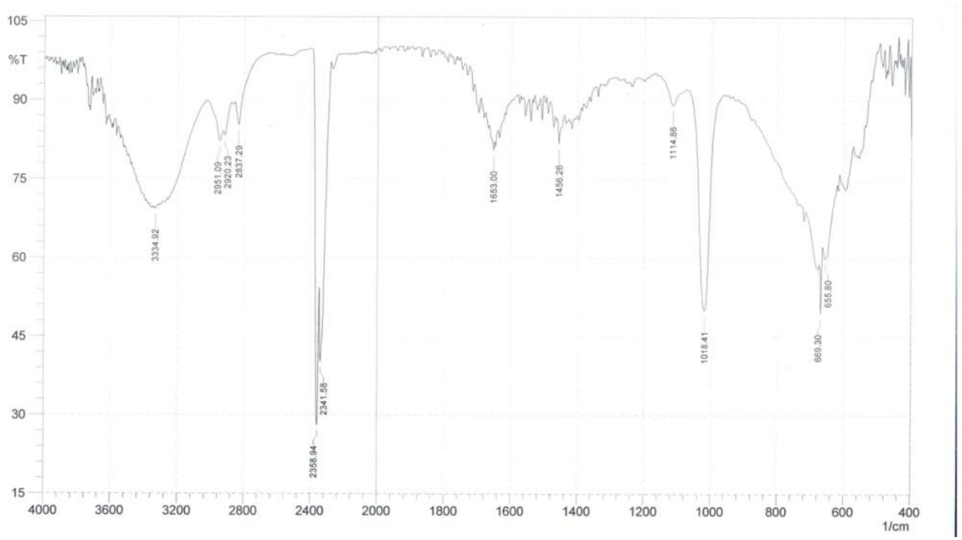

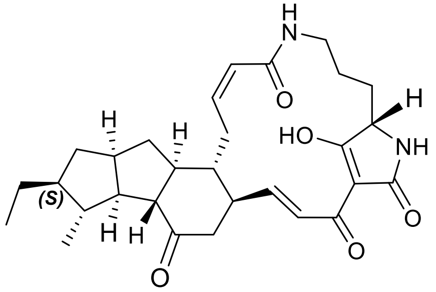

4

Chemical Formula: $C_{29}H_{38}N_2O_5$

caculated for $[M+H]^+$:495.2859

caculated for $[M+H]^+$:517.2678

Figure S28. The ^{1}H NMR spectrum of compound **4** in DMSO-d_6.

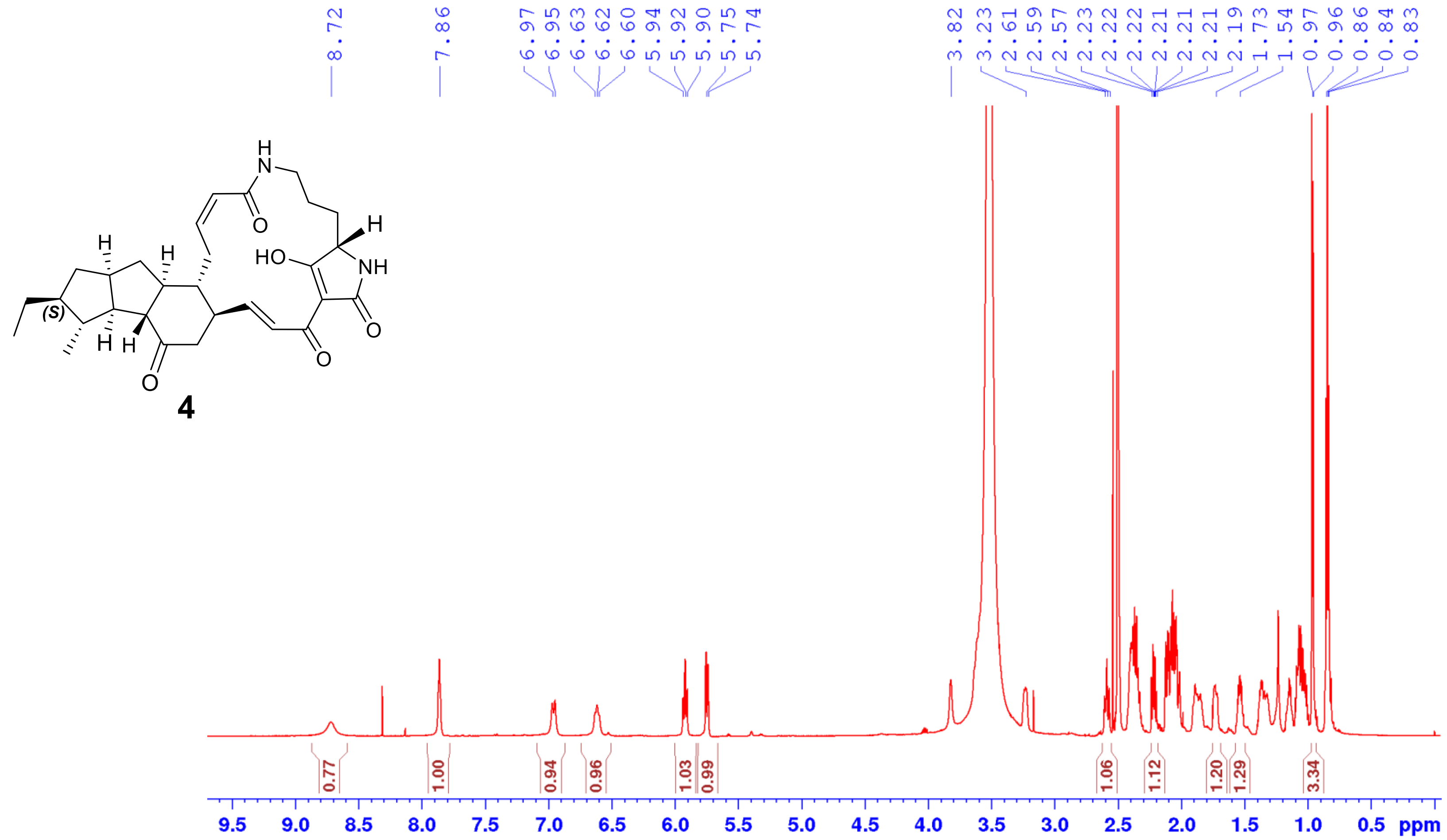

Figure S29. The ^{13}C NMR and DEPT 135 spectra of compound **4** in DMSO-d_6.

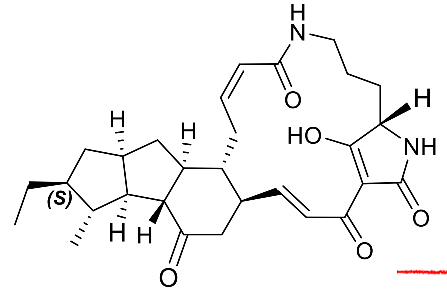

4

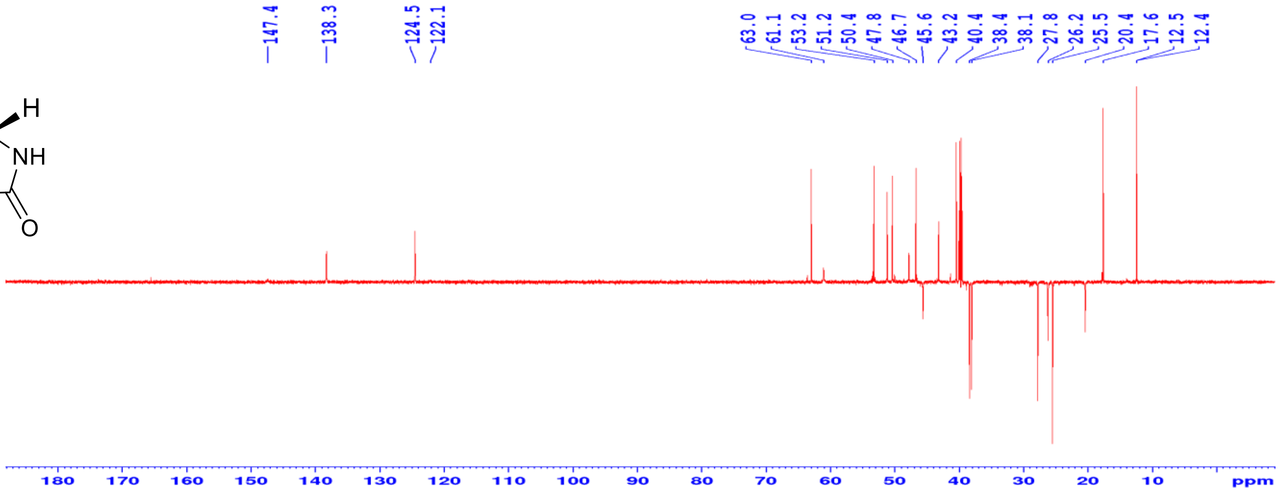

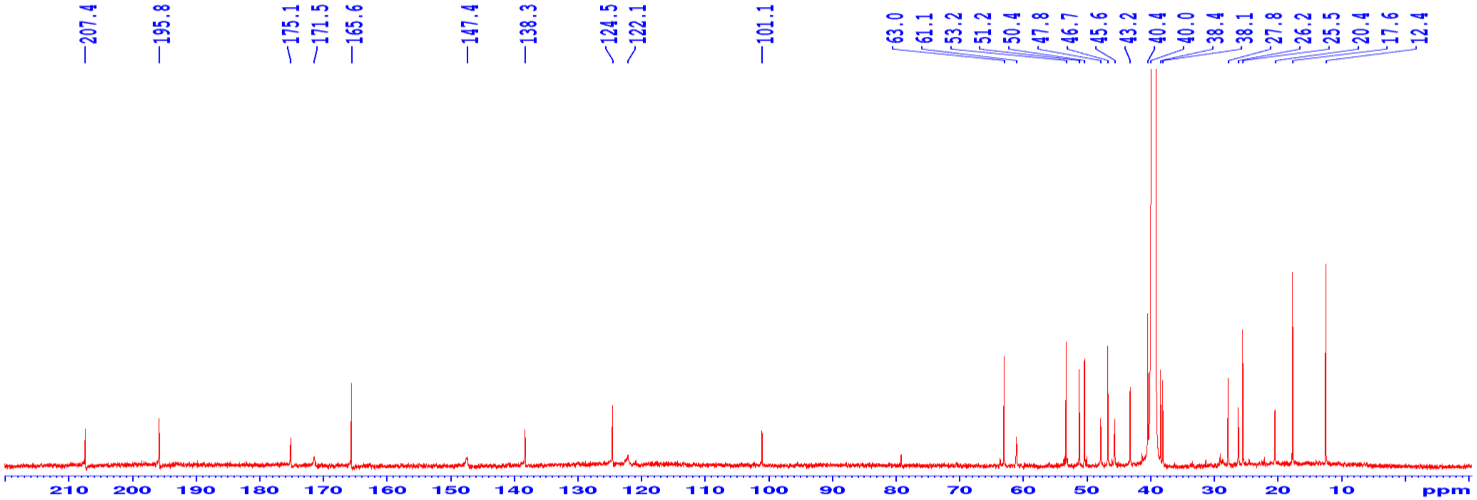

Figure S30. The HSQC spectrum of compound **4** in DMSO-d_6.

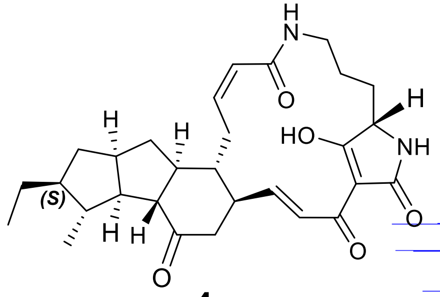

4

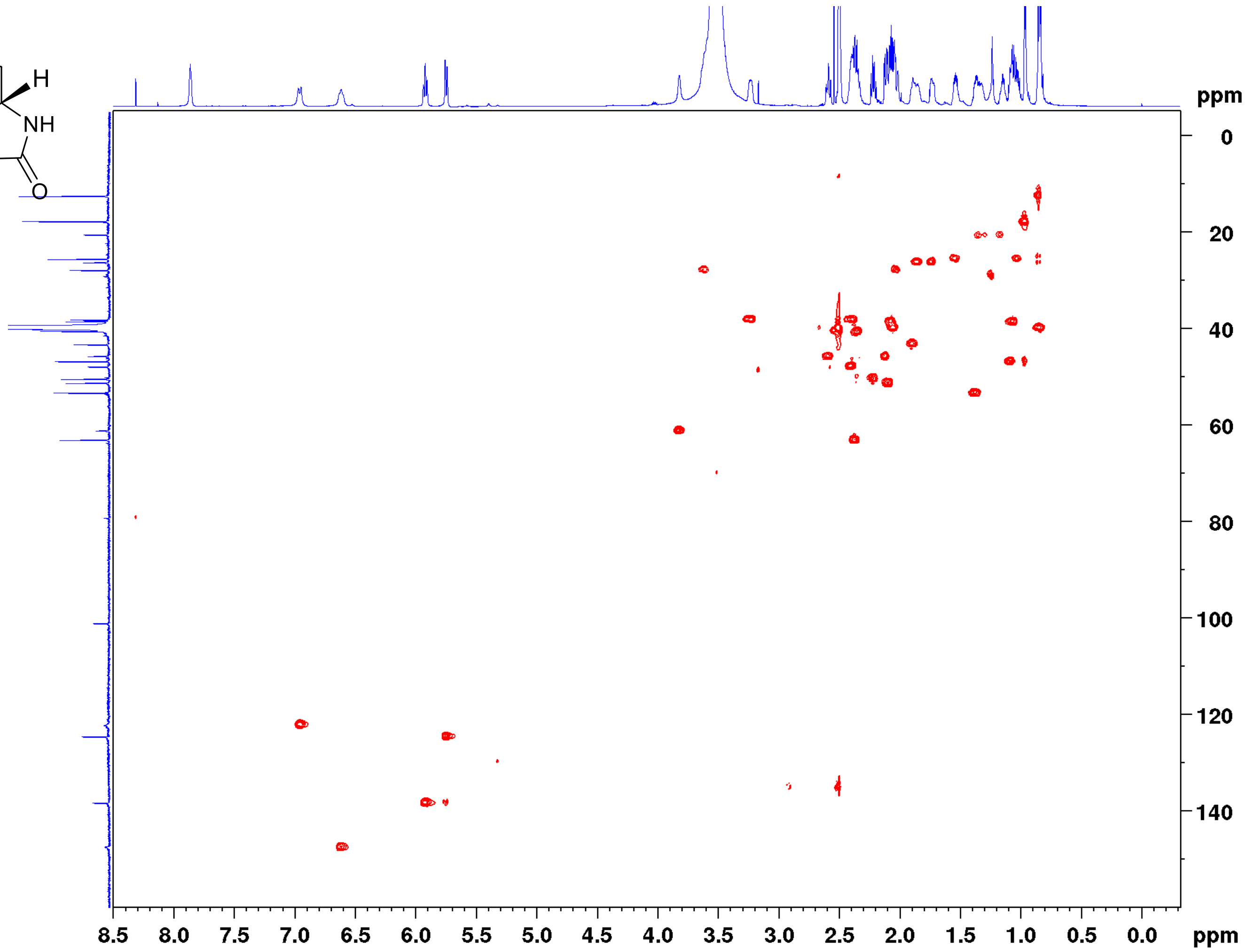

Figure S31. The HMBC spectrum of compound **4** in DMSO-d_6.

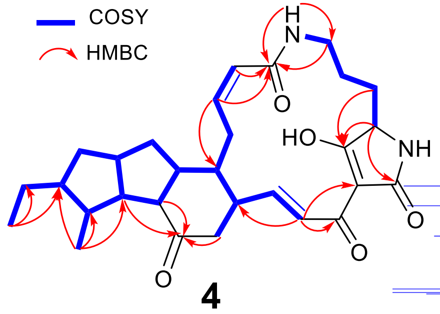

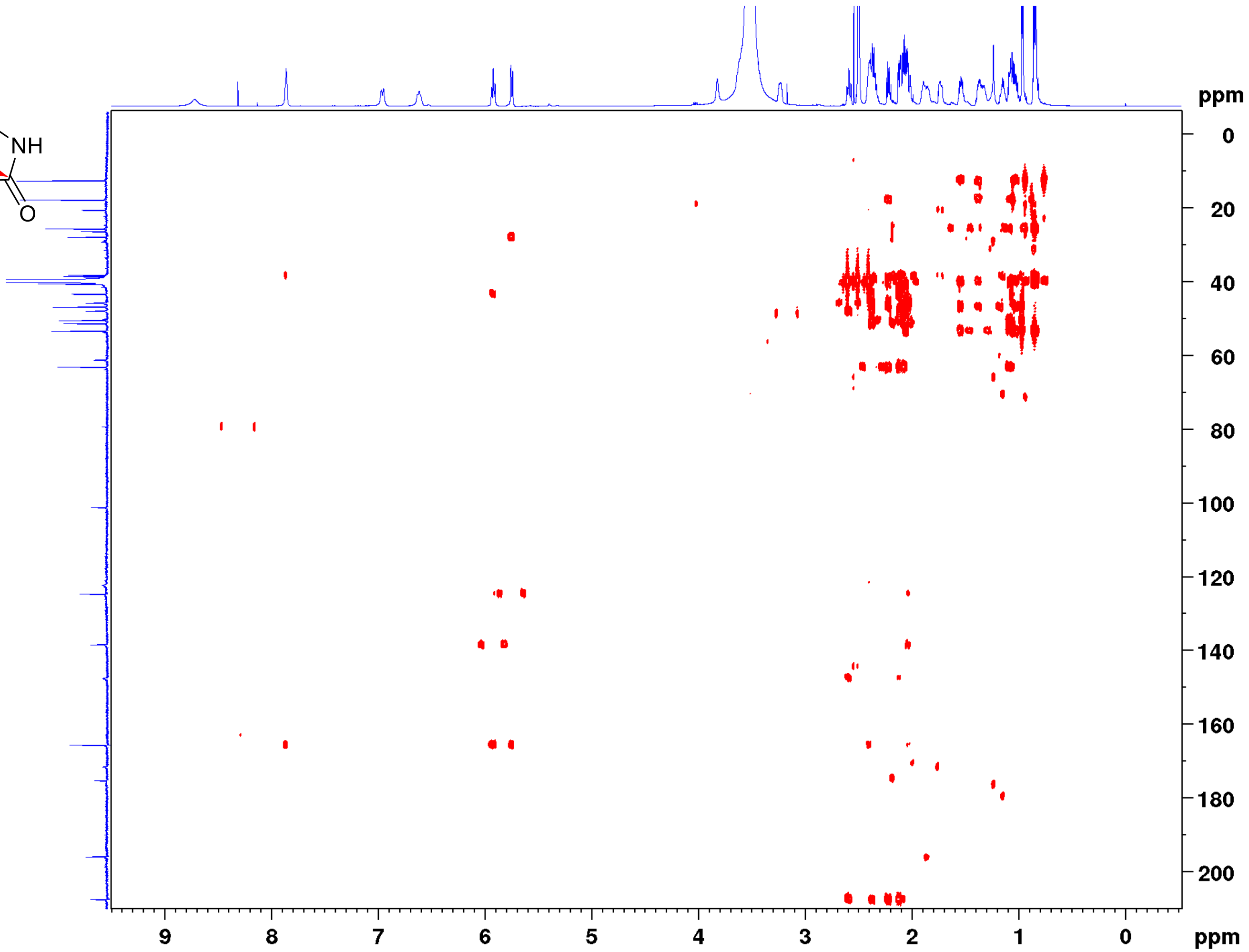

Figure S32. The ^{1}H-^{1}H COSY spectrum of compound **4** in DMSO-d_6.

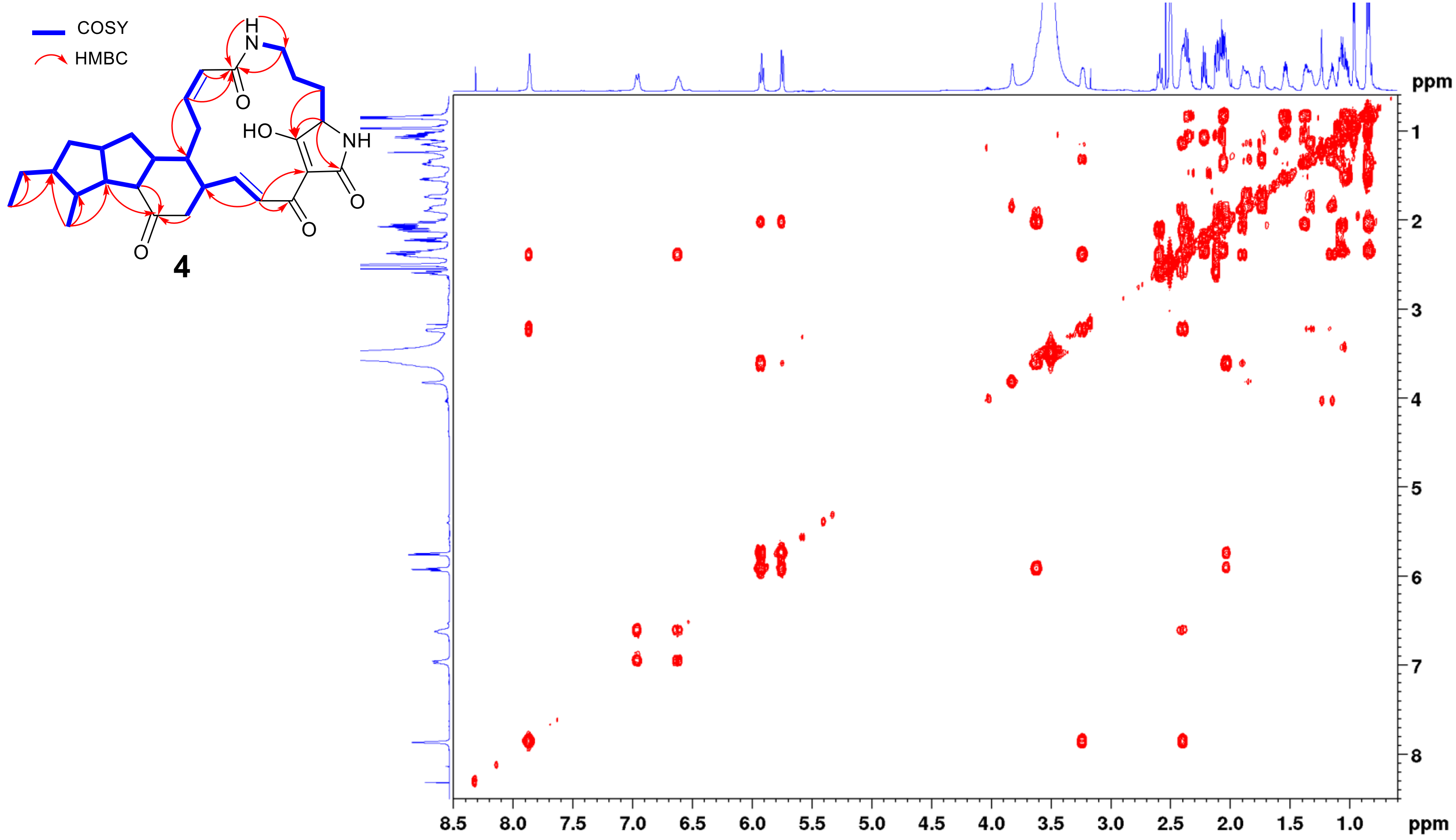

Figure S33. The NOESY spectrum of compound **4** in DMSO-d_6.

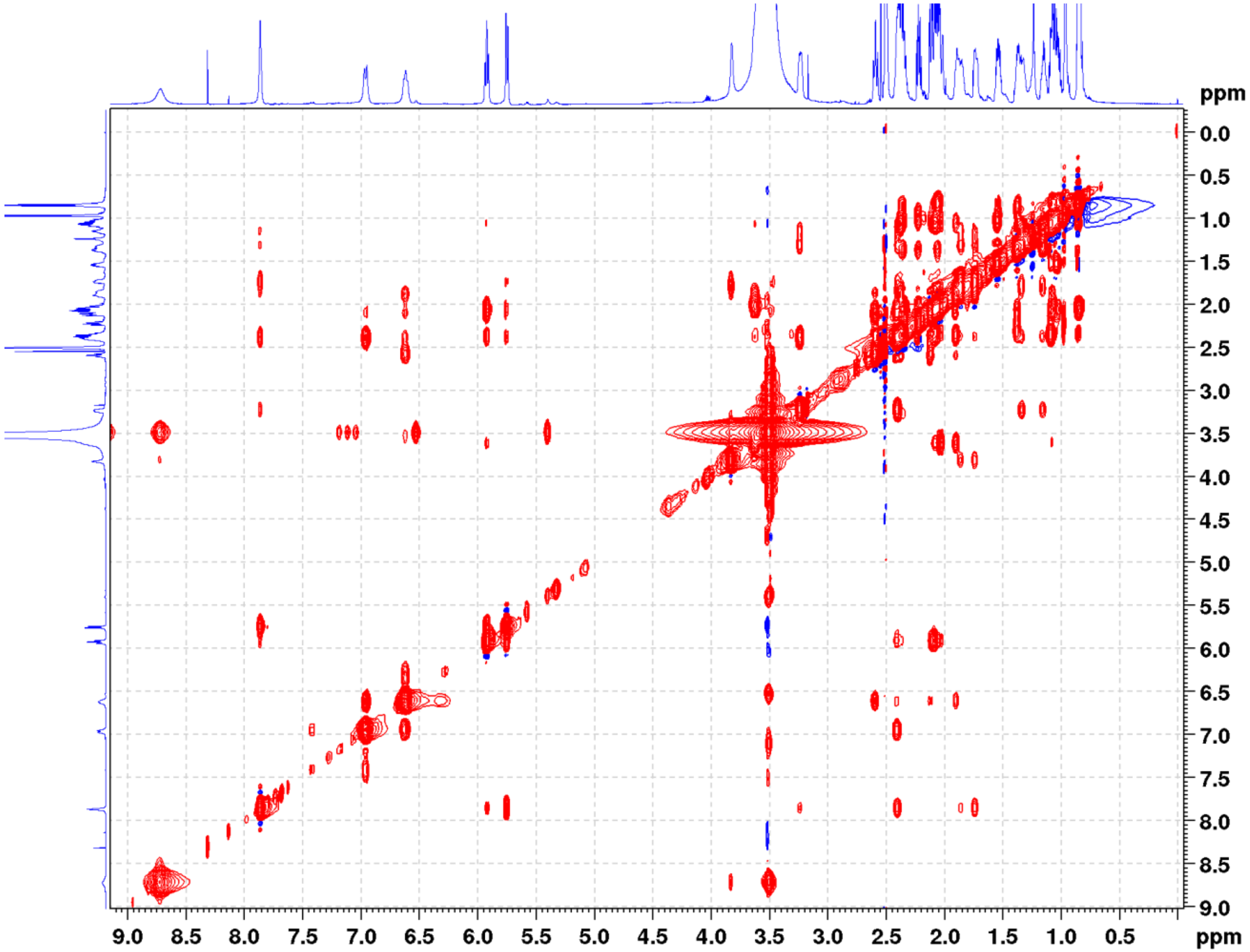

14

Figure S34. The key NOESY spectrum of compound **4** in DMSO-d_6.

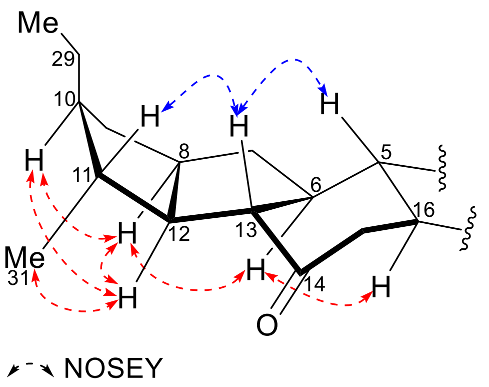

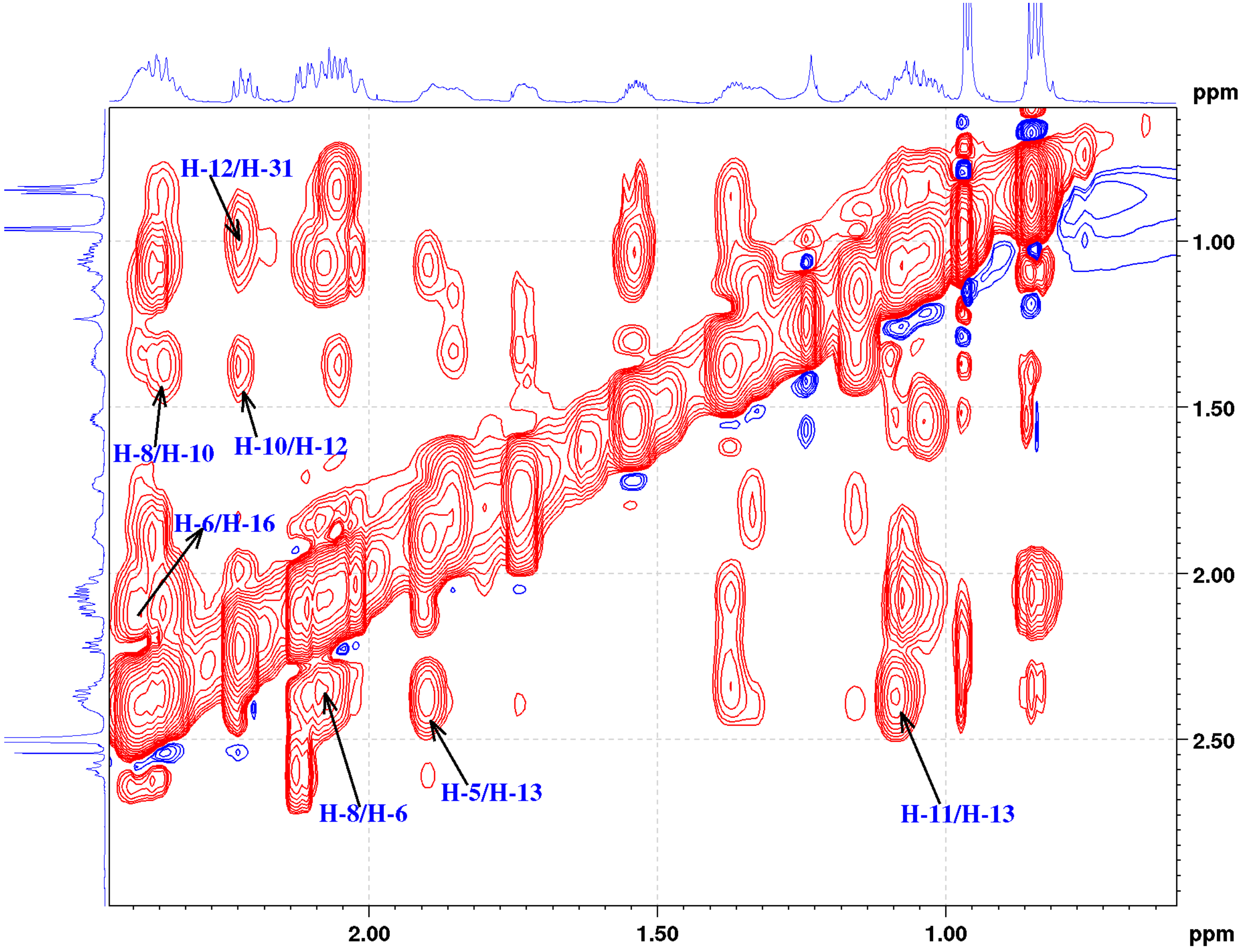

Figure S35. HRESIMS (**a**) and IR (**b**) of compound **5**.

(a) HR-ESI-MS

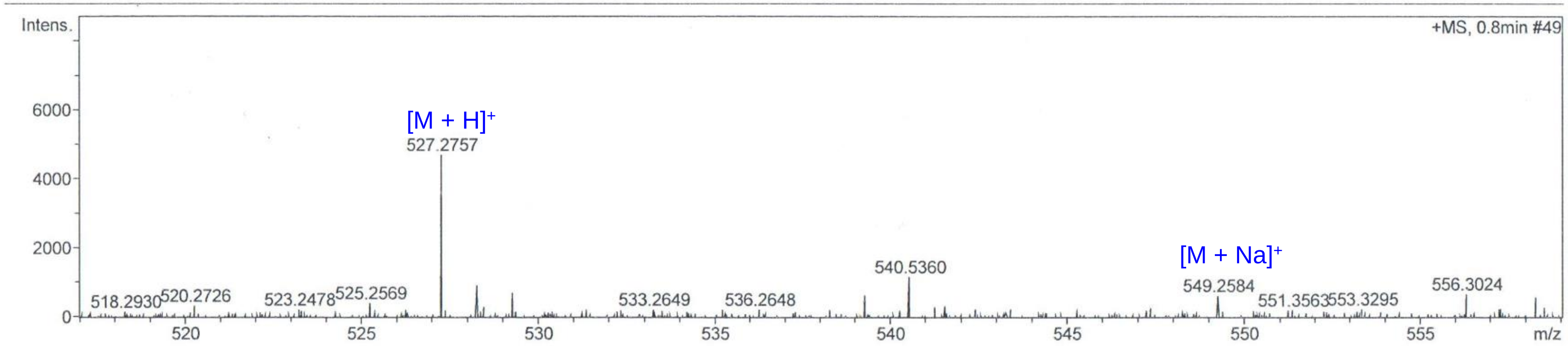

(b) IR

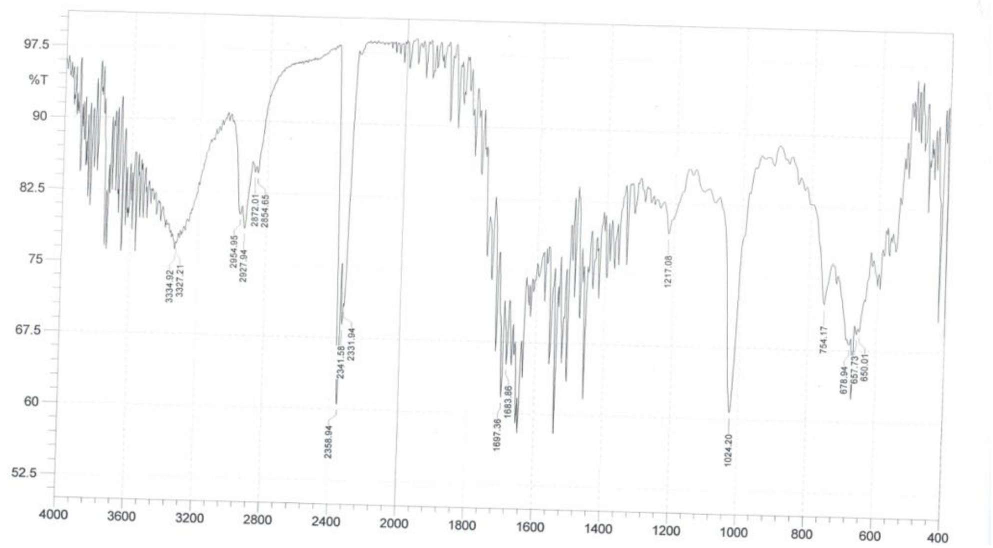

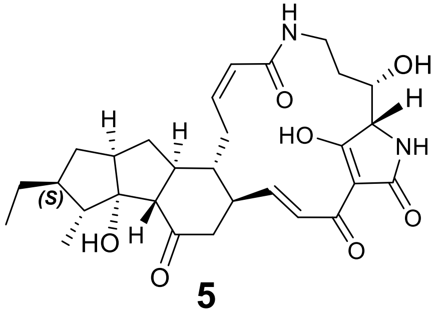

5

Chemical Formula: $C_{29}H_{38}N_2O_7$

caculated for $[M+H]^+$: 527.2757

caculated for $[M+H]^+$: 549.2577

Figure S36. The ^{1}H NMR spectrum of compound **5** in DMSO-d_6.

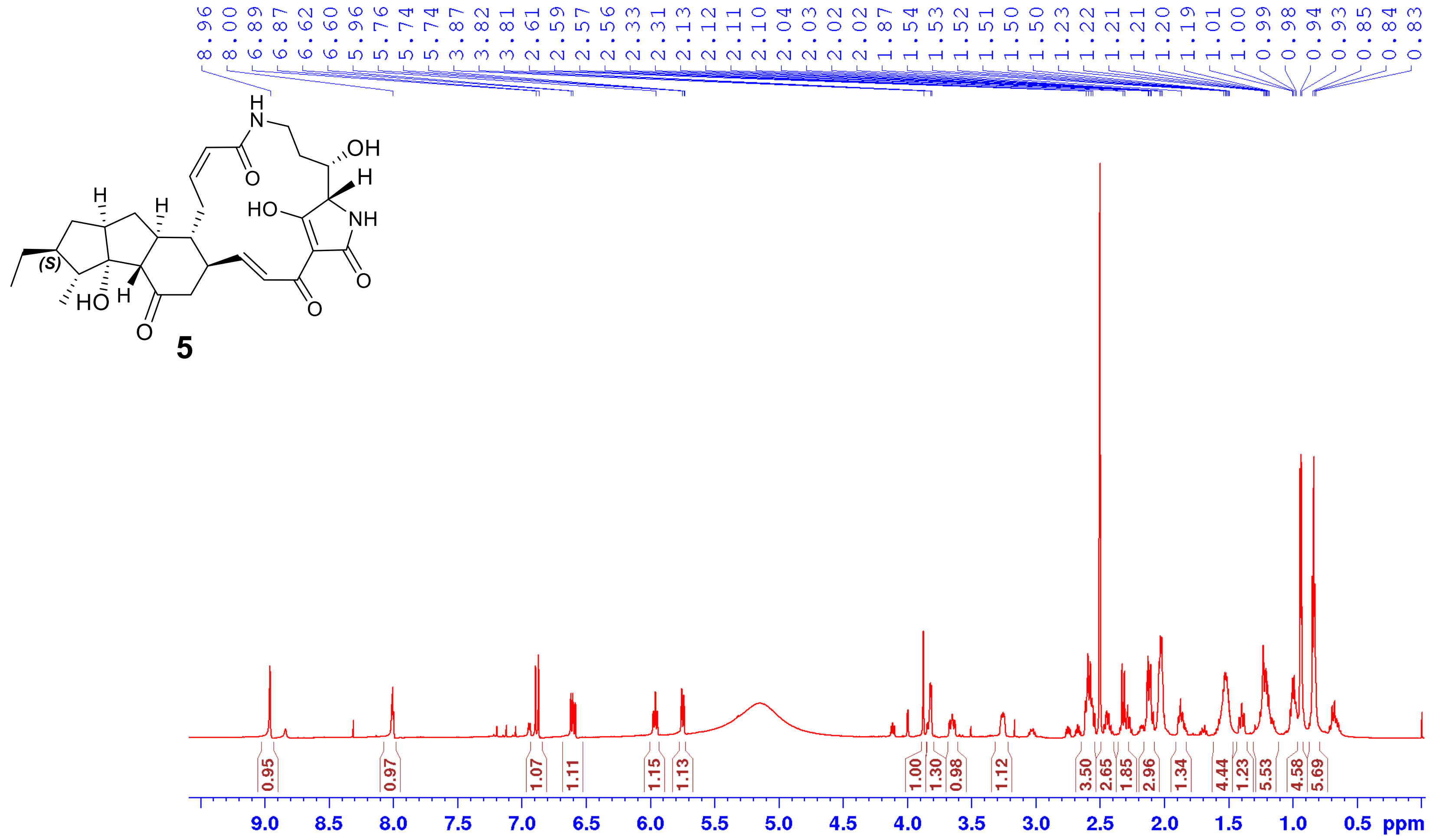

Figure S37. The ^{13}C NMR and DEPT 135 spectra of compound **5** in DMSO-d_6.

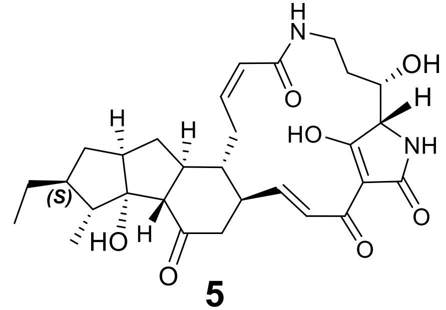

5

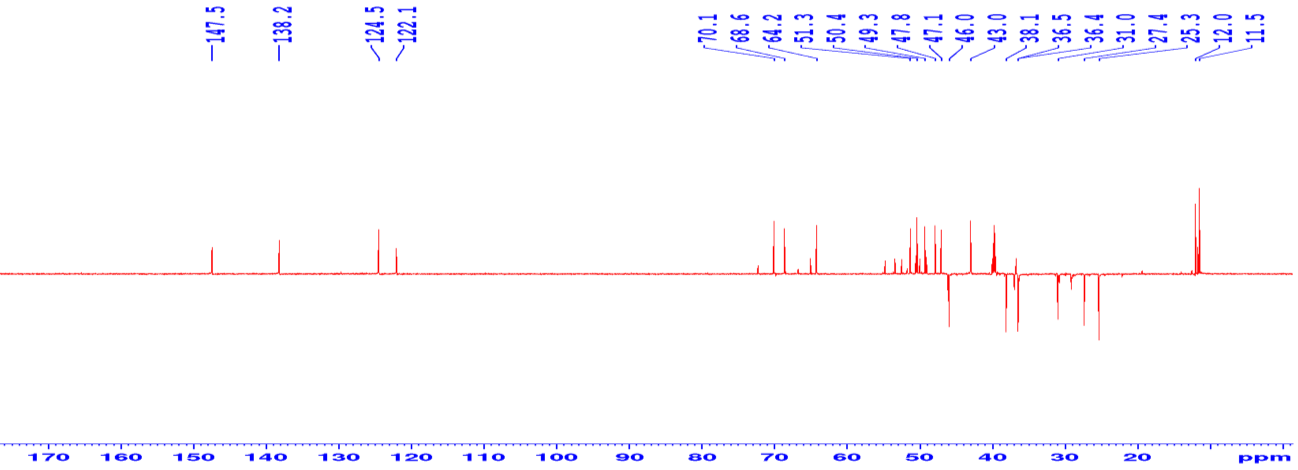

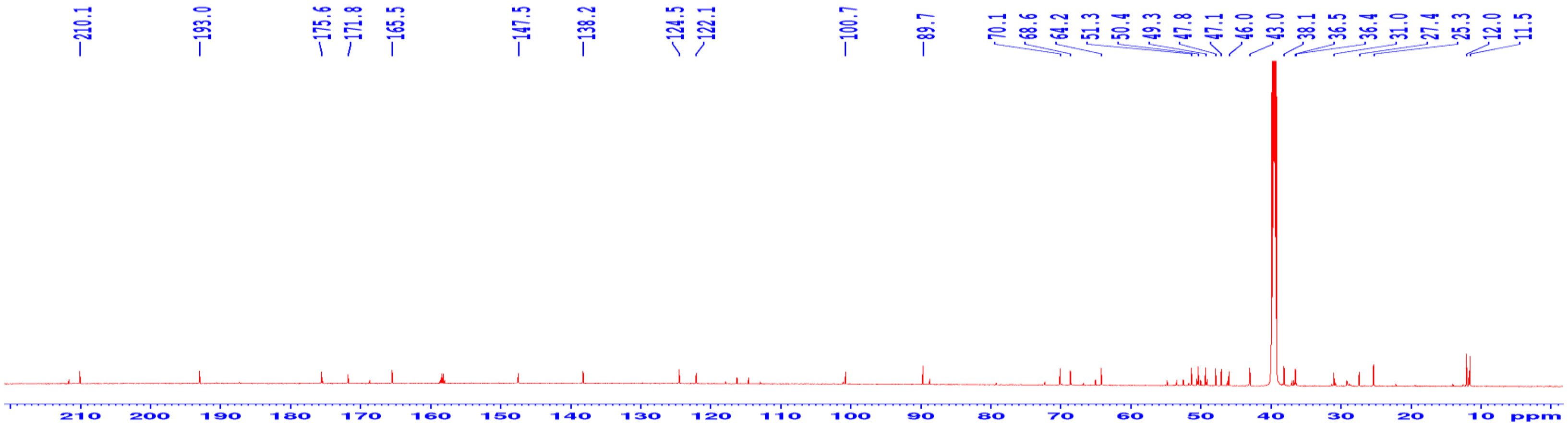

Figure S38. The HSQC spectrum of compound **5** in DMSO-d_6.

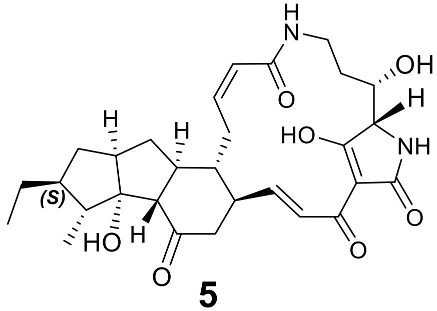

5

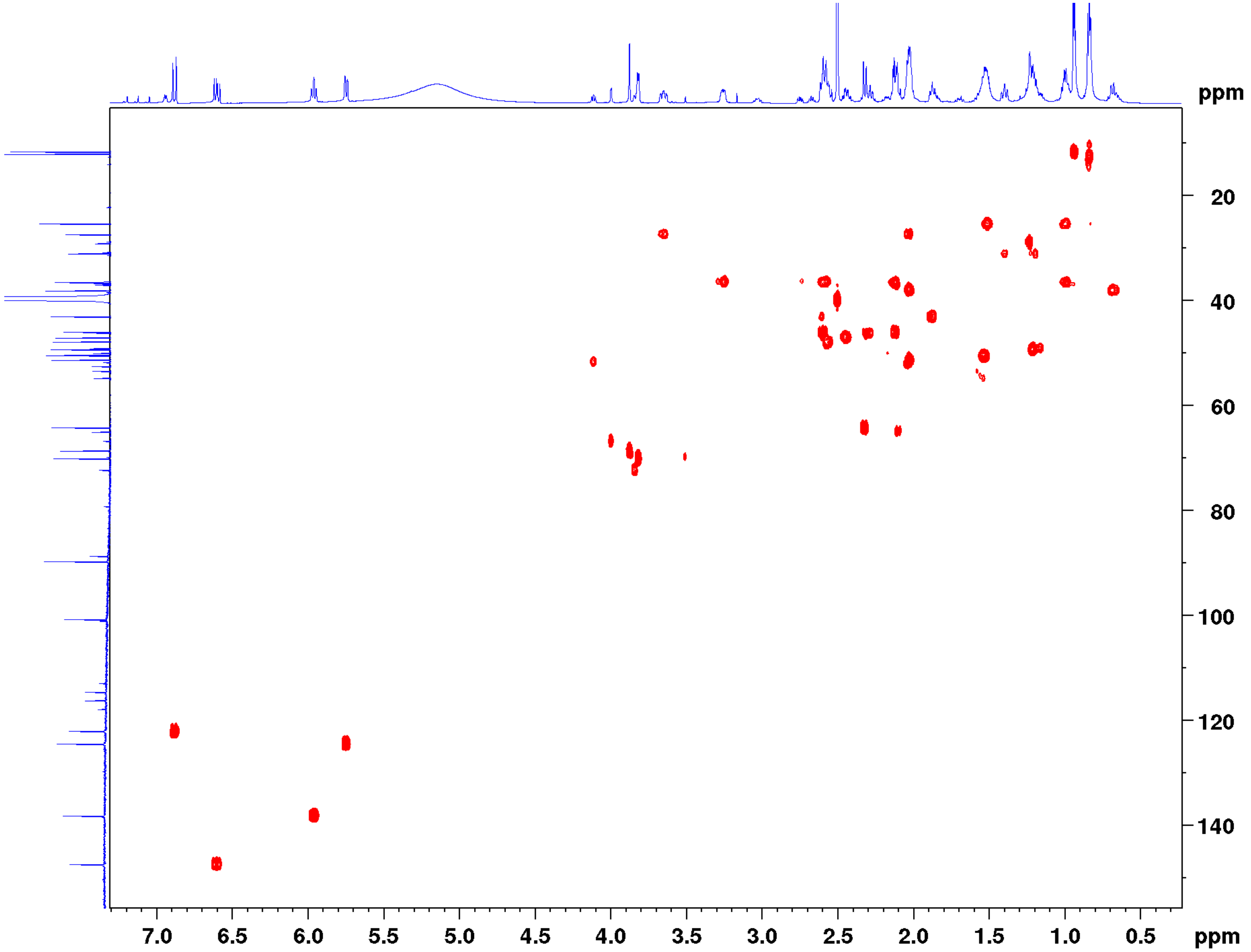

Figure S39. The HMBC spectrum of compound **5** in DMSO-d_6.

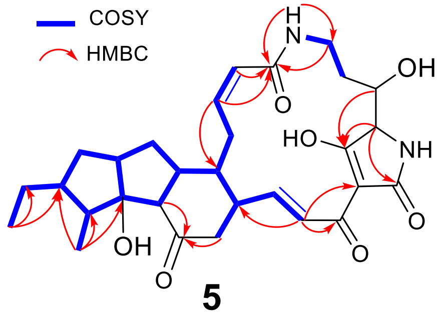

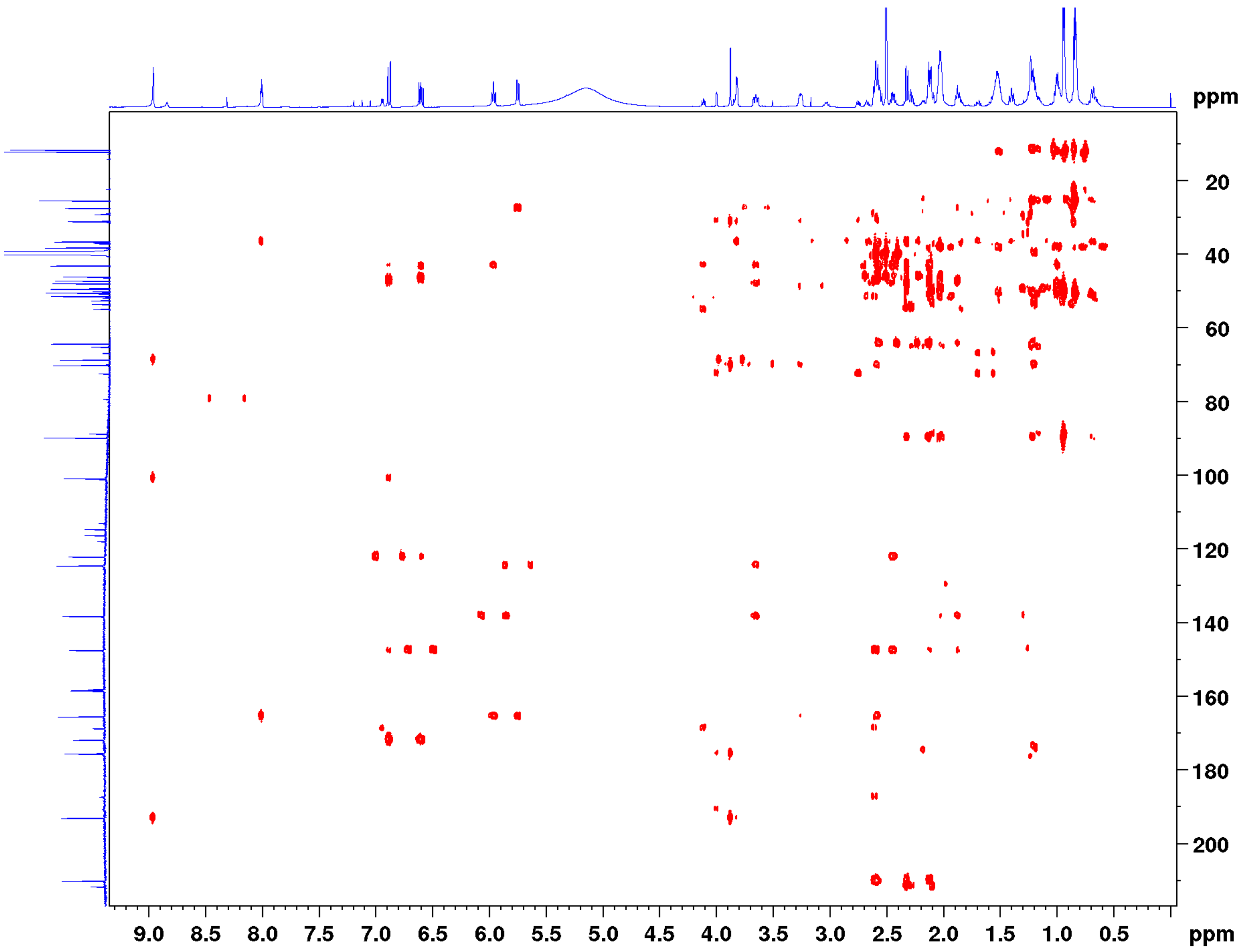

Figure S40. The ^{1}H-^{1}H COSY spectrum of compound **5** in DMSO-d_6.

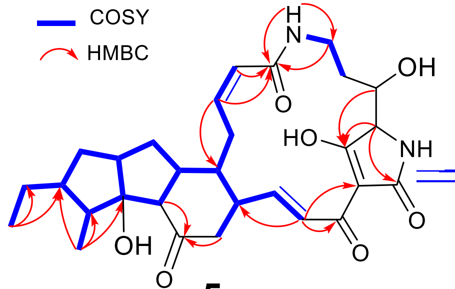

5

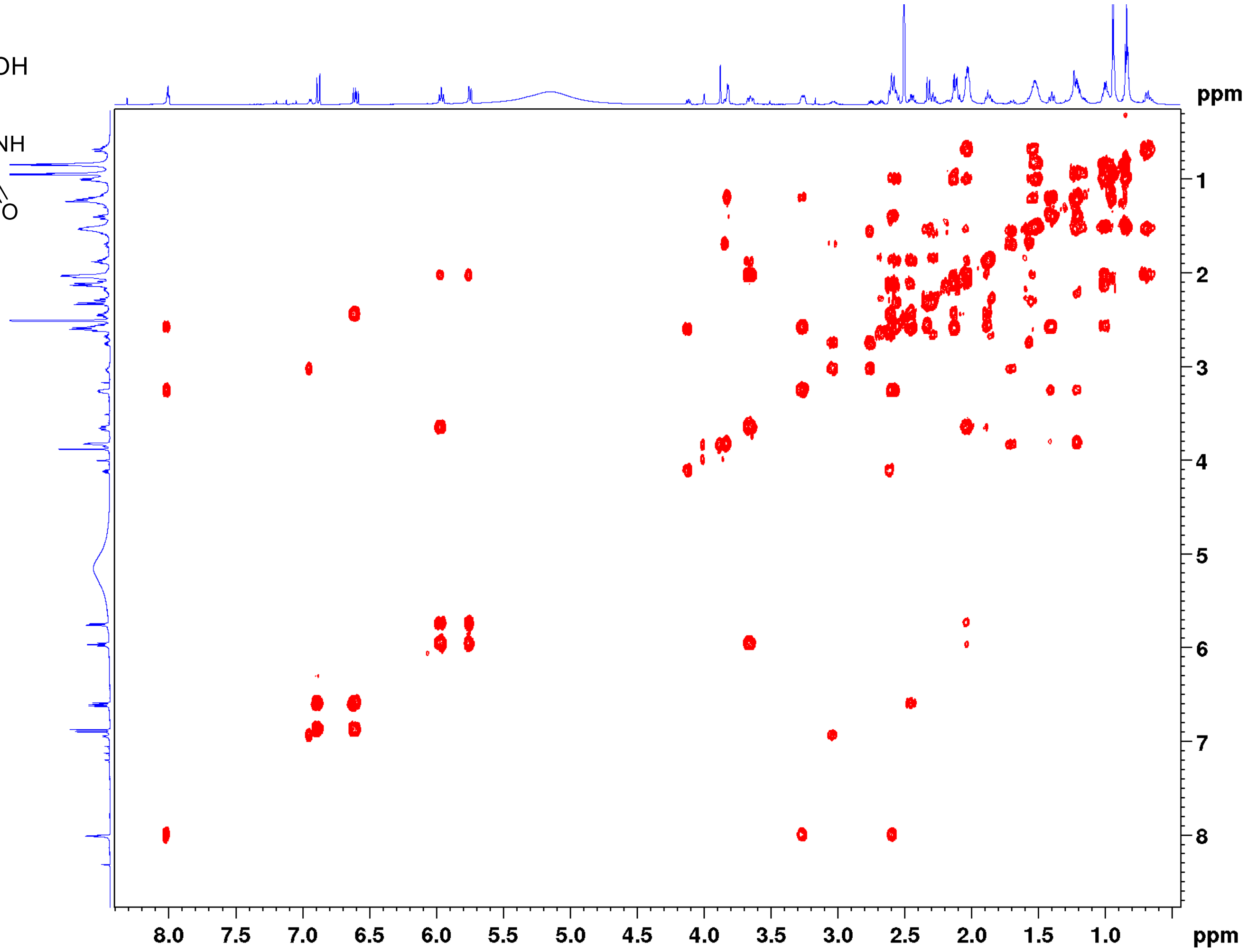

Figure S41. The NOESY spectrum of compound **5** in DMSO-d_6.

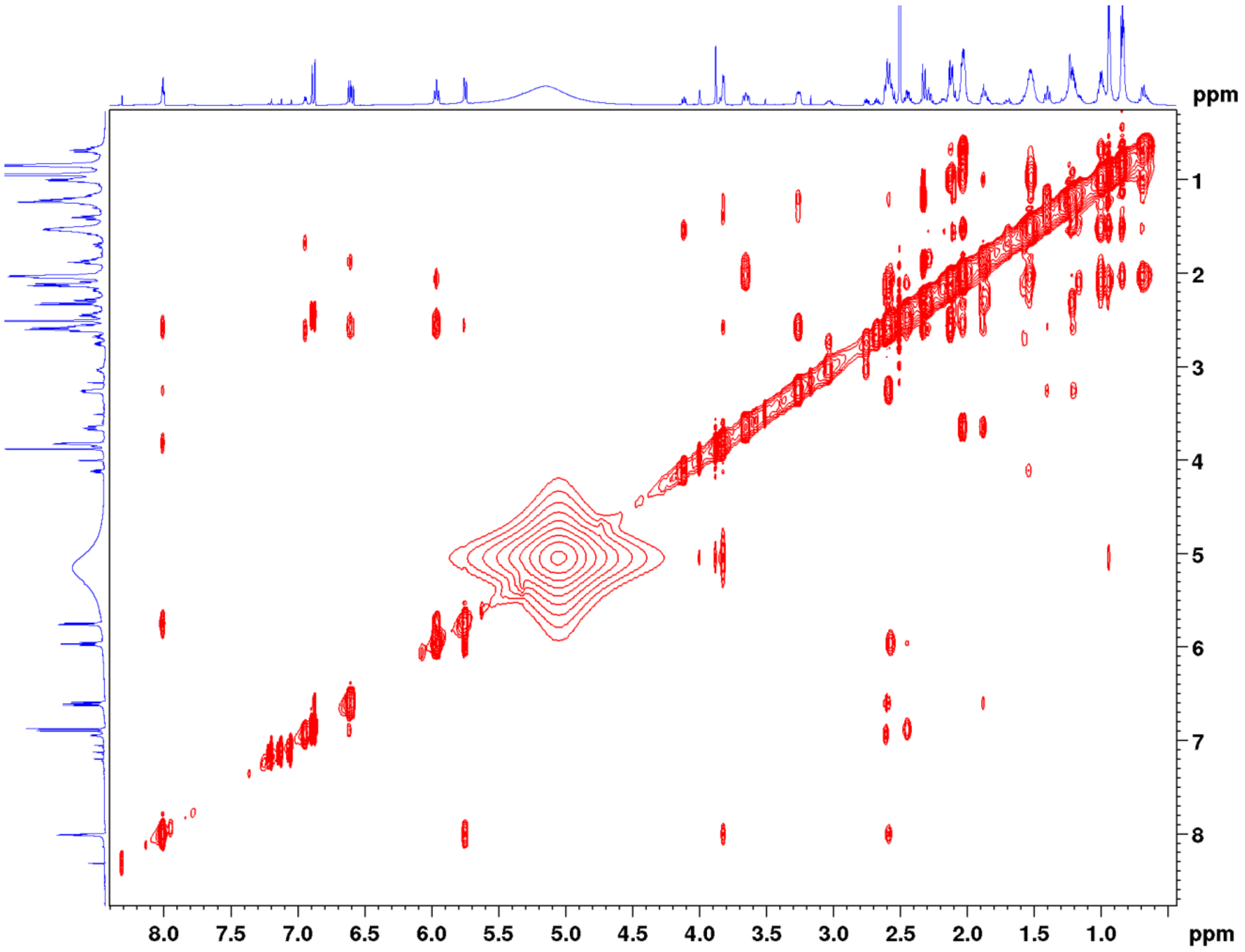

Figure S42. The key NOESY spectrum of compound **5** in DMSO-d_6.

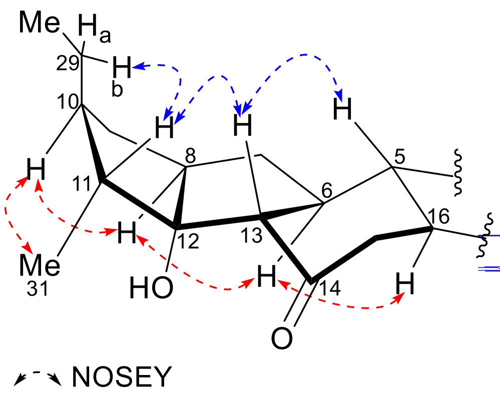

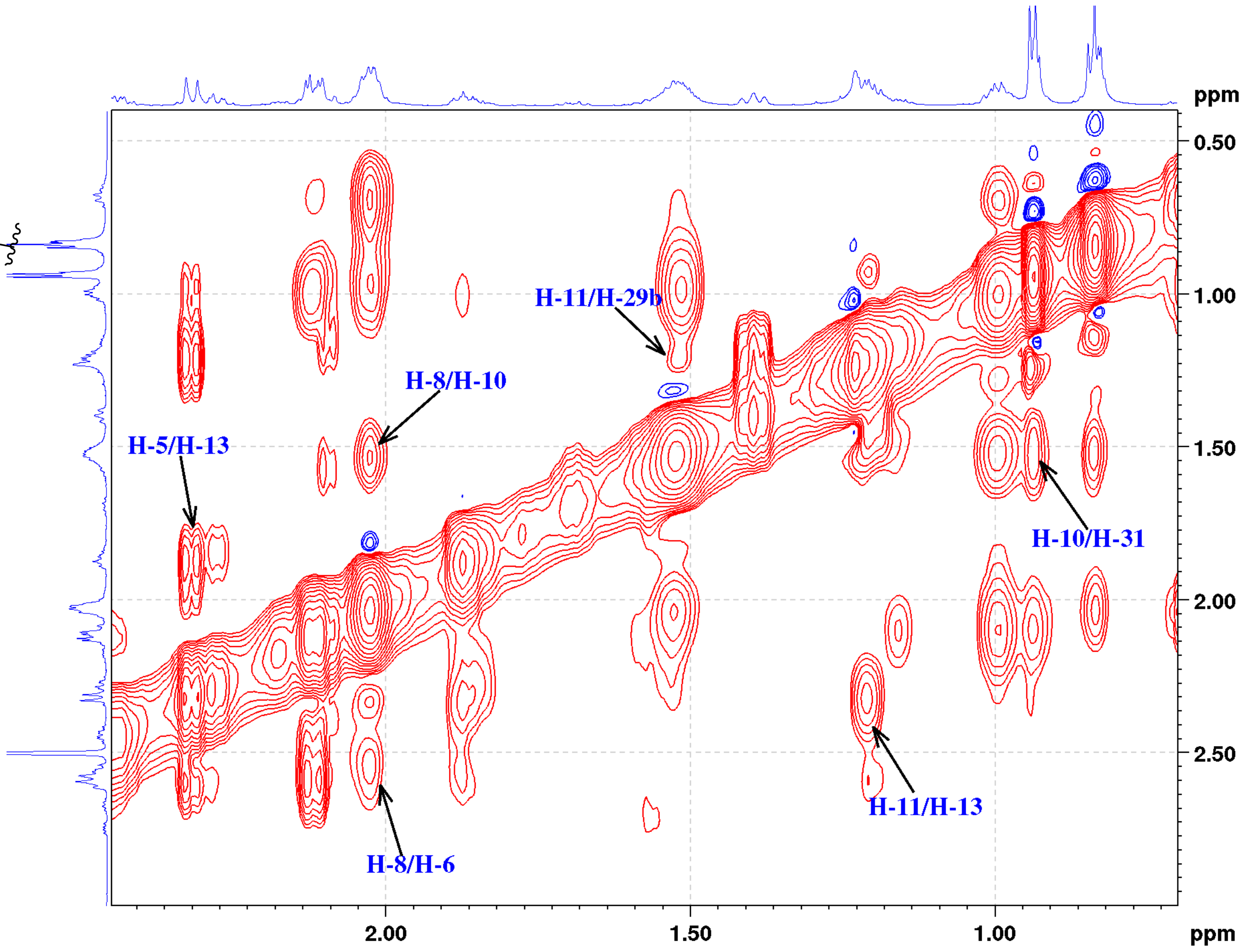

Figure S43. HRESIMS (**a**) and IR (**b**) of compound **6**.

(a) HR-ESI-MS

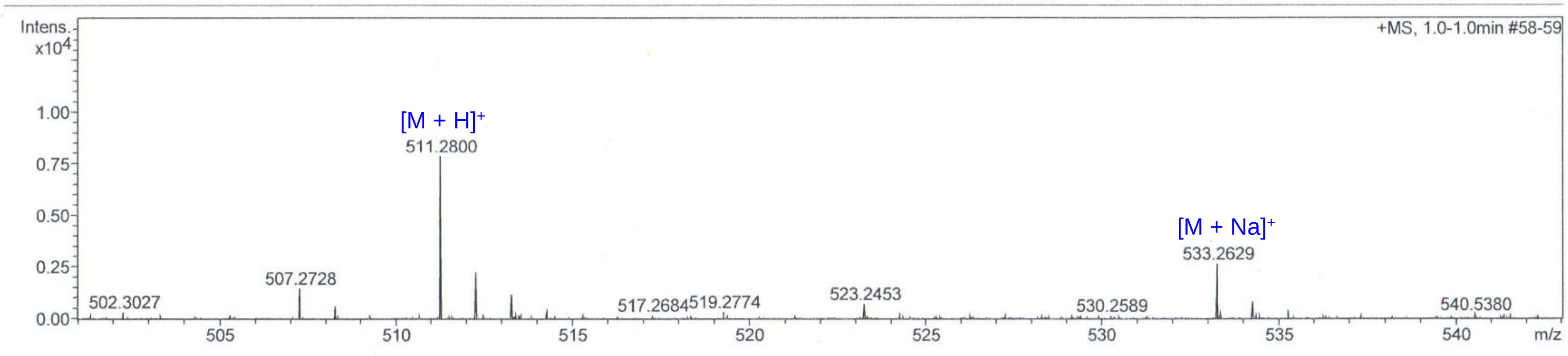

(b) IR

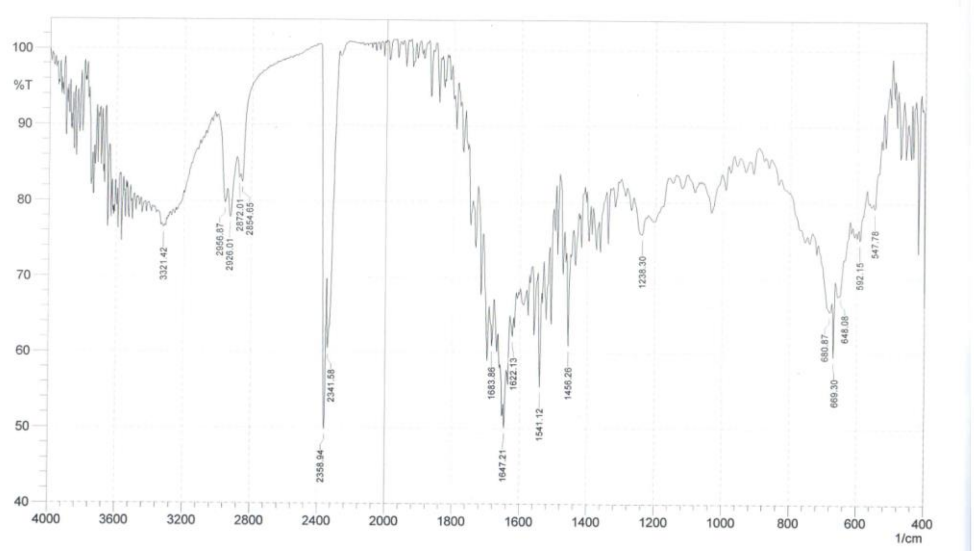

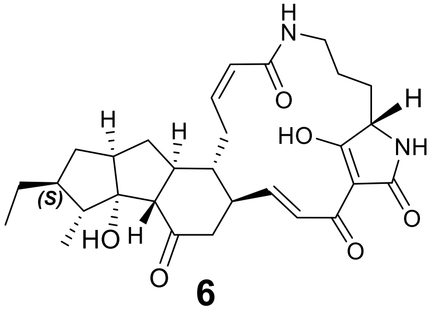

6

Chemical Formula: $C_{29}H_{38}N_2O_6$

caculated for $[M+H]^+$:511.2808

caculated for $[M+Na]^+$:533.2628

Figure S44. The ^{1}H NMR spectrum of compound **6** in DMSO-d_6.

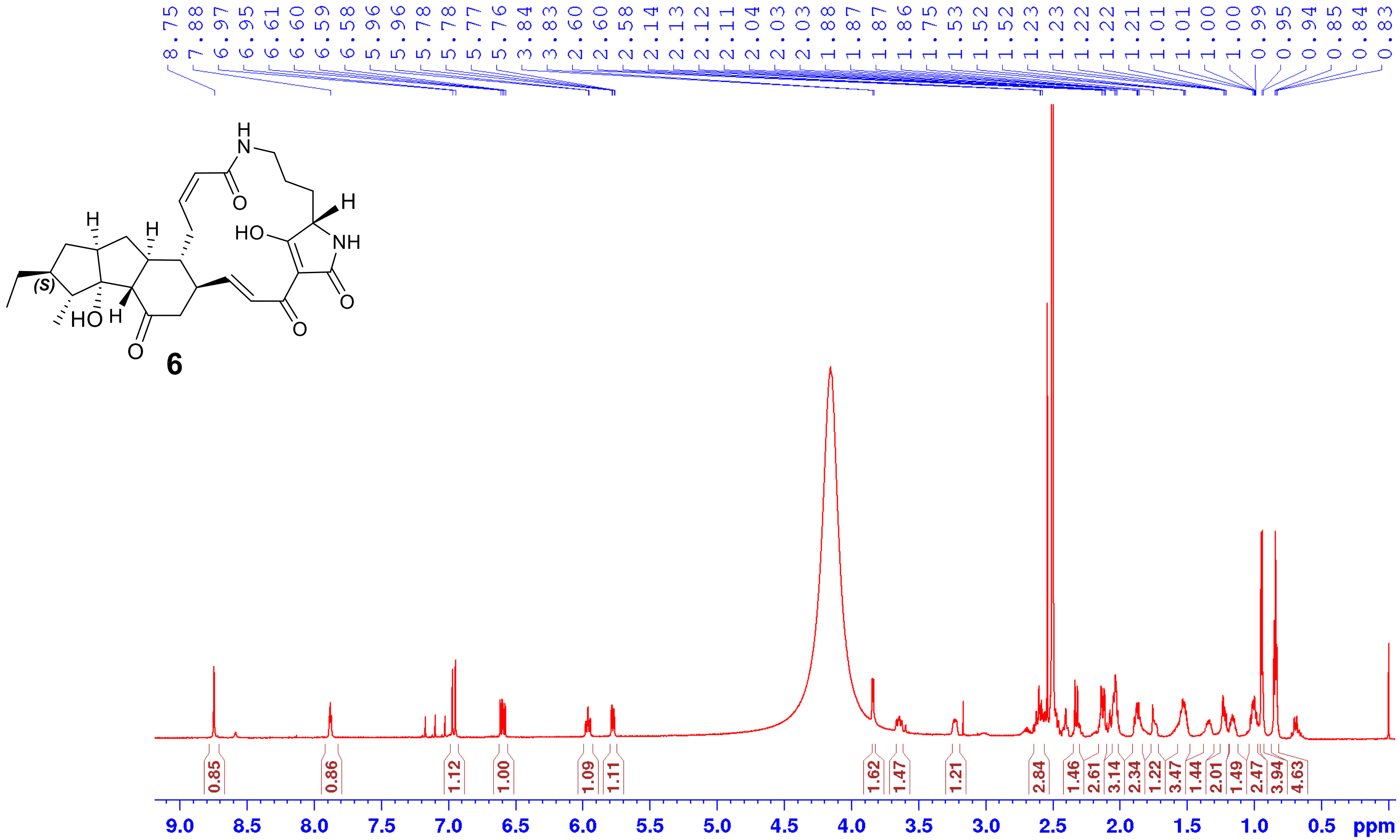

6

Figure S45. The ^{13}C NMR and DEPT 135 spectra of compound **6** in DMSO-d_6.

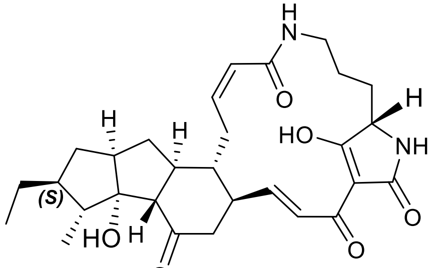

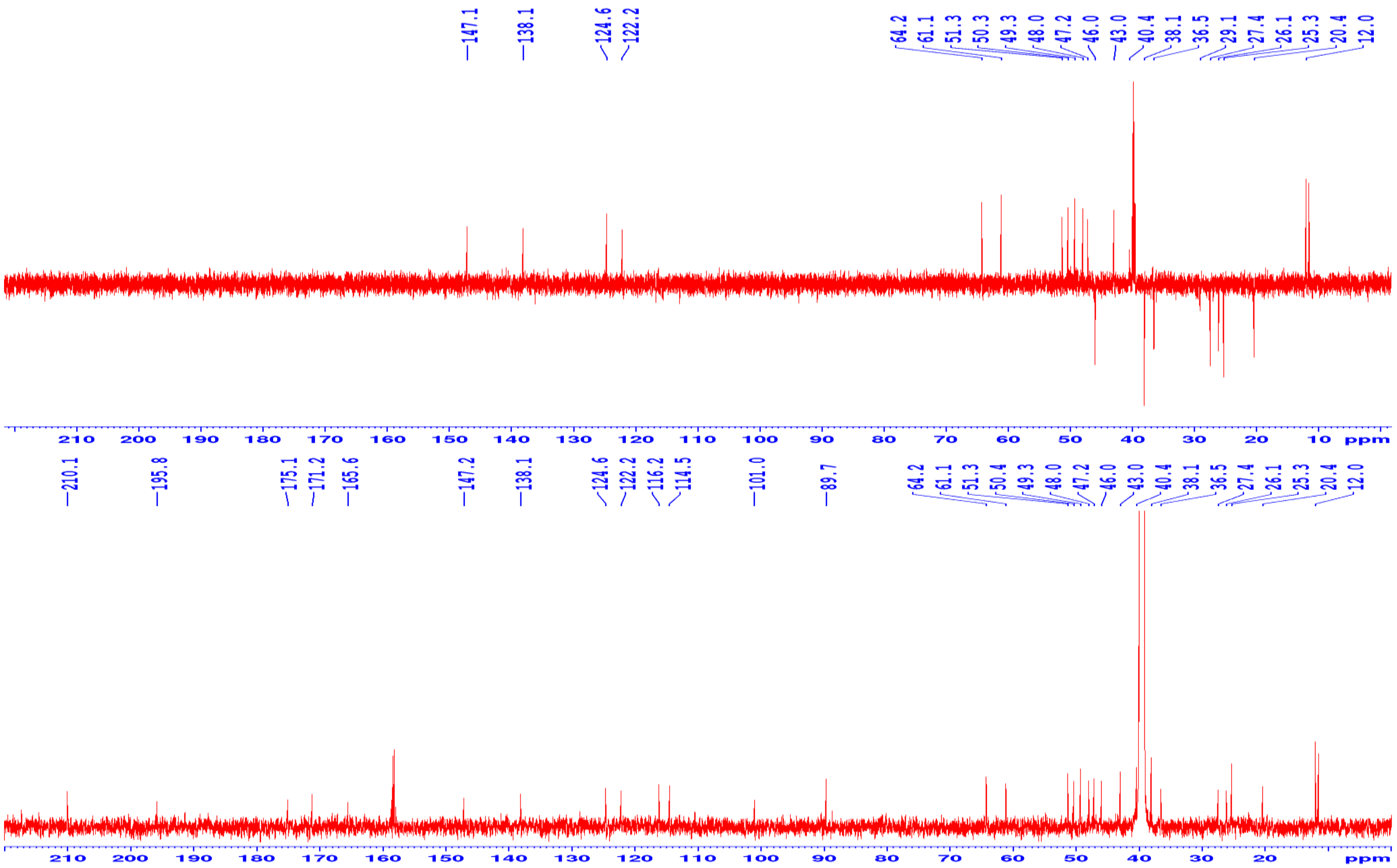
147.1
138.1
124.6
122.2
64.2
61.1
51.3
50.3
49.3
48.0
47.2
46.0
43.0
40.4
38.1
36.5
29.1
27.4
26.1
25.3
20.4
12.0
210
200
190
180
170
160
150
140
130
120
110
100
90
80
70
60
50
40
30
20
10
ppm
210.1
195.8
175.1
171.2
165.6
147.2
138.1
124.6
122.2
116.2
114.5
101.0
89.7
64.2
61.1
51.3
50.4
49.3
48.0
47.2
46.0
43.0
40.4
38.1
36.5
27.4
26.1
25.3
20.4
12.0
210
200
190
180
170
160
150
140
130
120
110
100
90
80
70
60
50
40
30
20
ppm

Figure S46. The HSQC spectrum of compound **6** in DMSO-d_6.

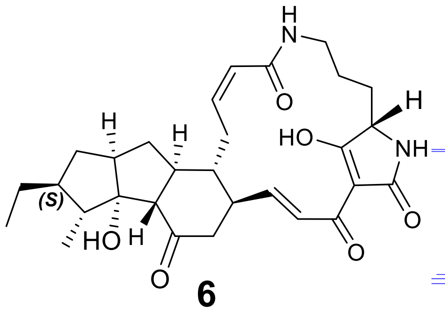

6

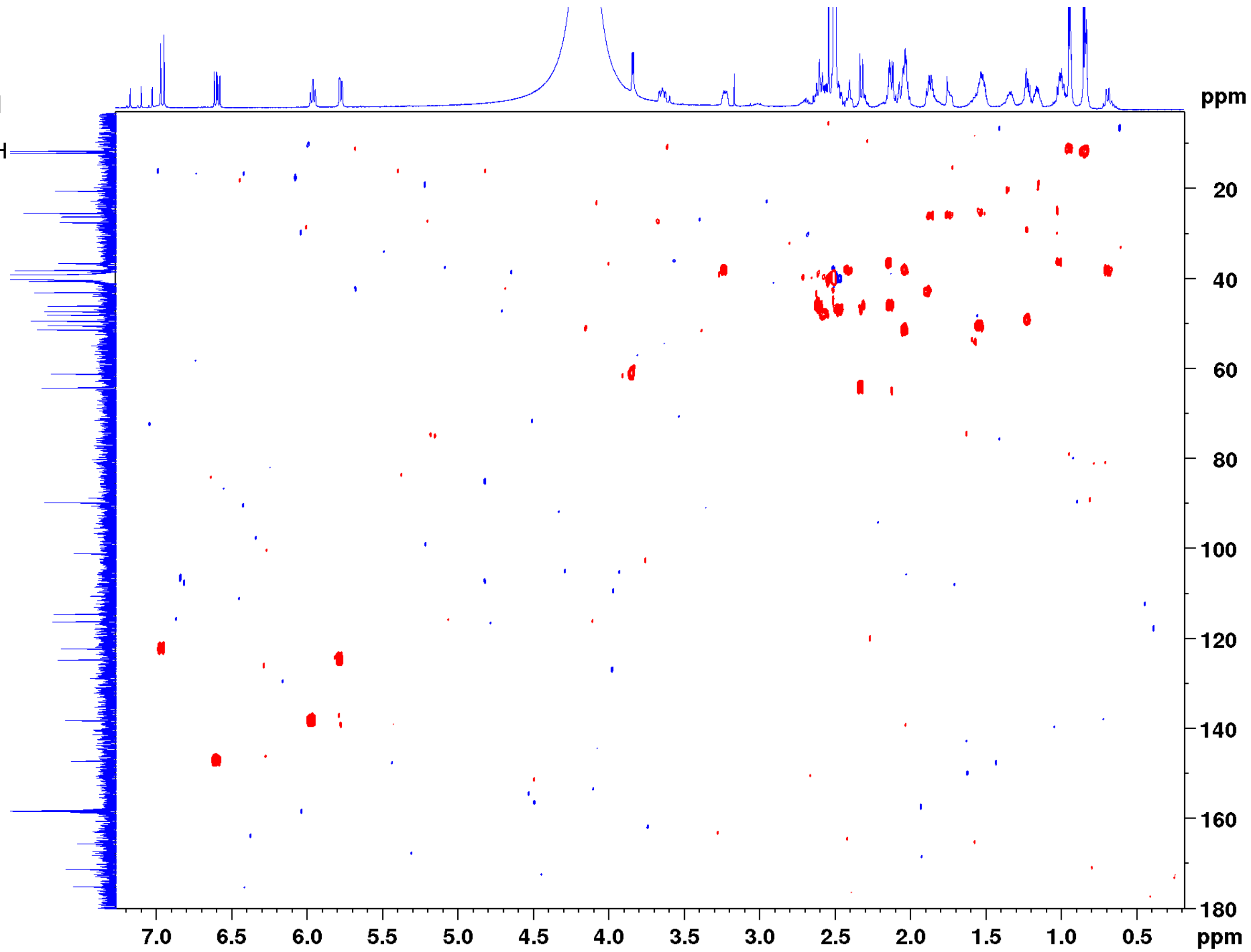

Figure S47. The HMBC spectrum of compound **6** in DMSO-d_6.

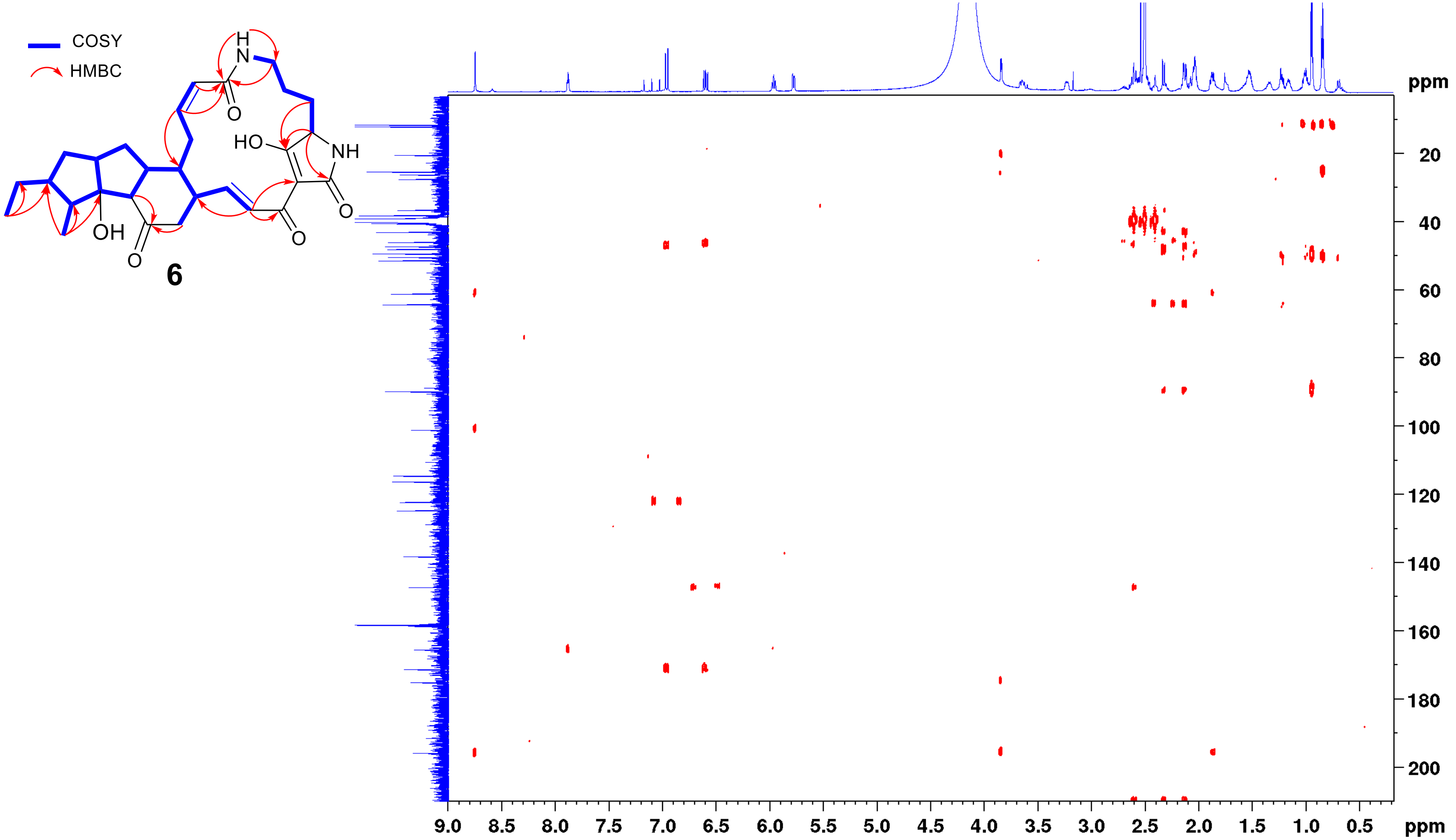

Figure S48. The ^{1}H-^{1}HCOSY spectrum of compound **6** in DMSO-d_6

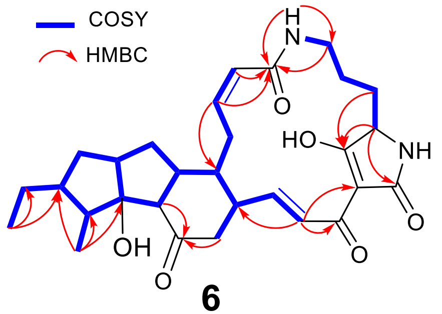

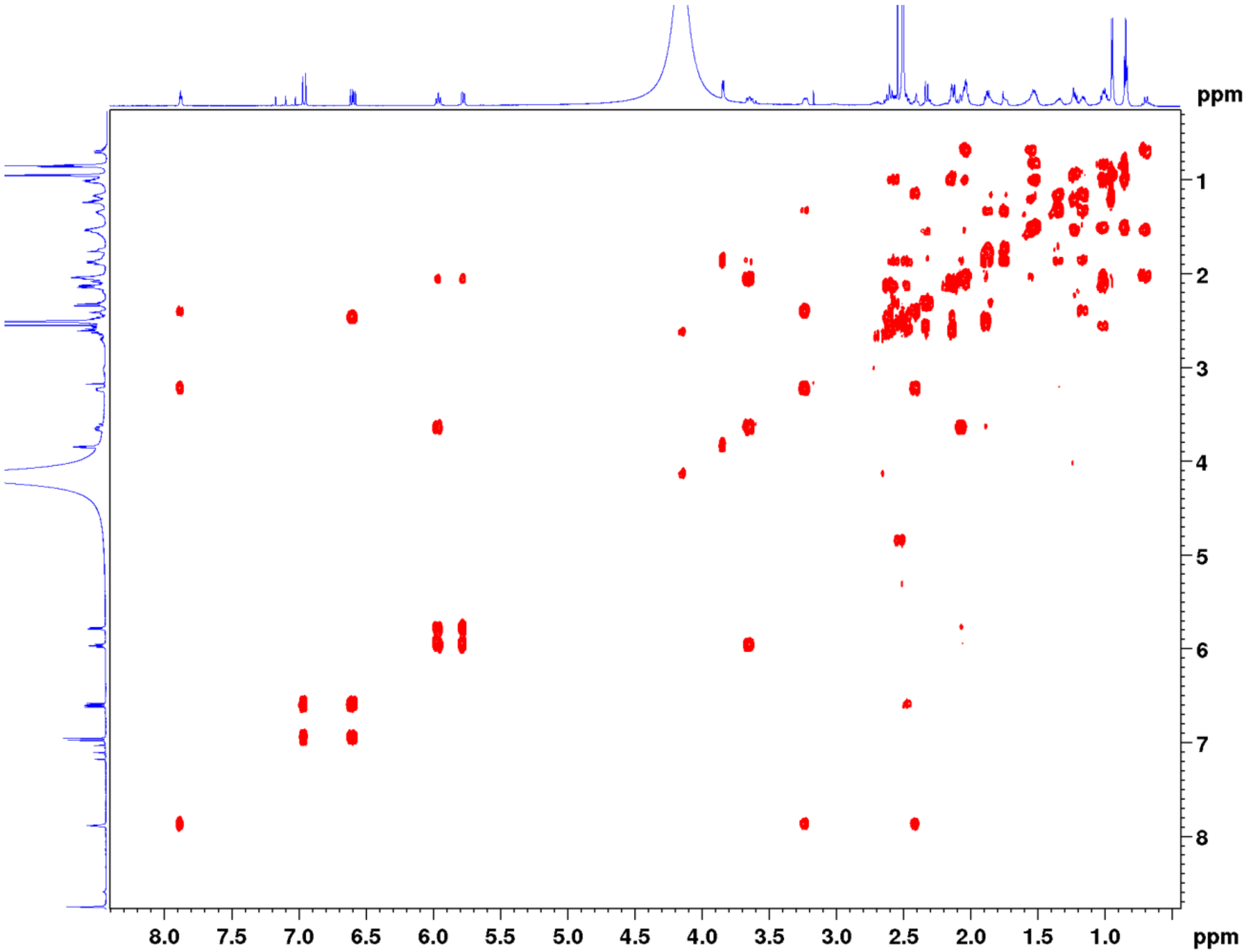

Figure S49. The NOESY spectrum of compound **6** in DMSO-d_6.

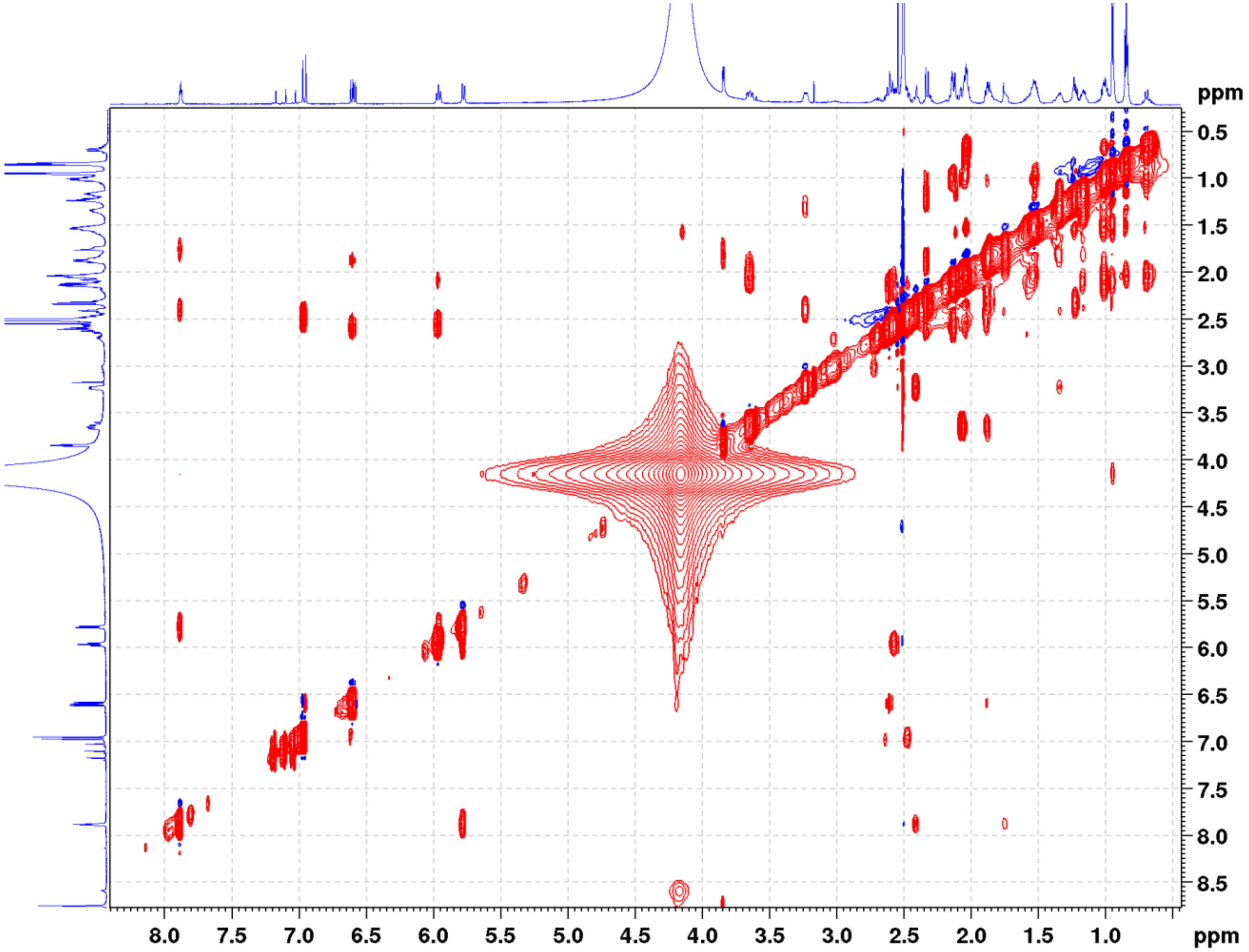

Figure S50. The key NOESY spectrum of compound **6** in DMSO-d_6.

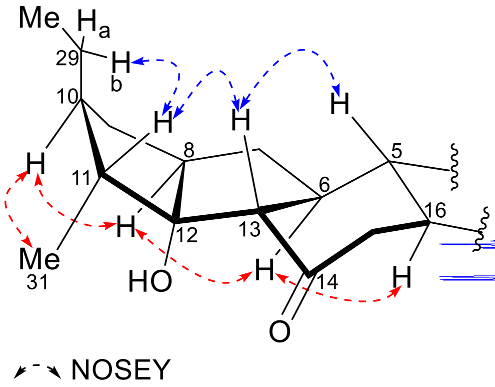

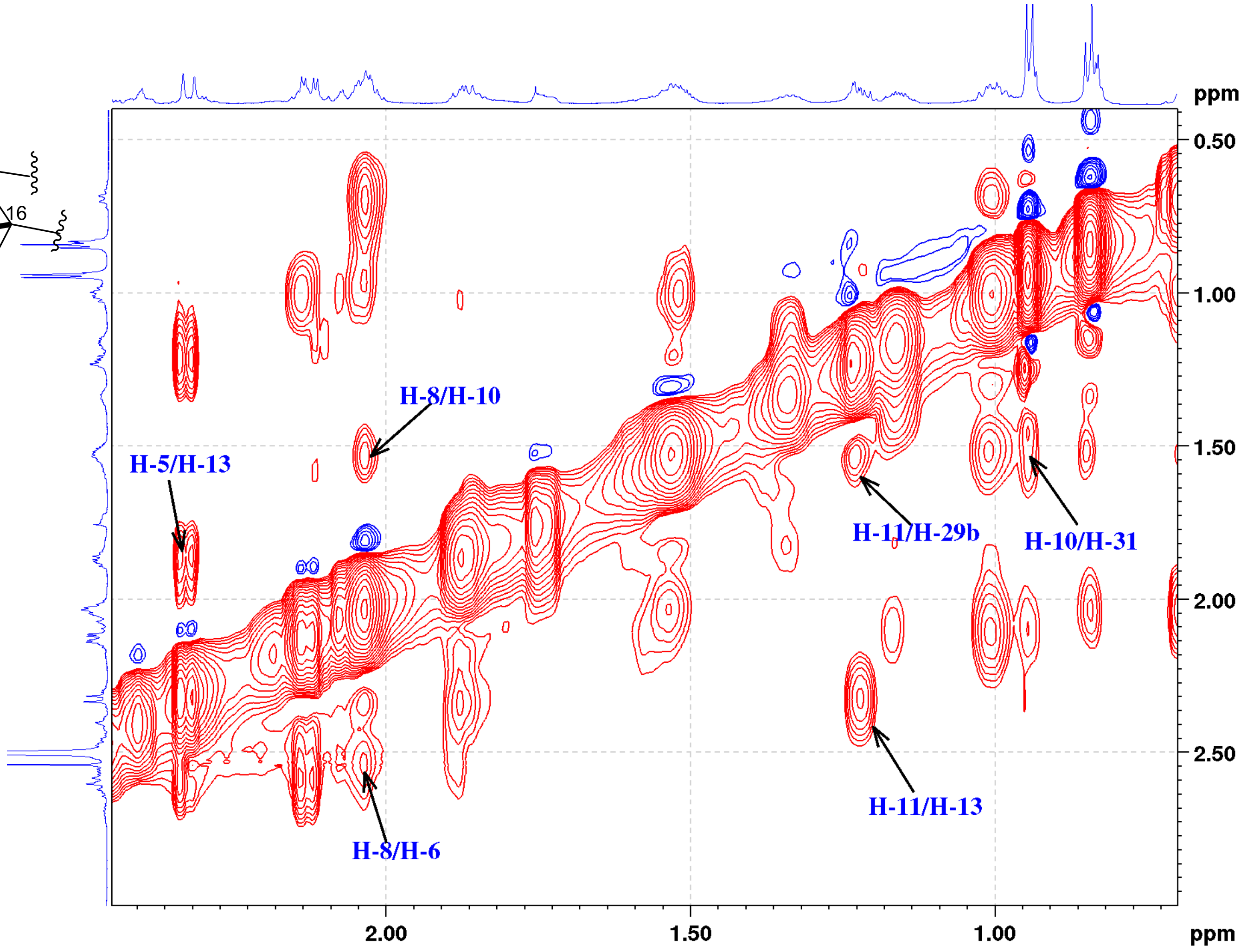

Supplement: Supplementary file 1 [file marinedrugs-17-00663-s001.pdf]
